# Co-transcriptional R-loops-mediated epigenetic regulation drives growth retardation and docetaxel chemosensitivity enhancement in advanced prostate cancer

**DOI:** 10.1186/s12943-024-01994-0

**Published:** 2024-04-24

**Authors:** Yufan Ying, Yuqing Wu, Fenghao Zhang, Yijie Tang, Jiahe Yi, Xueyou Ma, Jiangfeng Li, Danni Chen, Xiao Wang, Xiaoyan Liu, Ben Liu, Jindan Luo, Xiangyi Zheng, Liping Xie

**Affiliations:** 1 Department of Urology, First Affiliated Hospital, School of Medicine, Zhejiang University, Qingchun Road 79, Hangzhou, 310003 Zhejiang China; 2 Cancer Center, Zhejiang University, Qingchun Road 79, Hangzhou, 310003 Zhejiang China; 3 First Affiliated Hospital, School of Medicine, Zhejiang University, Hangzhou, Zhejiang China; 4 Department of Pathology, First Affiliated Hospital, School of Medicine, Zhejiang University, Hangzhou, Zhejiang China

**Keywords:** M^6^A, DNA methylation, Prostate cancer, R-loops

## Abstract

R-loops are prevalent three-stranded nucleic acid structures, comprising a DNA-RNA hybrid and a displaced single-stranded DNA, that frequently form during transcription and may be attributed to genomic stability and gene expression regulation. It was recently discovered that RNA modification contributes to maintain the stability of R-loops such as N6-methyladenosine (m^6^A). Yet, m^6^A-modified R-loops in regulating gene transcription remains poorly understood. Here, we demonstrated that insulin-like growth factor 2 mRNA-binding proteins (IGF2BPs) recognize R-loops in an m^6^A-dependent way. Consequently, IGF2BPs overexpression leads to increased overall R-loop levels, cell migration inhibition, and cell growth retardation in prostate cancer (PCa) via precluding the binding of DNA methyltransferase 1(DNMT1) to semaphorin 3 F (SEMA3F) promoters. Moreover, the K homology (KH) domains of IGF2BPs are required for their recognition of m^6^A-containing R-loops and are required for tumor suppressor functions. Overexpression of SEMA3F markedly enhanced docetaxel chemosensitivity in prostate cancer via regulating Hippo pathway. Our findings point to a distinct R-loop resolution pathway mediated by IGF2BPs, emphasizing the functional importance of IGF2BPs as epigenetic R-loop readers in transcriptional genetic regulation and cancer biology.

The manuscript summarizes the new role of N6-methyladenosine in epigenetic regulation, we introduce the distinct R-loop resolution mediated by IGF2BP proteins in an m6A-dependent way, which probably lead to the growth retardation and docetaxel chemotherapy resistance in prostate cancer. Moreover, our findings first emphasized the functional importance of IGF2BPs as epigenetic R-loop readers in transcriptional genetic regulation and cancer biology. In addition, our research provides a novel RBM15/IGF2BPs/DNMT1 trans-omics regulation m6A axis, indicating the new crosstalk between RNA m6A methylation and DNA methylation in prostate cancer.

**Graphical Abstract:**

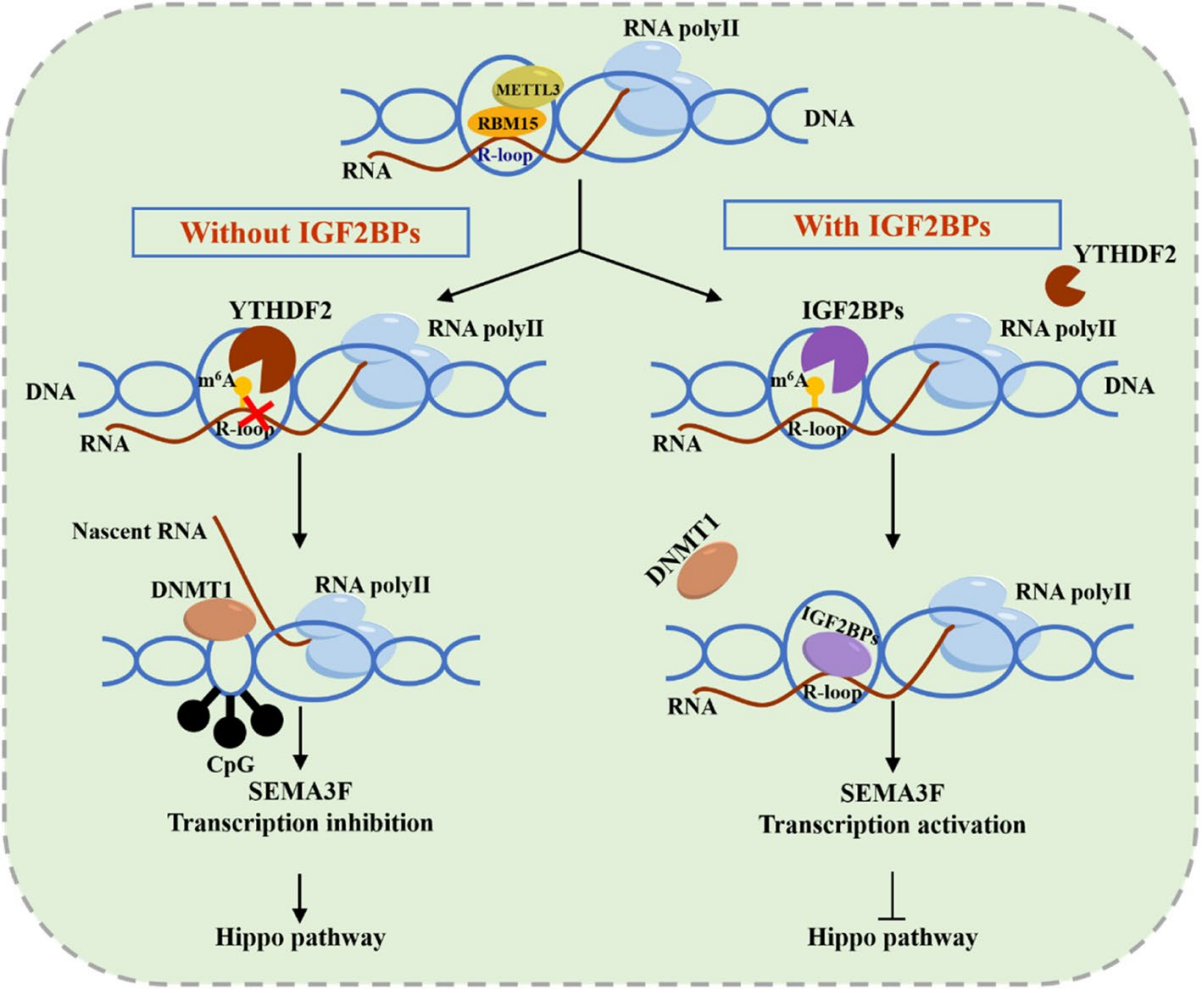

**Supplementary Information:**

The online version contains supplementary material available at 10.1186/s12943-024-01994-0.

## Introduction

R-loops have typically been recognized as harmful consequences of transcription that contribute to genomic instability over the last decade [1]. Broadly, R-loops are divided into two categories: physiological and pathological [2]. Physiological R loops often emerge as a result of a pre-programmed process involving particular elements, whereas pathological R-loops occur mistakenly and abruptly. R-loops have lately been highlighted in biomedical research as being important in cellular processes such as gene control and homologous recombination repair [3–6]. Based on the accumulated evidence for the abundance of physiological R-loops in gene promoters and termination regions, it appears that the presence of physiological R-loops may contribute to gene expression regulation. In mammalian cells, R-loops have been linked to the unmethylated state of CpG islands presenting at promoters of numerous genes via preventing the binding of DNA methyltransferase (DNMTs) and attracting ten-eleven translocation (TET) DNA demethylases [7, 8].

As the most abundant RNA modification, N6-methyladenosine (M^6^A) directs a broad range of crucial post-transcriptional cellular processes [9–11]. A growing body of evidence has uncovered m^6^A’s novel role in R-loop development and resolution [12–15]. Depletion of METTL3 lead to the reduction of R-loop accumulation around transcription end sites (TESs) and enhancement of read-through in m^6^A-containing transcripts [16]. In addition, the m^6^A modification of R-loops can be recognized by YTHDF2, leading to the elimination of RNA–DNA hybrid [13]. The m^6^A modification, as a whole, is critical for the regulation of physiological R-loops.

A group of proteins known as IGF2BPs are involved in regulating RNA processing. Recent studies indicated that IGF2BPs contain RNA recognition motifs (RRMs) and KH domains. Moreover, the KH domains are assigned to the recognition of m^6^A modification and are essential for their biological functions [17]. A similar conclusion was reached in our previous studies [18]. Although the mass spectrometry results demonstrated IGF2BP1/3 can be enriched by S9.6 IP [19, 20], the co-transcriptional roles of IGF2BPs in R-loop metabolism are not yet unknown. Almost all studies focused on the function of IGF2BPs in the cytoplasm such as RNA stabilization and translation. Here, our research revealed the new functional role of IGF2BPs as R-loop readers, which stabilize m^6^A-modified R-loops in the gene promoters. Moreover, our findings demonstrated the new crosstalk between m^6^A RNA methylation and DNA methylation during R-loop metabolism.

## Methods

### Cell lines and transfection

RWPE-1, DU-145, PC-3, 22Rv1, LNCaP, C4-2B, VCaP and HEK293T cell lines were cultured according to the ENCODE cell culture standards. IGF2BPs KO and RBM15 KO cells were generated using the CRISPR-Cas9 technology following the manufacturer’s instructions. The guided RNAs (sgRNAs) were cloned into pLentiCRSPIR V2 (Genscript, USA). Transfections were carried out with Polyplus (Franch) for plasmids and siRNAs. The sgRNA sequences used are listed in Supplementary Table S1.

### Clinical tissue samples

Thirty-six paired prostate cancer tissues and adjacent tissues were obtained from patients. The clinical tissues were all collected at the First Affiliated Hospital, School of Medicine, Zhejiang University after the approval of the Ethics Committee of Zhejiang University and informed consent. Clinicopathological characteristics of the PCa patients are listed in Supplementary Table S2.

### Antibodies, siRNAs, plasmids and RNA oligos

Antibodies used in this study were: anti-Myc tag (ab32, Abcam), anti-IGF2BP1 (ab290736, Abcam), anti-IGF2BP2 (ab128175, Abcam), anti-IGF2BP3 (ab177477, Abcam), anti-RBM15 (10587-1-AP, Proteintech), anti-DNMT1 (ab92314, Abcam), anti-DNMT3A (ab307503, Abcam), anti-DNMT3B(67,259, Cell Signaling Technology), anti-SEMA3F (SAB2107196, Sigma), anti-S9.6 (MABE1095, Sigma), anti-5-mC (ab214727, Abcam), anti-m^6^A (SAB5600251, Sigma), anti-YAP1(phosphor S127, ab76252, Abcam), anti-YAP1(66900-1-Ig, Proteintech), anti-LATS1(66569-1-Ig, Proteintech), anti-LATS2(20276-1-AP, Proteintech), anti-β-Actin(4970, Cell Signaling Technology). siRNAs against DNMT1 used were purchased from Ribobio Co.,Ltd (Guangzhou, China). pcDNA3-based vectors encoding wild-type, RRM domain mutant, KH domain mutant Myc-tagged IGF2BP1, IGF2BP2, IGF2BP3 were produced by Shanghai Yoche Biotechnology Co.,Ltd (Shanghai, China). The plasmids encoding RBM15 and SEMA3F (h-RBM15-pcDNA3.1-c-HA, M35-FLAG-SEMA3F) were obtained from Guangzhou FulenGen Co., Ltd (Guangzhou, China). DNA and RNA oligos synthesized by TsingKe Biotech Co., Ltd (Beijing, China) are listed in Supplementary Table S1.

### Lentiviruses transfection

The lentiviruses vectors, pReceiver-Lv242-FLAG, encoding IGF2BP1, IGF2BP2, IGF2BP3 and RBM15 were obtained from Guangzhou FulenGen Co., Ltd (Guangzhou, China). Lentivirus infection was performed following the manufacturer’s manuals.

### Immunoprecipitation and immunobloting

**Fig. 1:**
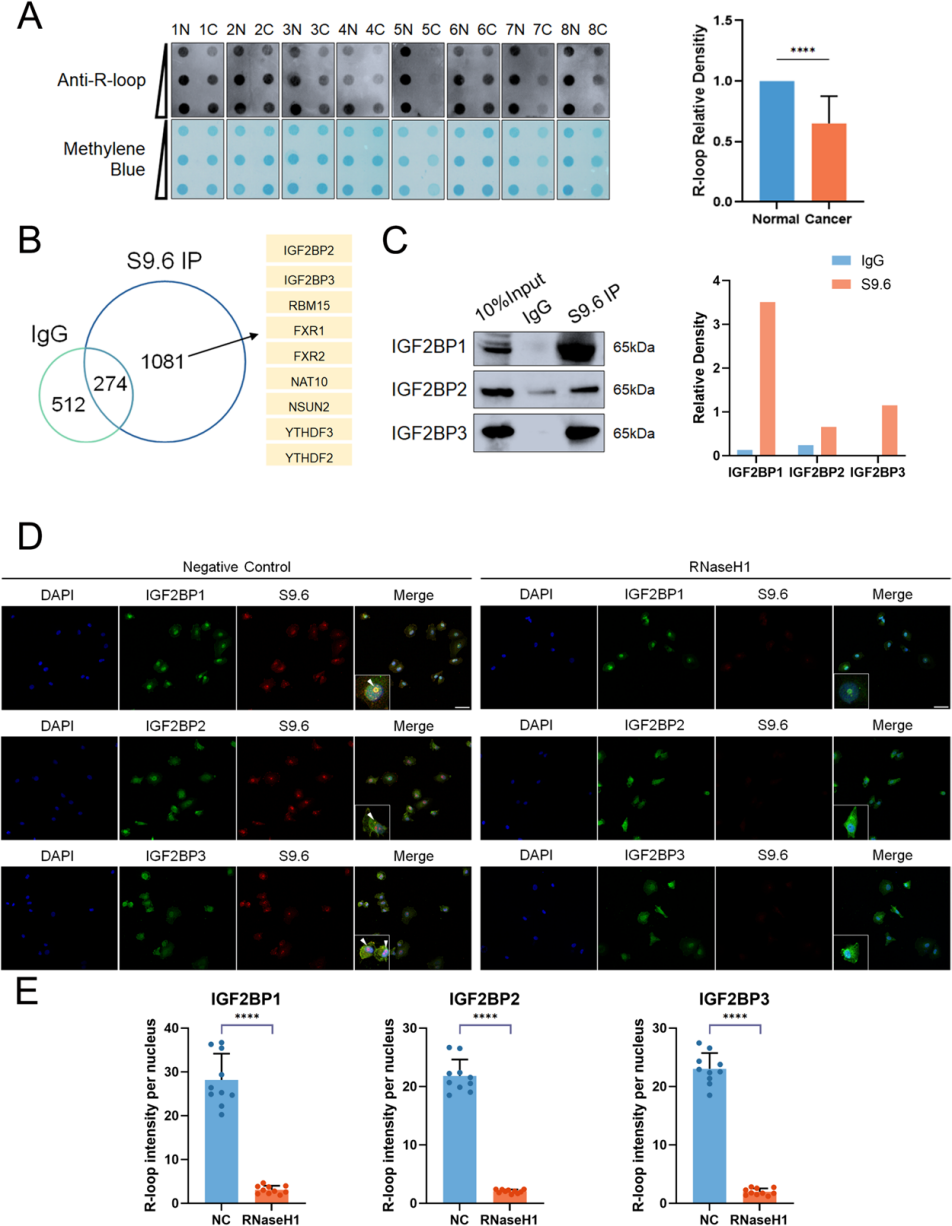
R-loops are reduced in PCa and recruit IGF2BP proteins. **A** Isolated R-loops from PCa and paired normal tissues were analyzed by Dot-blot. Methylene blue staining served as a loading control. (Left) representative dot-blot results; (Right) quantification of dot blot (normalized by the density of methylene blue staining). **B **Regulators were identified by LC-MS/MS analysis using S9.6 antibody in PC-3 cells. **C **Validation of S9.6 IP LC-MS/MS results by western blot assay. Experiments were performed with Myc-purified IGF2BP proteins and S9.6 antibody in 293T cells. (Left): Representative western blot results. (Right): Quantification of western blot assay. **D** PC-3 cells were immunostained for IGF2BP1/2/3 and S9.6; representative images are shown (scale bar: 60 μm). **E **Quantification of cellular immunofluorescence (*n* = 10 per group). Data are presented as means ± *95CI*, two-tailed unpaired t-test. *p-value < 0.05, **p-value < 0.01, ***p-value < 0.001, ****p-value < 0.0001

**Fig. 2:**
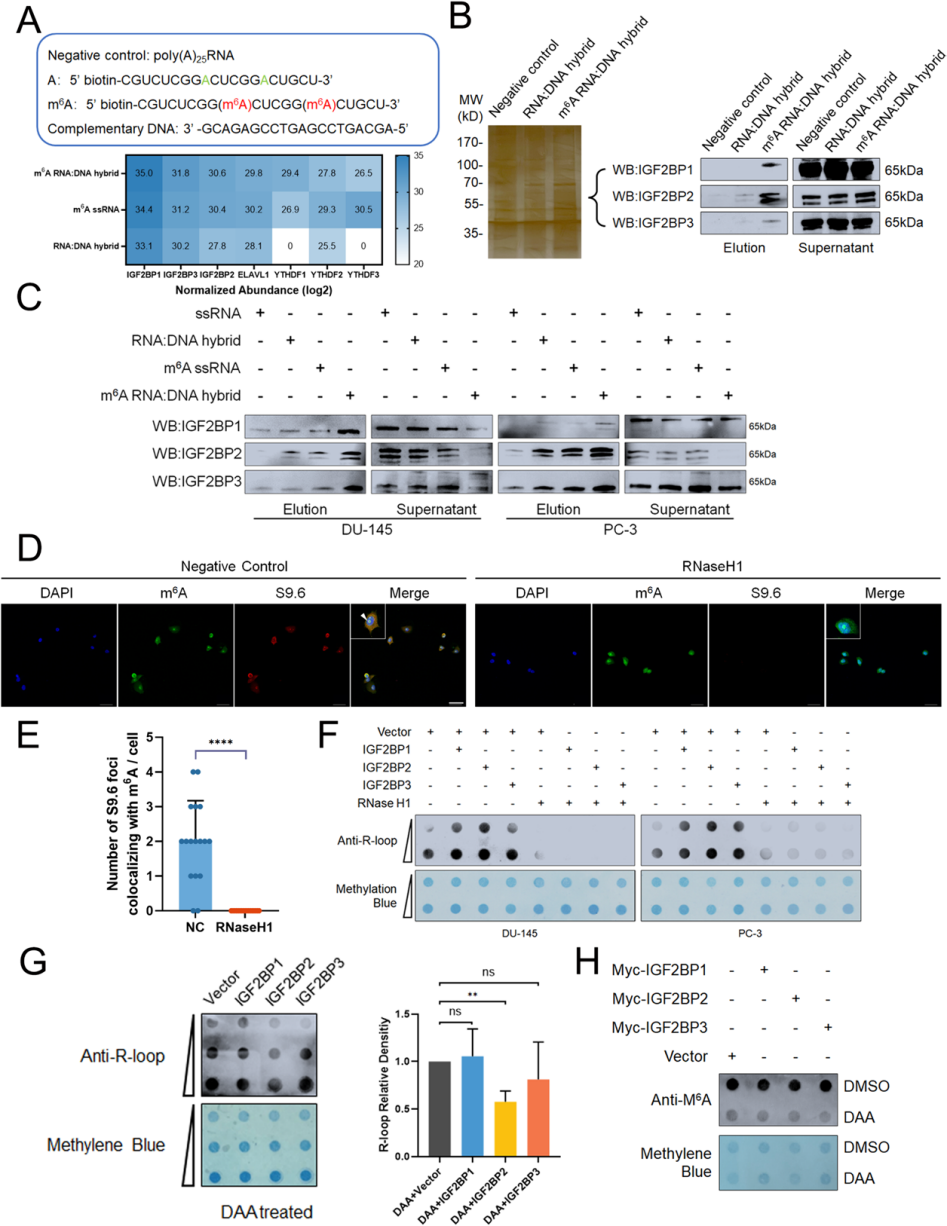
IGF2BPs preferentially bind DNA: RNA hybrids containing m^6^A-modified RNA and overexpression of IGF2BPs causes R-loop accumulation. **A **Normalized abundance of eluted pull-down proteins using RNA: DNA hybrid probes with methylated (red) or unmethylated (green) adenosine. **B **Identification of m^6^A-RNA: DNA hybrid specific binding proteins by RNA pull-down. Silver staining (upper left) and western blot (upper right) indicated pulldown of proteins from PC-3 nuclear extract. **C** Western blot results showed that IGF2BP proteins preferentially bind the RNA: DNA hybrids containing m^6^A-modified RNA. **D **Co-localization of m^6^A with R-loops in PC-3 cells. Scale bar = 60 μm. **E **Quantification of cellular immunofluorescence (*n* = 17 per group). **F **DU-145 and PC-3 cells were transfected with Myc-IGF2BP1/2/3 and vector. The R-loop levels were evaluated by dot-blot, and RNaseH1 treated samples were used as negative control. **G**-**H** PC-3 cells were treated with m6A demethylation drug (DAA, 3-Deazaadenosine) and transfected with Myc-IGF2BP1/2/3 and vector. The R-loop levels (Left of Fig. **G**) and m6A levels (**H**) are shown. (Right of Fig. **G**) Quantification of dot blot results in Fig. **G**. Data are presented as means ± SD, two-tailed unpaired t-test. *p-value < 0.05, **p-value < 0.01, ***p-value < 0.001, ****p-value < 0.0001

**Fig. 3:**
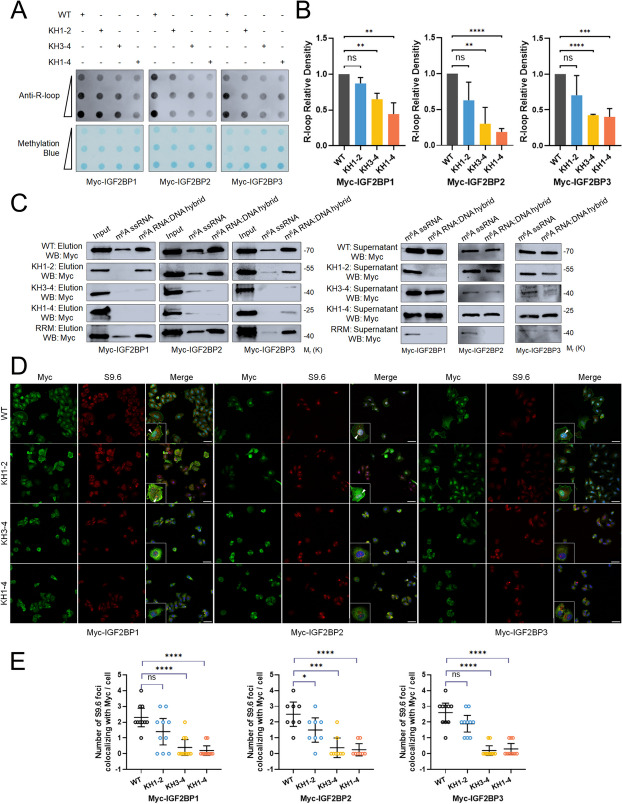
The KH domains of IGF2BPs are essential for R-loop accumulation. **A **PC-3 cells were transfected with wild-type (WT) and KH domain-mutated IGF2BPs variants. The R-loop levels were assessed by dot-blot. **B** Quantification of dot blot results in **B**. Data are presented as means ± SD. **C **RNA pull-down followed by western blot indicated in vitro binding of m6A-modified ssRNA and RNA: DNA hybrid probes with wild-type (WT), KH domain-mutated and RRM domain mutated IGF2BPs variants. **D** Co-localization of wide-type or mutated IGF2BPs variants with R-loops in IGF2BPs KO PC-3 cells. Scale bar = 60 μm.**E **Quantification of cellular immunofluorescence (Myc-IGF2BP1 group: *n* = 10;Myc-IGF2BP2 group: *n* = 8; Myc-IGF2BP3 group: *n* = 10). Data are presented as means ± *95CI*, two-tailed unpaired t-test. *p-value < 0.05, **p-value < 0.01, ***p-value < 0.001, ****p-value < 0.0001

**Fig. 4:**
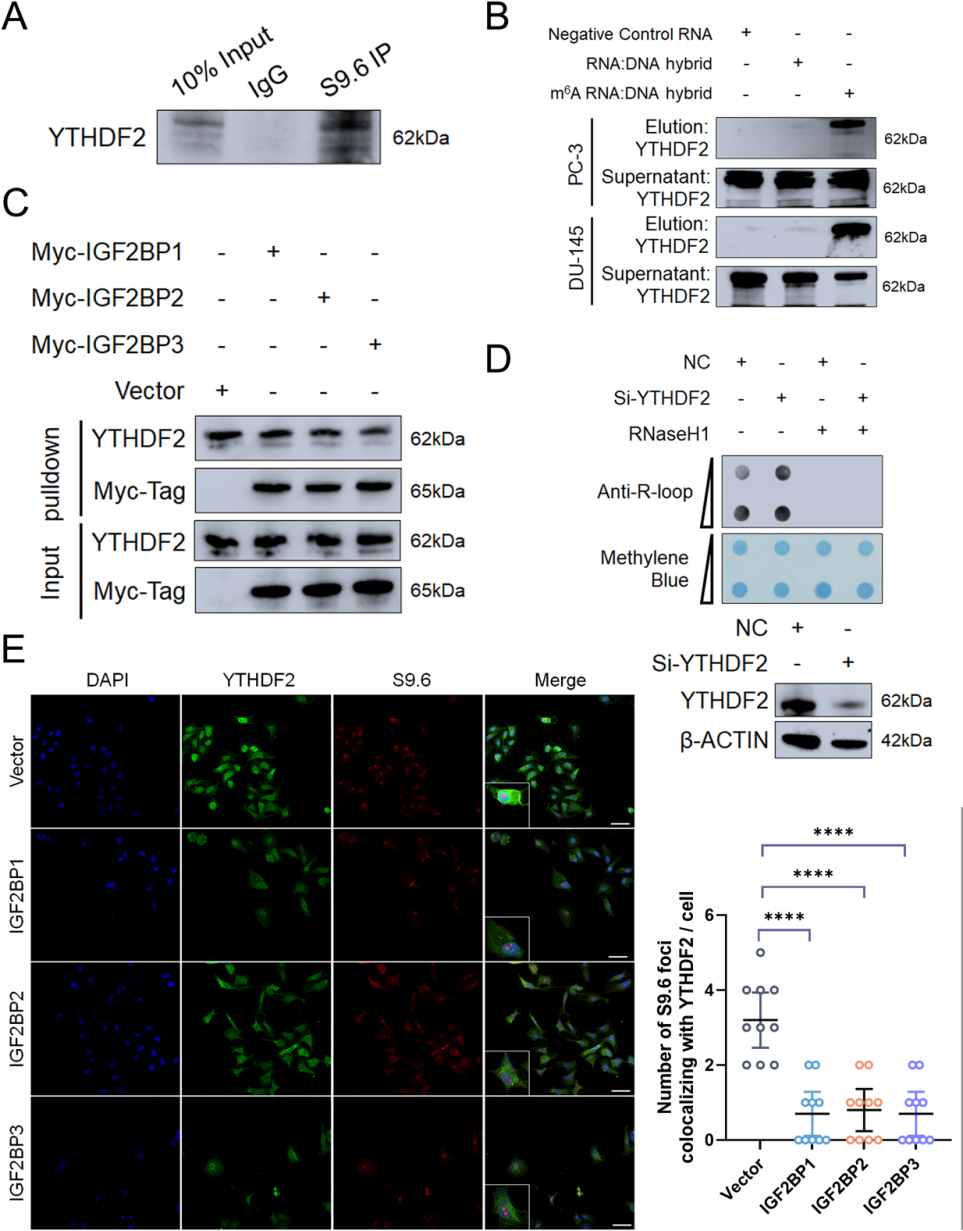
YTHDF2 interact with R-loop and YTHDF2 depletion leads to accumulation of R-loops. **A **PC-3 cell lysates were immunoprecipitated with IgG or anti-S9.6 antibody. Precipitates and input were blotted for YTHDF2. **B **Pulldown and western blot results showed that YTHDF2 preferentially bind the RNA: DNA hybrids containing m^6^A-modified RNA. **C** Pulldown followed by western blot indicated in vitro binding of m^6^A-modified RNA: DNA hybrid probe with YTHDF2 in control and IGF2BPs overexpression PC-3 cells. **D **PC-3 cells were transfected with negative control and YTHDF2 siRNA. The R-loop levels were evaluated by dot-blot, and RNaseH1 treated samples were used as control. **E **Left: Immunofluorescent staining of control and IGF2BP1/2/3 overexpression PC-3 cells. Scale bar = 60 μm. Right: Quantification of cellular immunofluorescence (*n* = 10 per group). Data are presented as means ± *95CI*, two-tailed unpaired t-test. *p-value < 0.05, **p-value < 0.01, ***p-value < 0.001, ****p-value < 0.0001

**Fig. 5:**
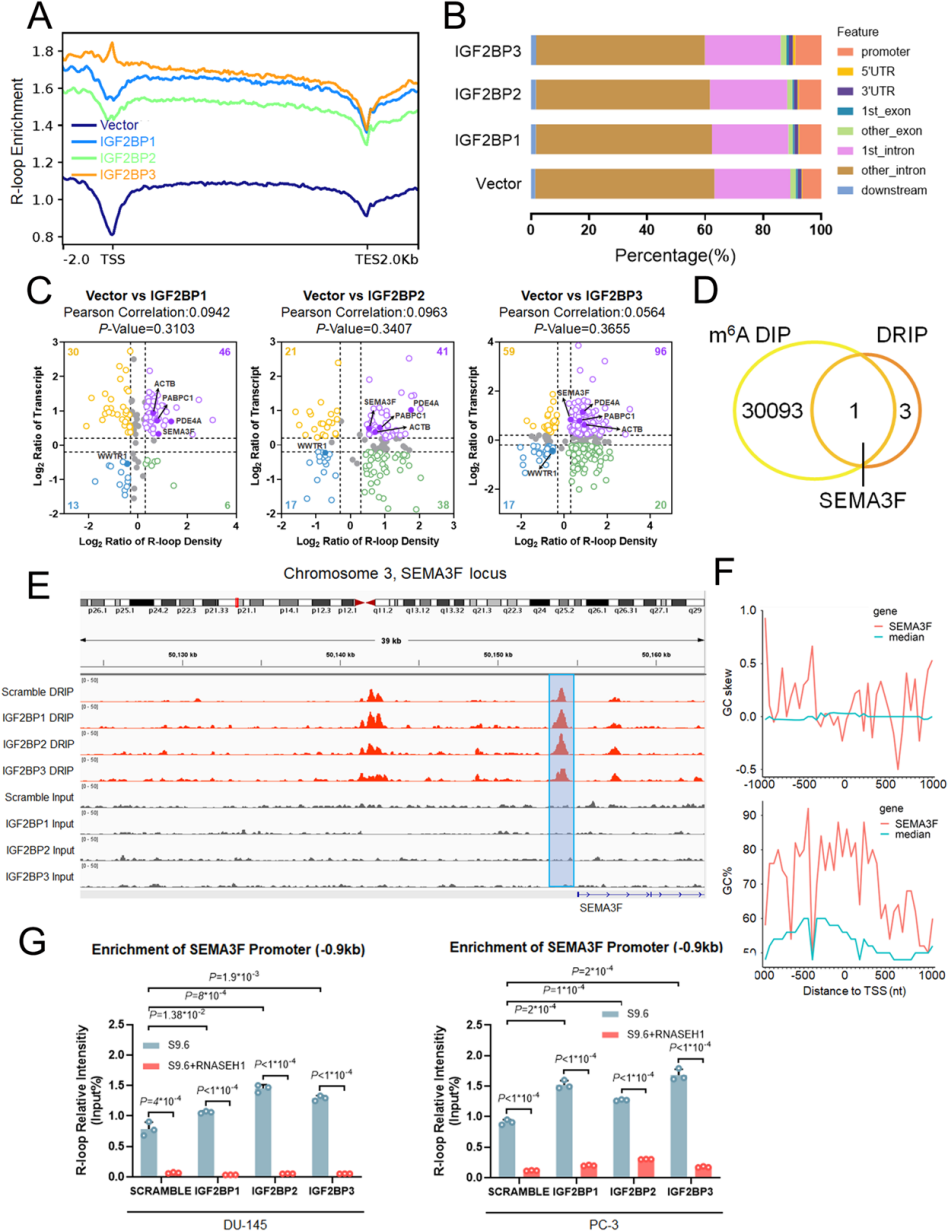
Overexpression of IGF2BPs globally upregulates target gene expression and R-loop levels in promoter regions. **A ** R-loop enrichments in randomized regions are shown as the purple (Scramble), blue (OE IGF2BP1), green (OE IGF2BP2) and orange (OE IGF2BP3) lines. **B** Distribution of the S9.6 peaks at the indicated genomic features in PC-3 cells. **C **Nine quadrant chart of mRNA expression ratios and R-loop density [Purple dots, significantly upregulate in both R-loop and mRNA; blue dots, significantly downregulate in both R-loop and mRNA; green dots, significantly downregulate in mRNA and upregulate in R-loop; green dots, significantly upregulate in mRNA and downregulate in R-loop], Correlation of mRNA expression ratios and R-loop density determined by Pearson coefficient. **D **The list of genes included in the DRIP-seq results and published m^6^A-DIP-seq results were compared using Venn diagram. **E **The coverage plots of S9.6 DRIP densities in the promoter region of the indicated gene (SEMA3F). **F** Compared to the median levels of all genes annotated in RefSeq, the SEMA3F promoter is GC rich and G-skewed. 50-bp sliding windows were used to compute GC% = [(G + C)/(G + C + A + T)]%, and GC skew = (G − C)/(G + C). The GC% and GC skew at the SEMA3F promoter (in red), and the median level for all gene promoters (in green) are shown. **G** R-loop levels of SEMA3F promoter in IGF2BPs overexpression DU-145 and PC-3 cells compared to control by DRIP-qPCR, Data are presented as means ± SD, two-tailed unpaired t-test. *p-value < 0.05, **p-value < 0.01, ***p-value < 0.001, ****p-value < 0.0001

**Fig. 6:**
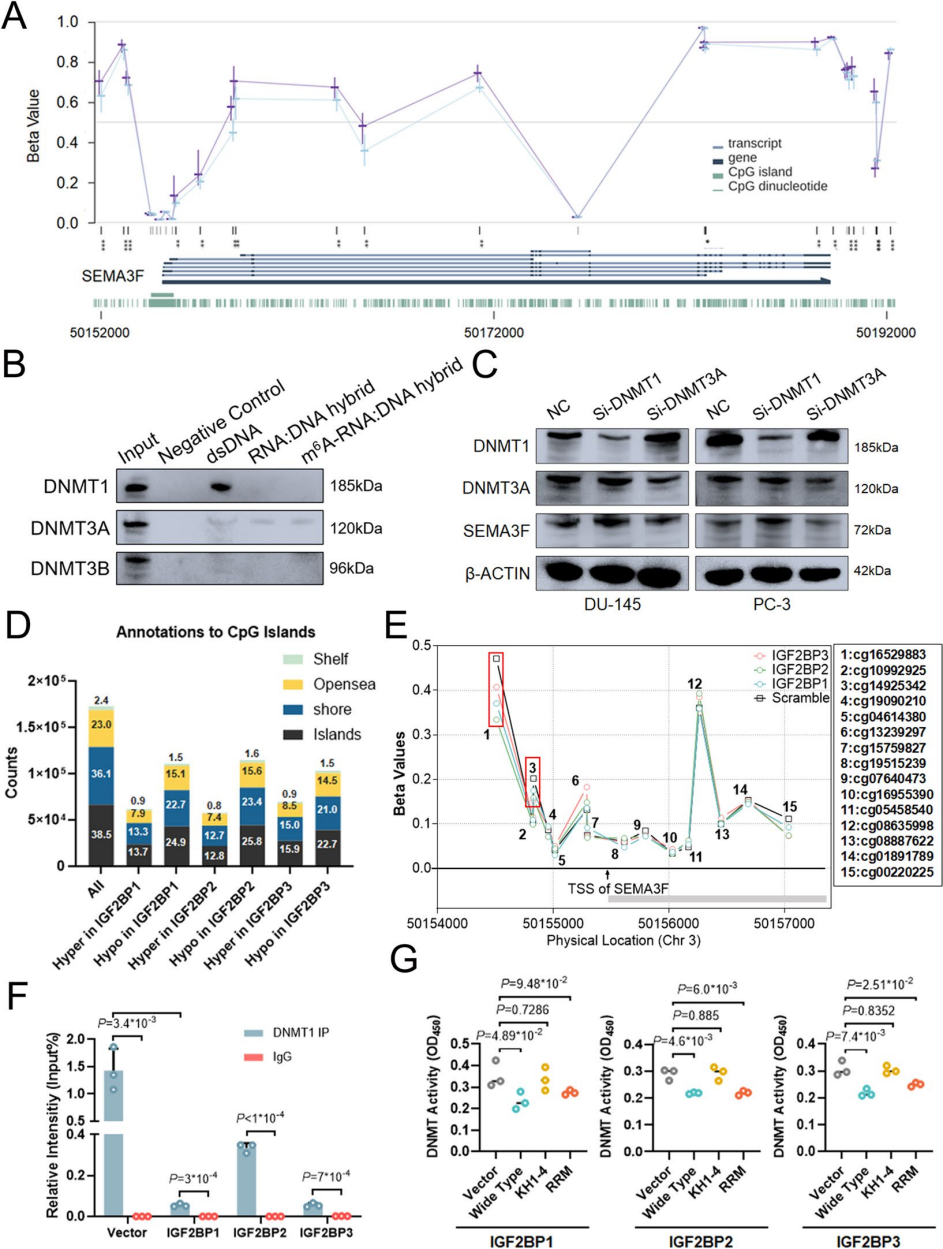
IGF2BPs upregulate SEMA3F expression via repelling DNMT1. **A **DNA methylation levels of the SEMA3F gene in TCGA prostate cancer tissues from different groups (blue lines represented the SEMA3F high expression group, purple lines represented the SEMA3F low expression group) were shown by MEPRESS. **B **Identification of dsDNA specific binding proteins by pull-down. Western blot indicated pulldown of DNMT proteins from PC-3 nuclear extract. **C **Western blot assay showed the protein levels of SEMA3F in DNMT1 and DNMT3A knockdown compared to control. **D **Overexpression of IGF2BPs altered global DNA methylation patterns which represented more hypomethylated CpG islands, CGI shelves and shores. **E** DNA methylation levels of hyper-methylated SEMA3F CpG sites (red square box) decreased in IGF2BPs overexpression teams as compared with control. **F** ChIP-qPCR analysis showed that IGF2BPs overexpression leads to more DNMT1 binding of SEMA3F promoter. **G **DNMT1 activities in wild-type and RRM mutant IGF2BPs groups were lower than control and KH1-4 mutants. Data are presented as means ± SD, two-tailed unpaired t-test

**Fig. 7:**
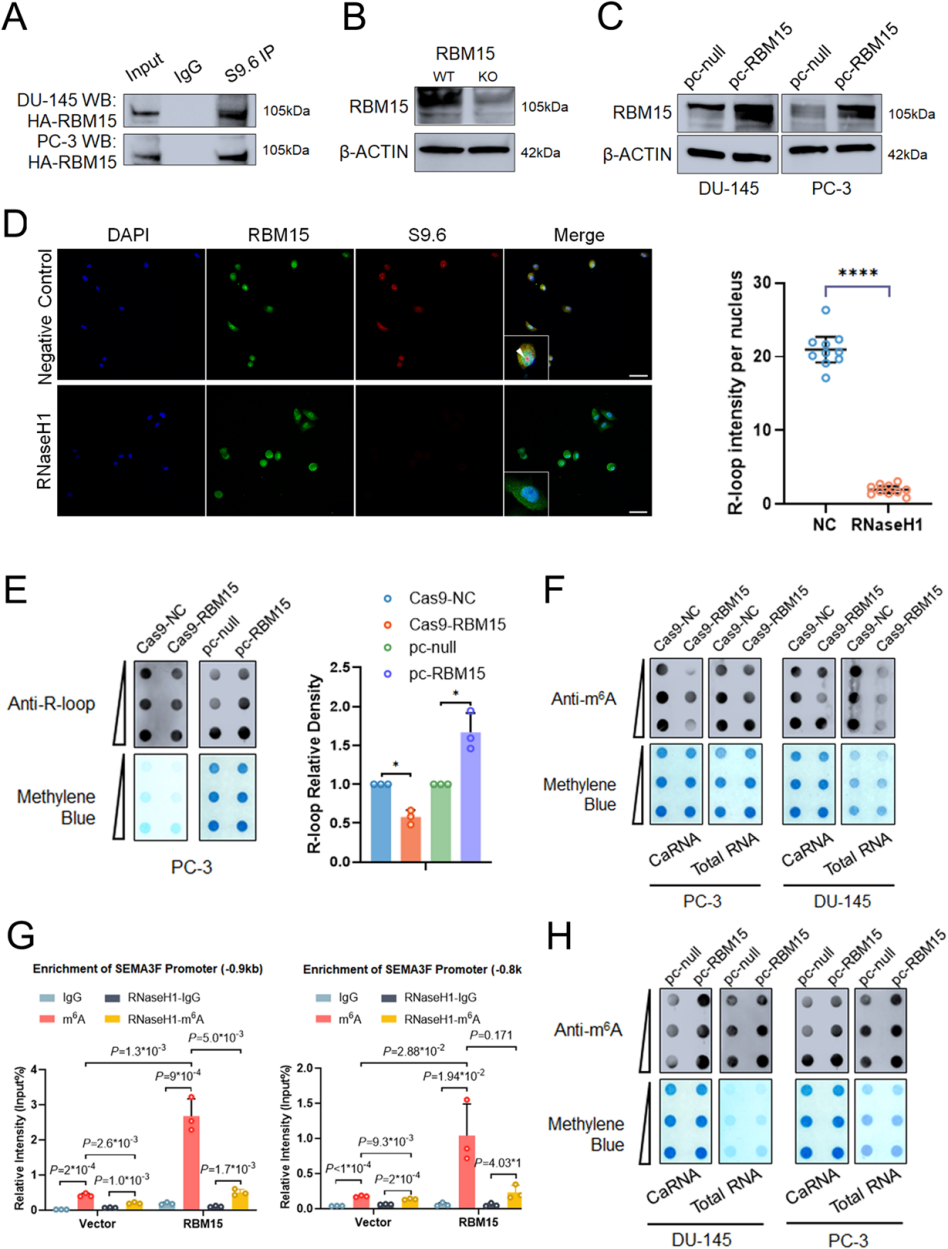
RBM15 induces m^6^A RNA methylation of R-loops. **A **Validation of S9.6 IP LC-MS/MS results by Western blot assay. Experiments were performed with HA-purified RBM15 protein and S9.6 antibody in DU-145 and PC-3 cells. **B **CRISPR-Cas9 mediated KO of RBM15 in PC-3 cells as detected by western blot. **C **PC-3 cells were immunostained for anti-RBM15 and S9.6; (Left): Representative images are shown (scale bar: 60 μm). (Right): Quantification of cellular immunofluorescence (*n* = 10 per group). Data are presented as means ± *95CI*, two-tailed unpaired t-test. **D **The R-loop levels were assessed in RBM15 overexpression cells and RBM15 KO cells compared to control. **F** RBM15 KO DU-145 and PC-3 cells were used to evaluate total RNA and chromatin-associated RNA m^6^A levels by dot-blot. **G **MeDIP-qPCR analysis indicated the m^6^A site in R-loops at SEMA3F promoter, and the m^6^A levels could be upregulated by RBM15 overexpression, Data are presented as means ± SD, two-tailed unpaired t-test. **H** DU-145 and PC-3 cells were transfected with pc-RBM15 and pcDNA 3.1 vector. The total RNA and chromatin-associated RNA m^6^A levels were evaluated by dot-blot. *p-value < 0.05, **p-value < 0.01, ***p-value < 0.001, ****p-value < 0.0001

**Fig. 8:**
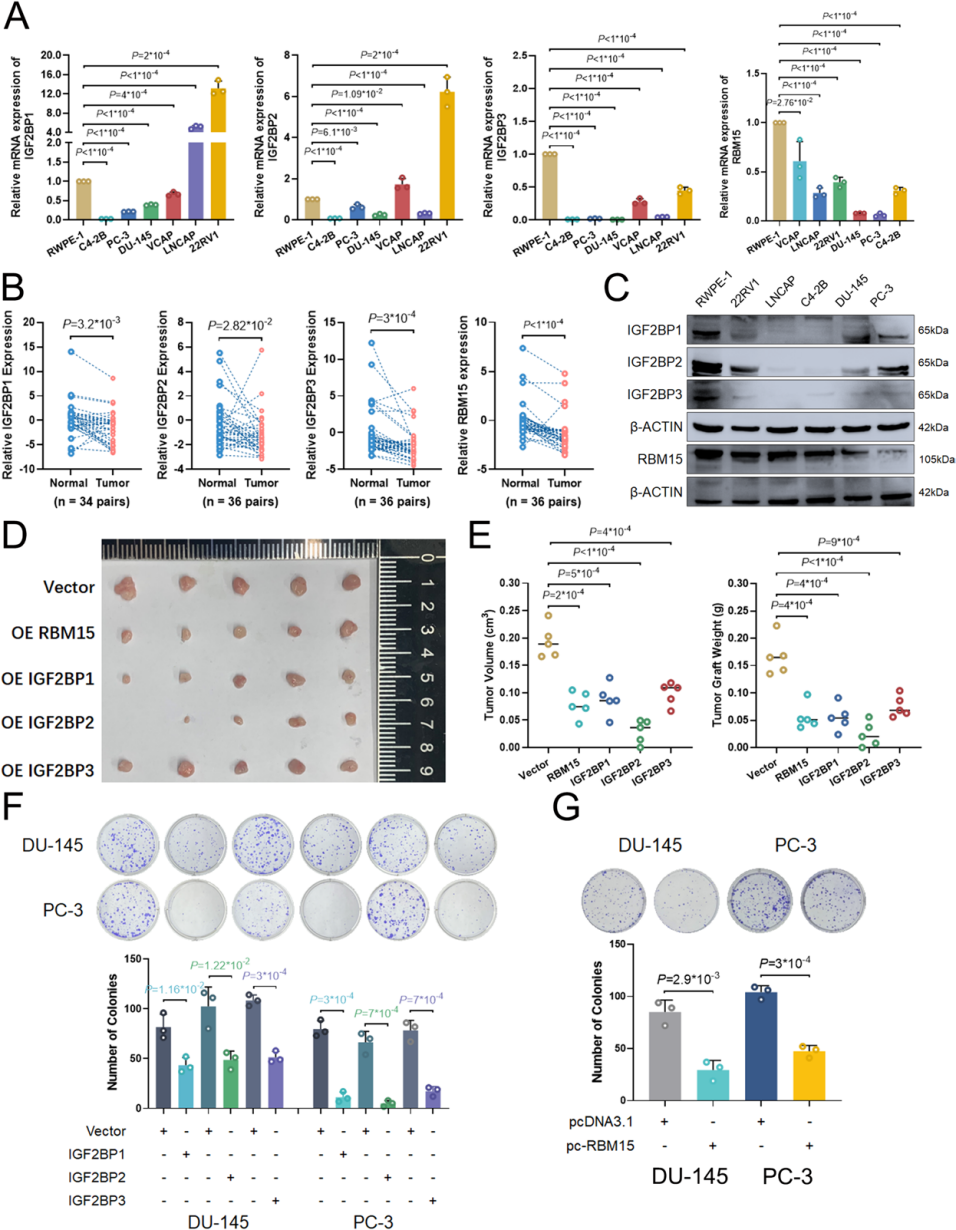
RBM15 and IGF2BPs significantly inhibited cell proliferation in prostate cancer. **A **Endogenous IGF2BPs and RBM15 mRNA levels in different human PCa cell lines. Data are presented as means ± SD, two-tailed unpaired t-test. **B** IGF2BP proteins were determined in PCa samples and paired adjacent normal tissues. Data are presented as means ± SD, two-tailed unpaired t-test. **C** IGF2BPs and RBM15 protein levels are markedly decreased in human PCa cell lines compared to RWPE-1 cell lines. (A). Endogenous IGF2BPs mRNA levels in different human PCa cell lines. **D**-**E **Typical tumor-bearing nude mice after 5 weeks (**D**), the tumor weight and volume (**E**) of 5 groups injected with same number of PC-3 tumor cells in nude mice after various treatment for 5 weeks (*n* = 5), Data are presented as means ± SD, two-tailed unpaired t-test. **D **IGF2BPs and RBM15 protein levels are markedly decreased in human PCa cell lines compared to RWPE-1 cell lines. **F**-**G **Overexpression of RBM15 and IGF2BP proteins significantly inhibited the cell proliferation in DU-145 and PC-3 cells. Data are presented as means ± SD, two-tailed unpaired t-test

**Fig. 9:**
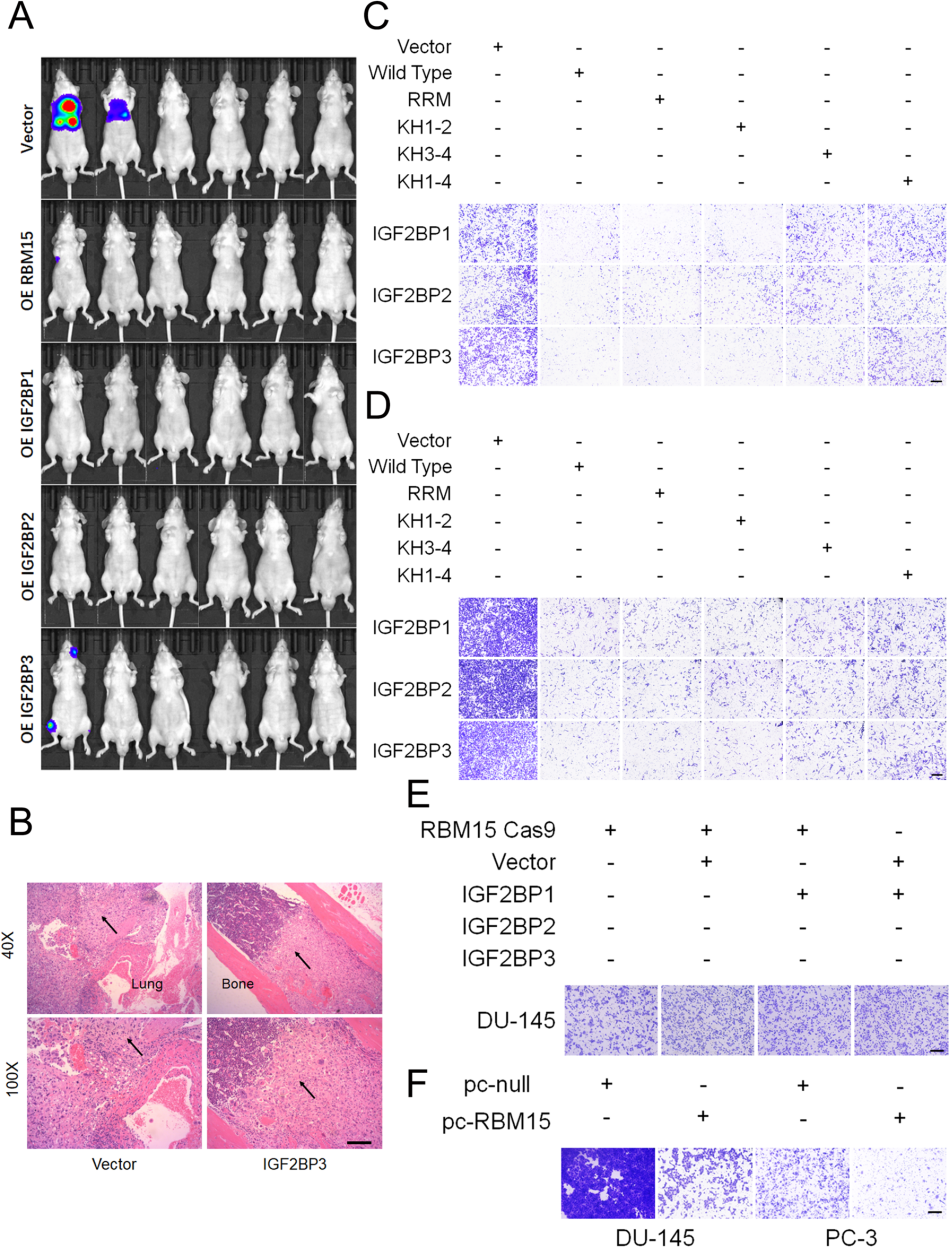
RBM15 and IGF2BPs significantly inhibited cell migration in prostate cancer. **A**-**B **Representative images of metastatic lesions at 10 weeks after the injection of indicated PC-3 cells into the tail vein of nude mice (A, *n* = 6 per group), and HE staining (**B**) of metastatic nodules of the lung and bone were shown (magnification, ×40, ×100, scale bar 100 μm). **C**-**D** Effect of wild-type or mutated IGF2BPs on restoring cell migration in IGF2BP-KO cells. Data shown represent mean value of 3 independent experiments (C: DU-145 cells, D: PC-3 cells). **E **Transwell migration assays showed the cell migration viabilities in RBM15 KO cells treated by IGF2BPs overexpression. **F **Transwell migration assays showed the inhibition of cell migration viabilities in RBM15 overexpression cells

**Fig. 10:**
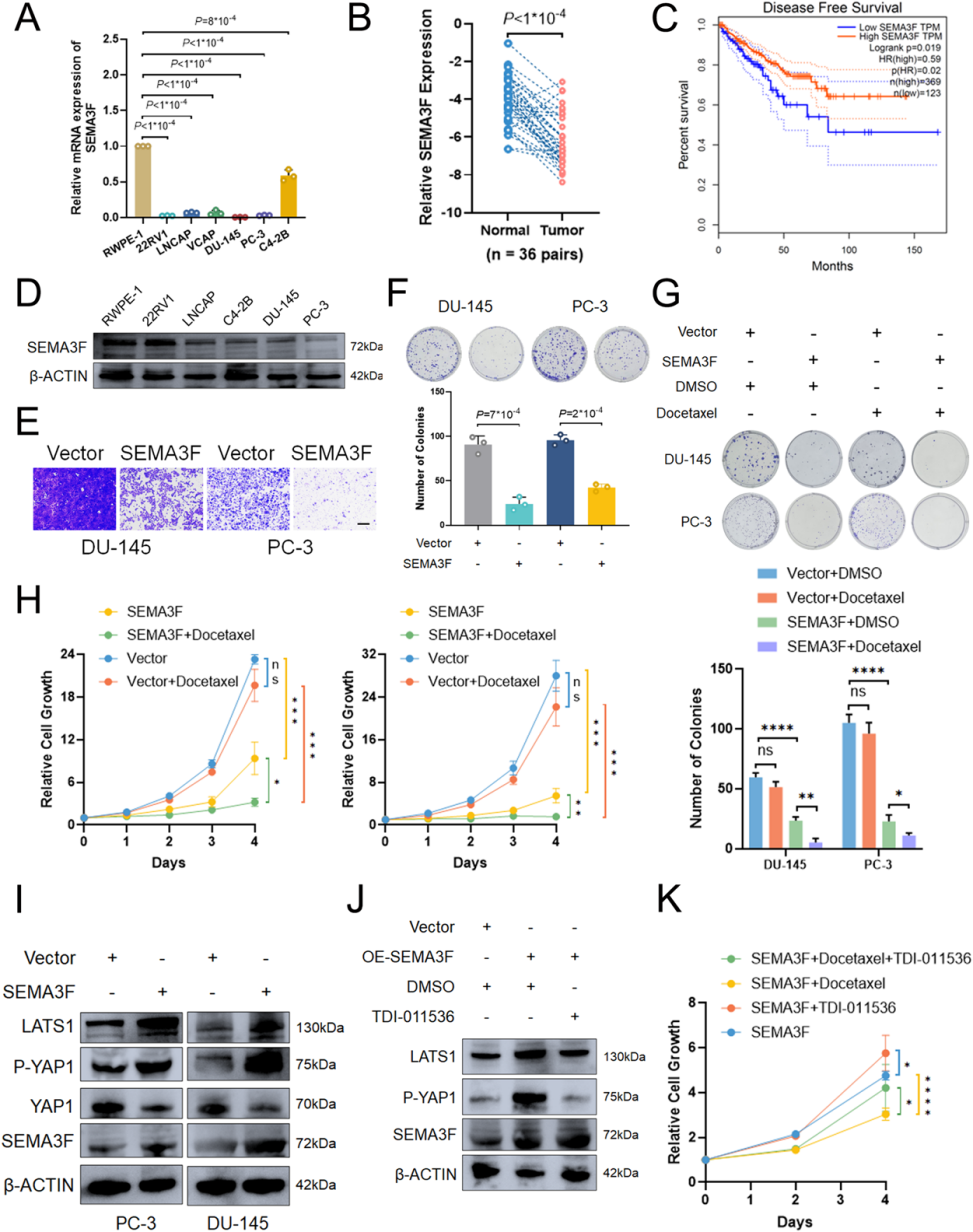
SEMA3F significantly inhibited cell proliferation, cell migration and enhanced docetaxel chemosensitivity in prostate cancer. **A **Endogenous SEMA3F mRNA levels in different human PCa cell lines. Data are presented as means ± SD, two-tailed unpaired t-test. **B **RBM15 protein was determined in PCa samples and paired adjacent normal tissues. Data are presented as means ± SD, two-tailed unpaired t-test. **C** Kaplan–Meier disease free survival (DFS) curves were performed from GEPIA portal. **D **SEMA3F protein levels are markedly decreased in human PCa cell lines compared to RWPE-1 cells. **E **Transwell migration assays showed the inhibition of cell migration viabilities in SEMA3F overexpression DU-145 and PC-3 cells. **F **Overexpression of SEMA3F significantly inhibited the cell proliferation in DU-145 and PC-3 cells, Data are presented as means ± SD, two-tailed unpaired t-test. **G **Pulldown followed by western blot indicated in vitro binding of m^6^A-modified RNA: DNA hybrid probe (using SEMA3F promoter sequence ) with YTHDF2 in control and IGF2BPs overexpression PC-3 cells. **H ** Differently transfected PC-3 cells were treated with docetaxel. Cell viability was normalized to that of the corresponding cells treated with dimethylsulphoxide (DMSO). (Upper): Representative clone formation assay. (Lower): Quantification of clone formation assay. Data are represented as means ± SD. of *n* = 3 replicates. (I). Effect of SEMA3F overexpression on Hippo pathway in DU-145 and PC-3 cells by western blot assay. **J **Effect of SEMA3F overexpression and LATS1 inhibitor(TDI-011536) on Hippo pathway in DU-145 and PC-3 cells by western blot assay. **K** Dose–response curves of differently transfected PC-3 cells treated with docetaxel, LATS1 inhibitor(TDI-011536) and SEMA3F overexpression. Cell viability was normalized to that of the corresponding cells treated with dimethylsulphoxide (DMSO). Data are represented as means ± SD. of *n* = 3 replicates.  * p -value < 0.05, ** p -value < 0.01, *** p -value < 0.001, **** p -value < 0.0001

After extracting proteins from cultured cells with a modified buffer, immunoprecipitation and following immunoblotting were performed with antibodies as described previously [21]. Briefly, 5 µg corresponding antibodies were added to PC-3 cell lysates and incubated at room temperature, followed by incubation with Protein A/G Magnetic beads (MCE) at 4 °C overnight. After washing with modified wash buffer, protein complexes were eluted by elution buffer and analyzed by immunoblotting and LC-MS/MS. All western blot experiments in Figs. 1, 2, 3, 4, 5, 6, 7, 8, 9 and 10 were repeated once; typical images from a single repeat are shown.

### Purification of IGF2BP1/2/3, METTL3 and RBM15

Full-lengths IGF2BP1/2/3 were cloned into pcDNA3.1 with Myc-tag, and full-lengths METTL3 was cloned into M35 with Flag-tag (Guangzhou FulenGen Co., Ltd). Full-lengths RBM15 was cloned into pcDNA3.1 with HA-tag (Guangzhou FulenGen Co., Ltd).The plasmid was expressed in 293T cells and incubated with DMEM medium at 37 °C. Cells were lysated in IP lysis buffer and incubated on ice. After incubation, the extract was centrifuged at 13,000 g for 20 min at 4 °C. The tag-agarose was added to supernatant and incubated at 4° C with rotation for 60 min. After centrifugation at 3000 g for 1 min, the tag-agarose-bound proteins were washed with the buffer containing 50 mM Tris pH 7.4, 150 mM NaCl. Recombinant IGF2BPs, METTL3 and RBM15 were eluted with elution buffer (50 mM Tris pH 7.4, 150 mM NaCl, 500 µg/ml peptides).

### S9.6 IP

Recombinant proteins co-immunoprecipitation with S9.6 antibody was performed from cultured PC-3 and DU-145 cells as previously described [22].

### In vitro RNA-protein pulldown assays

3’-biotin-labeled ssRNA (5’-CGUCUCGGACUCGGACUGCU-3’) and complementary ssDNA (5’-AGCAGTCCGAGTCCGAGACG-3’) were synthesized from TsingKe Biotech Co., Ltd (Beijing, China). We generated m^6^A ssRNA by substituting all two adenines with m^6^A-modified adenines. The ssRNA with or without m^6^A modification was annealed with complementary ssDNA in a 1:1 ratio. As directed by the manufacturer’s instructions, proteins in cell lysate were pulled down with the ssRNA and DNA: RNA hybrids using the Pierce magnetic RNA-Protein pull-down kit (20,164, ThermoFisher). The final elution and supernatant were analyzed by western blot and LC-MS/MS.

### LC-MS/MS

Elution samples were prepared for LC-MS/MS analysis as described previously [22]. After in-gel digestion, the peptides were dissolved on an EASY-nLC 1000 UPLC system according to the manufacturer’s protocol. And then peptides were subjected to NSI source followed by tandem mass spectrometry (MS/MS) in Q ExactiveTM Plus (Thermo) coupled online to the UPLC. The final MS/MS data were processed using Proteome Discoverer 2.4.

### Dot-blot

Total genomic DNA from prostate cells were isolated according to standard procedures. The same amount of DNA from divergent groups was diluted to the same concentration with NaOH/TE solution and denatured at 99 °C. Afterward, DNA was dot-blotted on Hybond N + membrane (GE health) via a dot-blot apparatus (Bio-rad), and linked by a UV crosslink at 1500 µJ. The membrane was stained by 0.1% methylene blue (Sigma-Aldrich). Primary antibody was diluted 1:1000 in universal antibody diluent (NCM Biotech). After incubation at 4 °C, the membrane was washed twice gently in 0.02% TBST for 10 min. Secondary antibody was diluted 1:5000 in 5% BSA in 0.02% TBST and then washed three times. All dot blot experiments in Figs. 1, 2, 3, 4, 5, 6, 7, 8, 9 and 10 were repeated once; typical images from a single repeat are shown.

### Immunofluorescence

Cells for immunofluorescence were fixed in ice-cold methanol for 15 min at -20 °C and further treated with 0.1% Triton X-100 for 30 min. Cells were then blocked by 5% BSA for 30 min at room temperature. Primary antibodies were diluted in universal antibody diluent (NCM Biotech) and incubated with prostate cells overnight at 4 °C. The cells were then washed three times with 0.02% TBST, and were incubated with secondary antibodies for 50 min at room temperature followed by wash with 0.02% TBST twice. DAPI was incubated with cells at room temperature for 5 min. Images were acquired with a STEDYCON confocal microscope and processed using Image J and Adobe Photoshop. Quantification of the m^6^A, RBM15, IGF2BP1/2/3 and S9.6 signal intensities was performed according to the previously described method [7].

### DRIP-seq and m^6^A DIP

Genomic DNA was isolated from prostate cells by Proteinase K and RNase A treatment in lysis buffer, followed by phenol-chloroform extraction and ethanol precipitation. The DNA was fragmented to 200–500 bp by sonication. 20 µg of genomic DNA was used for each immunoprecipitation. S9.6 DRIP was performed as described previously using S9.6 antibody [23]. The DNA libraries were sequenced on the Illumina sequencing platform by Genedenovo Biotechnology Co., Ltd (Guangzhou, China). M^6^A DIP was carried out as described in a previously published study [13] using anti-m^6^A antibody. With respect to the data analysis, sequencing reads were aligned to genome reference sequences using HISAT2 software (v2.1.0). The DRIP enriched regions (peaks) were visualized by Integrative Genomics Viewer (IGV).

### RNA-seq

Total RNA was isolated from different groups of PC-3 cells by standard protocol. RNA-seq libraries were constructed using standard Illumina RNA-seq protocols by Genedenovo Biotechnology Co., Ltd (Guangzhou, China). The paired-end clean reads were mapped to the reference genome using HISAT2. 2.4. Principal component analysis (PCA) was performed with R package gmodels (http://www.r-project.org/) in this experience. RNAs differential expression analysis was performed by DESeq2 software between two different groups (and by edgeR between two samples). The genes/transcripts with the parameter of false discovery rate (FDR) below 0.05 and absolute fold change ≥ 2 were considered differentially expressed genes/transcripts.

### ChIP

DU-145 and PC-3 cells cultured in 10 cm dishes were fixed with 1% paraformaldehyde for 15 min at room temperature. The ChIP assay was carried out using the ChIP kit (Merck and Millipore). Briefly, cells were lysed using lysis buffer on ice. Nuclei was collected and fragmented by ultrasound. The chromatin was isolated and added to DNMT1 beads in ChIP buffer. After incubation overnight, the beads were washed and eluted. The eluted chromatin was combined and crosslinks reversed, followed by DNA purification by PCR purification columns (Thermo Fisher). The eluted ChIP-DNA was used for RT-PCR analysis.

For ChIP-re-ChIP, the chromatin was isolated and added to RBM15 beads in ChIP buffer. Immunoprecipitates were then eluted with 30 µL 10mM DTT at 37 °C for 30 min. Then they were diluted 20x with Re-ChIP buffer (1% Triton X-100, 150 Mm NaCl, 2 mM EDTA, 20 mM Tris-HCl, and 1x cocktail) on ice. Next, they were incubated with anti-m6A antibody at 4 °C overnight with mixing. Washes and IP elution were performed according to ChIP kit protocols, followed by DNA purification by PCR purification columns (Thermo Fisher). The eluted ChIP-DNA was used for RT-PCR analysis. All the primers used are shown in Supplementary Table S1.

### DNMT1 activity assay

The DNMT1 activity assay (Abcam) was used to quantify DNMT1 binding activity. The procedures to measure the DNMT1 activity were directed by manufacturer’s manual.

### MethylationEPIC (850 K) BeadChip

The MethylationEPIC BeadChip experiments and data analysis of the PCa samples were conducted by OE Biotechnology Co.Ltd. (Shanghai, China). DNA concentration and integrity were assessed by a NanoDrop 2000 spectrophotometer (Thermo Fisher Scientific, Waltham, MA, USA) and agarose gel electrophoresis, respectively. DNA was bisulfite treated using the Zymo Research EZ DNA methylaiton-Glod Kits (Zymo Research, Irvine, CA, USA). Bisulfite-converted DNA was analysed on an Illumina Infinium MethylationEPIC(850 K) BeadChip (Illumina). Finally, Illumina iSCAN was used to scan the chip to get the Idat files. Idat files were imported and then preprocessed with ChAMP(version 2.12.4) package in R to get raw data. Next, the raw data was normalized with BMIQ method. Statistical differences in continuing variables between two groups were compared by t-test. The significantly DMS(differential methylation Sites) were identified by a threshold of |deltaBeta| > 0.1 and *P*.Value < 0.05.

### Cell migration and proliferation assays

All specific procedures were performed as previously described [18].

### Animal models and in vivo imaging

Subcutaneous transplantation models were prepared as described previously [24]. All the animal studies and protocols followed the institutional guidelines of the First Affiliated Hospital, School of Medicine, Zhejiang University.

### RNA isolation and quantitative PCR

Total RNA was extracted from prostate cell lines and PCa tissues by RNAiso plus (Takara, Japan) and analyzed by RT-qPCR as previously described [25]. All the primers used in the study are shown in Supplementary Table S1.

### Databases used

Several user-friendly databases were utilized to download data, analyze or refer to in this study. TCGA database (https://portal.gdc.cancer.gov), starBase online database (https://rnasysu.com/encori/), GEPIA online database (http://gepia2021.cancer-pku.cn/), LinkedOmics online database (http://www.linkedomics.org/), SRAMP (http://www.cuilab.cn/sramp), and Venn diagram (http://bioinfogp.cnb.csic.es/tools/venny/index.html).

#### Statistics

Data are presented as mean ± SD. Differences between two groups were evaluated using the two-tailed, unpaired t test. Survival curves were constructed using the Kaplan-Meier method and analyzed by the log-rank test. All statistical analysis was performed using the GraphPad Prism 9.0 software. Statistical significance was defined as *P* value of < 0.05.

## Results

### R-loops are reduced in PCa and recruit IGF2BP proteins

We employed the well-established monoclonal antibody S9.6 to analyze R-loops in PCa tissues and cell lines to see if overall R-loop levels are altered in prostate cancer. The findings demonstrated that R-loop abundance was higher in surrounding tissues than in carcinomas (Fig. 1A). R-loop levels in prostate cancer cell lines were also compared to RWPE-1 cells (Fig S1A). S9.6 immunoprecipitation was performed to investigate how R-loops are dynamically regulated in PC-3 cells. And the pull-down elution fractions were examined by mass spectrometry. S9.6 IP elution proteins are engaged in various RNA processing processes, including mRNA splicing, degradation, and stabilization, which is consistent with previous findings (Fig. 1B, S1B). IGF2BP family are known m^6^A readers which play a crucial role in controlling the fate of methylated mRNA. Furthermore, m^6^A is critical for R-loop resolution in recent studies [13, 15]. Therefore, we investigated the role of IGF2BP family in R-loop resolution. We first confirmed that endogenous and purified recombinant IGF2BPs physically binds R-loops using S9.6 IP assays (Fig. 1C, S1C).

Given the finding that IGF2BPs binds R-loop, we examined their colocalization via immunofluorescence assays. In PC-3 cells, IGF2BP1/2/3 spontaneously formed foci that overlapped with R-loop foci (Fig. 1D and E). Mutual co-immunoprecipitation(Co-IP) experiments were performed to assess that IGF2BP1/2/3 bind with other IGF2BP proteins in vitro (Fig.S1D, E). To further determine the domains involved in the IGF2BPs interactions, serial deletion mutants of IGF2BPs were constructed (Fig.S2A). Co-IP with the deletion mutants of IGF2BPs indicated that KH domains were crucial for the binding of IGF2BP proteins (Fig.S2B, C, D). Taken together, these data indicated that IGF2BP proteins may be one group of crucial R-loop regulators.

### IGF2BPs preferentially bind DNA: RNA hybrids containing m^6^A-modified RNA and overexpression of IGF2BPs causes R-loop accumulation

Since IGF2BPs are known m^6^A readers in mammalian cells [21], we anticipated that IGF2BPs could bind m^6^A-modified DNA: RNA hybrids. We initially employed negative control RNA, biotin-labeled DNA: RNA hybrids with or without two m^6^A sites for pull-down (Fig. 2A). Then, methylated single-stranded RNA bait (ss-m^6^A) and DNA: RNA hybrids with or without m^6^A were used for RNA pull-down, followed by mass spectrometry analysis. All three IGF2BP proteins were identified by MS and confirmed to selectively bind the methylated DNA: RNA hybrids with a 1.96–5.80 fold higher affinity than the unmethylated probes and 0.14–0.53 fold higher than methylated single-stranded RNA bait (Fig. 2A). The 20-bp DNA: RNA hybrids containing m^6^As captured IGF2BPs in cell lysates more efficiently than negative control RNA and DNA: RNA hybrids without m^6^A modification (Fig. 2B, S3A). Thus, IGF2BP proteins preferentially bind the DNA: RNA hybrids containing m^6^A-modified RNA (Fig. 2C, S3B). In addition, subsequent immunofluorescence staining showed the enrichment of m^6^A signal in R-loops (Fig. 2D and E). Given that individual IGF2BPs bind with each other IGF2BP proteins and R-loops, we hypothesized that IGF2BPs would influence R-loop levels. The overall R-loop levels markedly increased in IGF2BPs OE cells (Fig. 2F, S3C), which can be impaired by m^6^A demethylation (Fig. 2G and H, S3D). In order to prevent the potential consequences of endogenous IGF2BP proteins, PC-3 cells with IGF2BPs CRISPR KO were generated (Fig.S3E). Interestingly, the R-loop accumulation induced by IGF2BPs overexpression was not visibly affected by another IGF2BPs knockout (Fig.S4A). Taken together, overexpression of IGF2BPs causes R-loop accumulation in prostate cancer cells in an m^6^A-dependent way.

### The KH domains of IGF2BPs are essential for R-loop accumulation

All of the IGF2BP proteins were structurally composed of similar functional domains, including four K homology (KH) domains and two RNA recognition motif domains (RRM). Studies indicated that KH domains are essential for the binding of IGF2BPs to m^6^A-modified mRNAs [17]. The RRM domains and KH domains of the IGF2BP mutants were truncated as previously described [9]. KH3-4 and KH1-4 alterations almost impaired the upregulation of R-loop levels (Fig. 3A and B). Similarly, Only truncating the KH1-2 domains marginally inhibited the association of IGF2BPs with ss-m^6^A probes and m^6^A-RNA: DNA hybrid probes, whereas the KH3-4 and KH1-4 alterations virtually removed the connection (Fig. 3C, S4B). Notably, KH3-4 and KH1-4 mutations also affected the colocalization of IGF2BPs with R-loops in the nucleus (Fig. 3D and E). Collectively, these findings demonstrated the crucial roles of KH domains in IGF2BPs binding of m^6^A-modified DNA: RNA hybrids.

### YTHDF2 preferentially bind DNA: RNA hybrids containing m^6^A-modified RNA and knockdown of YTHDF2 causes R-loop accumulation

We next asked what is the specific mechanism underlying IGF2BPs recruitment of the R-loops. Previous studies revealed the opposite role of IGF2BPs versus YTHDF2 on mRNA stabilization [17]. And YTHDF2 was identified as one of crucial R-loop regulator in an m^6^A-dependent way previously [13]. We reasoned that a similar mechanism might be at R-loop metabolism in prostate cancer cells as well. We first confirmed that YTDHF2 binds R-loops using S9.6 IP assays in PC-3 cells (Fig. 4A, S5A). The DNA: RNA hybrids containing m^6^As captured YTHDF2 in cell lysates more efficiently than negative control RNA and DNA: RNA hybrids without m^6^A modification (Fig. 4B, S5B). Knockdown of YTHDF2 reduced the overall R-loop levels in PC-3 cells (Fig. 4D). It is therefore likely that YTHDF2 fulfils a similar functional role to R-loop metabolism in PC-3 cells. Interestingly, our studies indicated that the binding of DNA: RNA hybrids containing m^6^As and YTHDF2 can be impaired by IGF2BPs overexpression (Fig. 4C, S5C). Notably, IGF2BPs overexpression also affected the colocalization of YTHDF2 with R-loops in the nucleus (Fig. 4E). Taken together, IGF2BPs may regulate the R-loop metabolism via precluding the binding of YTHDF2 with R-loops.

### Overexpression of IGF2BPs globally upregulates target gene expression and R-loop levels in promoter regions

Following that, we performed RNA-seq on individual IGF2BPs overexpression and control PC-3 cells. The similarity between each IGF2BPs overexpression sample was shown using principal component analysis (PCA) (Fig.S6A). Further DRIP-seq investigation revealed that R-loops accumulate when IGF2BPs were overexpressed (Fig. 5A). According to the combined data, IGF2BPs overexpression groups show higher levels of R-loop signals throughout promoter regions than the control group (Fig. 5B). Individual published IGF2BPs CLIP-seq(starBase, https://rnasysu.com/encori/) targets overlapped with 57.2%, 70.3%, and 71.2% of them, respectively (Fig.S6B). In general, the levels of most transcripts were considerably increased in each IGF2BPs overexpression team (88.5%, 51.9%, and 82.8%), whereas R-loop signals accumulated in the promoter regions (Fig. 5C). Combined analysis suggested no sigificant correlation between R-loop density and transcript levels (*r* = 0.0942, 0.0963, 0.0564; P-value = 0.3103, 0.3407, 0.3655). Five putative co-targets were shared by the three IGF2BP proteins (Fig. 5C). Among them, only SEMA3F contains one m^6^A peak in the R-loop region, as discovered by m^6^A-DIP in one published study(Fig. 5D) [13]. Furthermore, we found the promoter region of SEMA3F is significant GC rich and revealed high GC skewing (Fig. 5F). Previous research has consistently shown that a G-rich non-templated DNA strand favors R-loop formation.

We then investigated whether IGF2BPs had a direct effect on R-loop metabolism in the SEMA3F locus of PC-3 cells. Notably, overexpression of IGF2BPs increased R-loop formation at the SEMA3F promoter. Furthermore, subsequent investigations of the SEMA3F revealed a high positional overlap of SEMA3F in DRIP-seq assays (Fig. 5E). The R-loop level and expression of SEMA3F was confirmed by qPCR to be markedly upregulated upon IGF2BPs overexpression (Fig. 5G, S6D, S7A). Similar findings were obtained in the western blot experiments, which can be partially rescued by overexpression of RNaseH1 (Fig.S7C). All IGF2BPs knockouts decreased SEMA3F expression in PC-3 cells (Fig.S6E). Then we interrogated TCGA database to determine the correlation between the mRNA expression of SEMA3F and IGF2BPs. And findings revealed a significant positive correlation between IGF2BP2/3 and SEMA3F in PCa tissues (Fig.S7D). The correlated regulation of R-loop levels and gene expression via IGF2BP proteins revealed that IGF2BPs are responsible for the R-loops and expression of target genes.

## IGF2BPs upregulate SEMA3F expression via repelling DNMT1 and YTHDF2

Specifically, we investigated how gene expression is affected by R-loops. Recent study has shown that R-loop abundance is connected with de novo methylation and is responsible for DNA methylation prevention [6]. Nevertheless, it is unknown whether R-loops induced by IGF2BPs alter DNA methylation patterns in prostate cancer. Consequently, we questioned if R-loops may influence target promoter DNA methylation in PCa. Initially, we compared promoter methylation status in SEMA3F high expression and low expression PCa patients. We discovered considerably higher methylation levels in SEMA3F promoter areas of low expression team compared to high expression as found by MEXPRESS database (Fig. 6A). We further analyzed the correlation between SEMA3F expression and expression of major DNA methyltransferases (DNMTs). SEMA3F mRNA abundance was all negatively linked with DNMTs, as expected (Fig.S8E). To further investigate the relationship between DNMTs and SEMA3F, we initially employed biotin-labeled ssDNA, DNA: RNA hybrids with or without one m^6^A sites using SEMA3F promoter sequence for pulldown (Fig.S7B). The ssDNA captured DNMT1 in cell lysates much more efficiently than DNA: RNA hybrids with or without m^6^A modification (Fig. 6B, S5F). However, all the probes failed to enrich DNMT3A and DNMT3B (Fig. 6B, S5F). Silencing DNMT1 also resulted in upregulated SEMA3F expression while DNMT3A knockdown almost did not affect SEMA3F protein levels (Fig. 6C, S2H). After that, chromatin immunoprecipitation indicated that DNMT1 binds directly to the promoter of SEMA3F, which is severely suppressed by IGF2BPs overexpression (Fig. 6F).

Previous research has documented that R-loop has substantially lower DNMT1 activity than ssDNA [16]. IGF2BPs consistently inhibited DNMT1 activity, which can be restored by KH1-4 mutants (Fig. 6G, S8C). With that confirmation, we compared SEMA3F promoter methylation in IGF2BPs overexpression PC-3 cells to control, and discovered that IGF2BPs overexpression reduced methylation levels at the SEMA3F promoter (Fig. 6E).

Moreover, overexpression of IGF2BPs altered global DNA methylation patterns, which were accompanied by hypomethylation of CpG islands (CGIs), CGI shelves and shores (Fig. 6D, S8D). Importantly, IGF2BPs overexpression resulted in a significant decrease in global DNA methylation by dot-blot, which could be partially restored by KH3-4 and KH1-4 mutants (Fig.S8A, B). These findings suggested that IGF2BPs facilitated target transcription by avoiding DNA methylation.

As mentioned above, IGF2BPs regulate R-loop metabolism by precluding the binging of YTHDF2 with R-loops. The binding of DNA: RNA hybrids(using SEMA3F promoter sequence) containing m^6^A and YTHDF2 can be impaired by IGF2BPs overexpression (Fig.S11E). Moreover, ChIP-PCR assays revealed that IGF2BPs overexpression downregulated the binding of YTHDF2 with SEMA3F promoter (Fig.S11F). Meanwhile, silencing of YTHDF2 upregulated the expression of SEMA3F(Fig.S11J). Taken together, these results demonstrated that IGF2BPs upregulate SEMA3F expression via precluding DNMT1 and YTHDF2.

### RBM15 induces m^6^A RNA methylation of R-loops

METTL3 is crucial for the m^6^A methylation of co-transcriptional R-loops [16]. Nevertheless, S9.6 IP-LC/MS-MS analyses in PC-3 cells discovered approximately 1300 proteins involved in R-loop metabolism except METTL3. As a result, we investigated if other enzymes were involved in m^6^A methylation of R-loops in PCa cells. We searched that the location of RBM15/15B proteins is believed to influence m^6^A modification sites according to the previous studies, and RBM15 was included in the proteins identified by LC-MS/MS in Fig. 1B. Since RBM15 binds METTL3 (Fig.S9F), we investigated its activity at R-loops and its function in m^6^A-modified R-loops. We initially performed co-immunoprecipitation with the S9.6 antibody in PC-3 cells, and the results revealed the interactions between R-loops and purified recombinant RBM15 (Fig. 7A). Then we observed that endogenous RBM15 was colocalized with R-loops and m^6^A modification (Fig. 7D, S9A). Next we assessed whether RBM15 was involved in m^6^A modification of R-loops by dot-blot. RBM15 Knockout significantly reduced R-loop levels and m^6^A modification (Fig. 7B, E and F, S9C, S11A), whereas RBM15 overexpression markedly upregulated R-loop levels and m^6^A modification (Fig. 7C, E and H, S9D). As expect, overexpression of RBM15 upregulated the binding of METTL3 and R-loops (Fig. S10B). Silencing METTL3 downregulated the overall R-loop levels in PCa cells (Fig.S10D).

SEMA3F was further studied to corroborate the regulation in m^6^A modification of R-loops. SRAMP was used to predict the detail m^6^A sites in SEMA3F promoter region (Fig.S9E). When RBM15 expression was upregulated, the methylation level of the m^6^A site at SEMA3F promoter altered considerably via MeDIP-PCR and re-ChIP-PCR (Fig. 7G, S11B). Moreover, the expression of RBM15 mRNA markedly positively correlated with SEMA3F across PCa samples (Fig.S10E). Consistently, SEMA3F expression was upregulated when RBM15 was overexpression (Fig.S12I). After that, we asked whether IGF2BPs promote R-loops formation and targets transcription in an m^6^A-dependent manner. We measured R-loop levels in IGF2BPs overexpression and control groups after RBM15 knockout-induced m^6^A RNA demethylation, and no significant difference was found between these groups (Fig.S9B). Notably, similar results were observed in western blot assay to assess the expression of SEMA3F in RBM15 KO cells (Fig.S10A). Together, these data suggested that RBM15 was responsible for the m^6^A methylation of R-loops in prostate cancer.

### RBM15 and IGF2BPs play tumor suppressor roles in prostate cancer

The expression of IGF2BPs and RBM15 in PCa tissues and cell lines was assessed. In comparison to controls, the expression of IGF2BPs and RBM15 proteins varied in PCa tissues and cell lines (Fig. 8A and C, S10C, S11C, S11D). Given the similar expression patterns and effects on R-loop metabolism, we assume IGF2BPs and RBM15 could function as tumor suppressor in prostate cancer. Specifically, overexpression of individual IGF2BPs and RBM15 all markedly inhibited cell migration and proliferation viabilities in vitro and in vivo (Figs. 8D and G and 9A, D and F, S12A, B). Notably, KH3-4 and KH1-4 mutants could partially repair the inhibition of cell proliferation and migration capacities caused by IGF2BPs overexpression (Fig. 9C and D, S13A). Additionally, the inhibition of proliferation and migration viabilities of IGF2BPs overexpression cells could be partially reversed by RBM15 KO and forced expression of RNaseH1 (Fig.S9E, S13B-D), suggesting the suppressor role of IGF2BPs relies on the m^6^A modification and R-loop regulation.

#### SEMA3F play tumor suppressor roles in prostate cancer

Meanwhile, as IGF2BPs’ common target, SEMA3F was likewise downregulated in PCa tissues and cell lines (Fig. 10A, B and D). Remarkably as in PCa, individuals with low SEMA3F expression showed a significantly worse prognosis (Fig. 10C). Decreased proliferation and migration viabilities were also observed in SEMA3F overexpression cells as we expect (Fig. 10E and F). As shown recently, SEMA3s showed the special ability to activate Hippo pathway and attenuate tumorigenesis, angiogenesis and tissue growth. We observed similar results in SEMA3F and RBM15 overexpression PC-3 and DU-145 cells compared to control (Fig. 10I, S11G, I). Previous studies have indicated potential associations between Hippo pathway and docetaxel chemosensitivity [26–28], and we observed significant changes of docetaxel chemosensitivity in SEMA3F overexpression groups compared to controls (Fig. 10G and H). Meanwhile, the regulation of docetaxel chemosensitivity and Hippo pathway of SEMA3F overexpression cells could be partially reversed by LATS1 inhibitor (TDI-011536) (Fig. 10J and K, S11H). Altogether, our data showed that RBM15 and IGF2BPs play tumor suppressor roles via SEMA3F-mediated regulation of Hippo pathway in prostate cancer. Further evaluation of the mechanism for the RBM15/IGF2BPs/SEMA3F axis may provide a new strategy for prostate cancer.

## Discussion

The characterization of the IGF2BP proteins as direct m^6^A readers has provided novel insights into our understanding of effects of m^6^A on post-transcriptional regulation. However, the role of IGF2BPs on transcription is poorly understood. R-loops have initially been considered as by-products during transcription without any functional role. However, multiple of recent studies have revealed that they may have an effect on transcriptional regulation [6]. Here we demonstrated that IGF2BPs are extremely efficient in binding and regulating m^6^A-RNA methylated R-loops in prostate cancer. Surprisingly, we found that RBM15 was also important for R-loop recognition and metabolism, and recruits METTL3 to the R-loop for m^6^A RNA methylation. As expect, RBM15 knockout not only results in downregulation of m^6^A RNA methylation but also diminishes the interactions between IGF2BPs and R-loop. Furthermore, RBM15 depletion also impairs the biological function of IGF2BPs in prostate cancer.

.

IGF2BP proteins localized in the cytosol and the nucleus, while most studies focus on the cytosolic function [29–32]. Our previous study also revealed that IGF2BPs enhance the mRNA stability in the renal cancer [18]. Here, our finding illustrates for the first time that IGF2BPs play a crucial role in the nucleus. And the direct binding of IGF2BPs to m^6^A-modifed R-loops, as same as m^6^A RNAs, relies on their KH domains. Our studies demonstrated that KH domains, especially KH3-4 domain, are crucial for the regulation of R-loops and biological function of IGF2BPs. Notably, KH domain is a conserved RNA recognition element in other proteins such as HNRNPK, FMR1. It will be interesting that other proteins contain KH domain play a similar role in R-loop regulation.

Our results also illustrate the importance of IGF2BPs-meditated R-loops in gene regulation. IGF2BPs-meditated R-loops are commonly positioned at promoter region in PCa. IGF2BP proteins shared more than 50% RNA targets, but our data indicated only a small part (4-10%) of R-loops are shared by the three IGF2BP proteins. Additional researches will be required to further explore the relationship between IGF2BP proteins in R-loop regulation. Nevertheless, all IGF2BP proteins-mediated R-loops could protect SEMA3F promoter from DNA methylation via repelling DNMT1, as noted in another study [7].

Previous studies revealed the opposite role of IGF2BPs versus YTHDF2 on mRNA stabilization [17]. Similarly, our data showed that IGF2BPs are more likely to promote co-transcriptional R-loop formation while YTHDF2 eliminates m^6^A-containing R-loops. However, published ENCODE PAR-CLIP data demonstrated approximately 1% of IGF2BP binding sites being shared by YTHDF2, suggesting that R-loop metabolism and reader binding preferences may be attributed to the different m^6^A sites.

## Conclusion

We demonstrated that IGF2BPs could regulate R-loop metabolism in an m^6^A dependent way. In the nucleus, IGF2BP proteins prefers to bind the methylated DNA: RNA hybrids rather than ssRNA. Moreover, KH domains are crucial for the binding of IGF2BPs and R-loops. In addition, RBM15 is responsible for the m6A modification of R-loops. More importantly, our study provides a novel RBM15/IGF2BPs/DNMT1 trans-omics regulation m^6^A axis, indicating the new crosstalk between RNA m^6^A methylation and DNA methylation in prostate cancer. Simultaneously, as IGF2BPs co-target, SEMA3F was correlated with the survival of PCa patients, suggesting it might be a promising biomarker and predicting the risk of prostate cancer in the future.

### Supplementary Information

**Additional file 1: Supplementary Table S1.**

**Additional file 2: Supplementary Table S2.**

**Additional file 3: Supplementary Table S3. **Data analysis of RNA-seq and DRIP-seq Scramble vs IGF2BP1.

**Additional file 4: Supplementary Fig. 1. **(A). Isolated R-loops from RPWE-1 and other prostate cancer cell lines (LNCAP, VCAP, 22RV1, DU-145, PC-3) were analyzed by Dot-blot. (Left) representative dot-blot results; (Right): quantification of dot blot. (B). R-loop regulators were identified by LC-MS/MS analysis using S9.6 antibody in two previously published studies(Yellow: RNA/DNA Hybrid Interactome Identifies DXH9 as a Molecular Player in Transcriptional Termination and R-Loop-Associated DNA Damage; Orange: Human proteins that interact with RNA/DNA hybrids). The list of genes included in the published S9.6 IP results were compared using Venn diagram. (C). Validation of S9.6 IP LC-MS/MS results by western blot assay. (Left): representative western blot results; (Right): quantification of western blot. (D). PC-3 cell lysates were immunoprecipitated with IgG or anti-Myc-tagged antibody. Precipitates were blotted for IGF2BP1/2/3. (E). Quantification of western blot.

**Additional file 5: Supplementary Fig. 2. **(A). Schematic diagram of the IGF2BPs fragments. (B-D). Interaction of individual IGF2BP protein fragments and other full length IGF2BP proteins detected with Co-IP. (Left): Representative western blot results. (Right): Quantification of western blot.

**Additional file 6: Supplementary Fig. 3. **(A). Quantification of western blot results in Fig.2B. (B). Quantification of western blot results in Fig.2C. (C). Quantification of dot blot results in Fig.2F. Data are presented as means ± SD. (D). Quantification of dot blot results in Fig.2H. (E). CRISPR-Cas9 mediated KO of IGF2BP1/2/3 in PC-3 cells as detected by western blot. (Left): representative western blot results; (Right) :quantification of western blot.

**Additional file 7: Supplementary Fig. 4. **(A). IGF2BP1 KO PC-3 cells, as same as IGF2BP2 and IGF2BP3, were transfected with other full length IGF2BP proteins. The R-loop levels in each group were evaluated by dot-blot. (Upper): representative dot blot results; (Lower): quantification of dot blot. Data are presented as means ± SD, two-tailed unpaired t-test. (B). Quantification of western blot results in Fig.3C. *p-value < 0.05,**p-value < 0.01, ***p-value < 0.001,****p-value < 0.0001.

**Additional file 8: Supplementary Fig. 5. **(A). Quantification of western blot results in Fig.4A. (B). Quantification of western blot results in Fig.4B. (C). Quantification of western blot results in Fig.4C. (D). Quantification of dot blot results in Fig.4D. Data are presented as means ± SD. (E). Quantification of western blot results in Fig.4D. (F). Quantification of western blot results in Fig.6D. (G). Quantification of western blot results in Fig.6E. (H). Quantification of western blot results in Fig.7A.

**Additional file 9: Supplementary Fig. 6.** (A). Principal component analysis (PCA) constructed using the information from RNA-seq simultaneously. (B). The list of genes included in the DRIP-seq results and published PAR-CLIP-seq results were compared using Venn diagram. (C). The coverage plots of S9.6 DRIP densities in the promoter region of the indicated gene (PABPC1). (D). Relative expression of SEMA3F mRNA in IGF2BPs overexpression PC-3 cells compared to control by RT-qPCR. Data are presented as means ± SD, two-tailed unpaired t-test. (E). Effect of IGF2BPs KO on SEMA3F protein levels. (Upper): Representative western blot results; (Lower): Quantification of western blot.

**Additional file 10: Supplementary Fig. 7.** (A). Quantification of dot blot results in Fig.3B. Data are presented as means ± SD. (B). The ssDNA and RNA:DNA hybrid probes with methylated (red) or unmethylated (green) adenosine were shown. (C). Effect of RNaseH1 overexpression on endogenous IGF2BPs-induced SEMA3F upregulation in western blot assays. (Upper): representative western blot results; (Lower): quantification of western blot. (D). Correlation of mRNA expression between IGF2BPs and SEMA3F from GEPIA portal determined by Pearson coefficient. *p-value < 0.05,**p-value < 0.01, ***p-value < 0.001,****p-value < 0.0001. 

**Additional file 11: Supplementary Fig. 8.** (A-B). Global DNA 5mC levels in IGF2BP-KO cells treated with wild-type or mutated IGF2BPs. A: Representative dot blot results are shown. B: Quantification of dot blot results. Data are presented as means ± SD, two-tailed unpaired t-test. (C). Effect of IGF2BPs overexpression on DNMT1 in western blot assays. (Upper): Representative western blot results; (Lower): Quantification of western blot. (D). Methylation level changes of CpG sites on the IGF2BPs co-targets promoter by IGF2BPs overexpression. (E). Correlation of mRNA expression between DNMTs and SEMA3F from GEPIA portal determined by Pearson coefficient.*p-value < 0.05, **p-value < 0.01, ***p-value < 0.001,****p-value < 0.0001.

**Additional file 12: Supplementary Fig. 9.** (A). PC-3 cells were immunostained for RBM15 and anti-m^6^A antibody; representative images are shown (scale bar: 20μm). (B). RBM15 KO PC-3 cells were transfected by Myc-IGF2BPs and vector, and the R-loop levels were assessed by dot-blot. (Left) representative dot-blot results; (Right): quantification of dot blot. Data are presented as means ± SD, two-tailed unpaired t-test. (C). Quantification of dot blot results in Fig.7F. Data are presented as means ± SD, two-tailed unpaired t-test. (D). Quantification of dot blot results in Fig.7H. Data are presented as means ± SD, two-tailed unpaired t-test. (E). Bioinformatics site SRAMP predicts m^6^A methylation sites in the sequence of SEMA3F promoter. (F). 293T purified Flag-METTL3 protein was mixed with 293T purified HA-RBM15 protein. The mixture was immunoprecipitated with IgG, anti-Flag-tagged and anti-HA-tagged antibody. Precipitates were blotted for Flag-purified METTL3 and HA-purified RBM15. (Upper): Representative western blot results. (Lower): Quantification of western blot assay. (G). R-loop levels of SEMA3F promoter in IGF2BPs overexpression RBM15-KO PC-3 cells compared to control by DRIP-qPCR. RNaseH1 treated samples were used as negative control. Data are presented as means ± SD, two-tailed unpaired t-test. *p-value < 0.05, **p-value < 0.01,***p-value < 0.001, ****p-value < 0.0001.

**Additional file 13: Supplementary Fig. 10.** (A). RBM15 KO PC-3 cells were transfected by Myc-IGF2BPs and vector, and the expression of SEMA3F was evaluated by western blot. (Upper): Representative western blot results; (Lower): Quantification of western blot. (B). Identification of R-loop binding proteins by S9.6 IP and western blot assay. Experiments were performed with Flag-purified METTL3 protein, IgG antibody, RBM15 antibody and S9.6 antibody in PC-3 cell lysates. (Upper): Representative western blot results. (Lower): Quantification of western blot assay. (C). IGF2BP proteins were determined in PCa samples and paired adjacent normal tissues (n=6). (Left): Representative western blot results. (Right): Quantification of western blot assay. Data are presented as means ± SD, two-tailed unpaired t-test. (D). PC-3 cells were transfected with negative control and METTL3 siRNA. The R-loop levels were assessed by dot-blot. (Left): Representative dot blot results. (Right): Quantification of dot blot assay. Data are presented as means ± SD. (E). Correlation of mRNA expression between RBM15 and SEMA3F from GEPIA portal determined by Pearson coefficient. *p-value < 0.05,**p-value < 0.01, ***p-value < 0.001,****p-value < 0.0001.

**Additional file 14: Supplementary Fig. 11.** (A). Quantification of western blot results in Fig.7B. (B). Re-ChIP analysis in PC-3 cells with anti-RBM15 and anti-m^6^A antibodies. RNaseH1 treated samples were used as negative control. Data are presented as means ± SD, two-tailed unpaired t-test. (C). Quantification of western blot results in Fig.8C. (D). Quantification of western blot results in Fig.10D. (E). Pulldown followed by western blot indicated in vitro binding of m^6^A-modified RNA:DNA hybrid probe (using SEMA3F promoter sequence) with YTHDF2 in control and IGF2BPs overexpression PC-3 cells. (Left): Representative western blot results. (Right): Quantification of western blot assay. (F). The levels of SEMA3F promoter in IGF2BPs overexpression DU-145 and PC-3 cells compared to control by ChIP-qPCR (using YTHDF2 antibody and IgG antibody), Data are presented as means ± SD, two-tailed unpaired t-test. (G). Quantification of western blot results in Fig.10J. (H). Quantification of western blot results in Fig.10K. (I). Effect of RBM15 overexpression on Hippo pathway in DU-145 and PC-3 cells by western blot assay. (Left): Representative western blot results. (Right): Quantification of western blot assay. (J). Effect of YTHDF2 knockdown on SEMA3F protein levels in PC-3 cells by western blot assay. (Upper): Representative western blot results. (Lower): Quantification of western blot assay.

**Additional file 15: Supplementary Fig. 12.** (A). Immunohistochemical labelling showed the downregulated expression of Ki-67 after RBM15 or IGF2BPs overexpression (A)(scale bar: 100μm). (B). Immunohistochemical labelling showed the overexpression of RBM15 and IGF2BPS after RBM15 or IGF2BPs overexpression (scale bar: 100μm).

**Additional file 16: Supplementary Fig. 13. **(A). CCK-8 assays revealed the cell proliferation abilities of IGF2BP-KO cells treated with wild-type or mutated IGF2BPs. Data are presented as means ± SD. (B). Clone formation capacities of IGF2BPs overexpression RBM15 KO PCa cells were assessed by the clone formation assay compared to control. Data are presented as means ± SD, two-tailed unpaired t-test. (C). Effect of RNaseH1 overexpression on endogenous IGF2BPs-induced inhibition of clone formation capacities in DU-145 cells. (D). Effect of RNaseH1 overexpression on endogenous IGF2BPs-induced inhibition of cell migration capacities in DU-145 and PC-3 cells.

**Additional file 17.**

## Data Availability

No datasets were generated or analysed during the current study.
