# Supplementary material for: Co-transcriptional R-loops-mediated epigenetic regulation drives growth retardation and docetaxel chemosensitivity enhancement in advanced prostate cancer
Source: Mol Cancer. 2024 Apr 24;23:79. doi: 10.1186/s12943-024-01994-0 (PMC11041046; doi:10.1186/s12943-024-01994-0)
Supplement: Supplementary file 1 — Additional file 1: Supplementary Table S1. [file 12943_2024_1994_MOESM1_ESM.docx]

**Supplementary Table S1.**

| **Primers used for Real-time qPCR** | |
| --- | --- |
| **Name** | **Sequence** |
| IGF2BP1 forward | GCGGCCAGTTCTTGGTCAA |
| IGF2BP1 reverse | TTGGGCACCGAATGTTCAATC |
| IGF2BP2 forward | AGCTAAGCGGGCATCAGTTTG |
| IGF2BP2 reverse | CCGCAGCGGGAAATCAATCT |
| IGF2BP3 forward | TATATCGGAAACCTCAGCGAGA |
| IGF2BP3 reverse | GGACCGAGTGCTCAACTTCT |
| RBM15 forward | ACGACCCGCAACAATGAAG |
| RBM15 reverse | GGAAGTCGAGTCCTCACCAC |
| SEMA3F forward | AACACAACCGACTACCGAATC |
| SEMA3F reverse | GGCTGCCCAGTGTATAATGAG |
| **Primers used for m^6^A DIP, S9.6 DRIP and DNMT1 ChIP** | |
| **Name** | **Sequence** |
| SEMA3F forward | TTCTGCCTCAGCTCTCTCTC |
| SEMA3F reverse | AGTATACCGCTTCCCTTCGG |
| SEMA3F-m^6^A forward | GGAGAAGGAAGGAGAGGCAGAGG |
| SEMA3F-m^6^A reverse | TGCCCAACCCAACTTTCCAAGG |
| **Oligonucleotides used for pull-down** | |
| **Name** | **Sequence** |
| m^6^A-ssRNA oligo | CGUCUCGG(m6A)CUCGG(m6A)CUGCU |
| ssRNA oligo | CGUCUCGGACUCGGACUGCU |
| DNA oligo | AGCAGTCCGAGTCCGAGACG |
| **sgRNAs used for CRISPR-Cas9** | |
| **Name** | **Sequence** |
| sg-IGF2BP1 | ATATTCCACCCCAGCTCCGA |
| sg-IGF2BP2 | ATGCCCGCTTAGCTTCTCCA |
| sg-IGF2BP3 | ATATCCCGCCTCATTTACAG |
