# Supplementary material for: Co-transcriptional R-loops-mediated epigenetic regulation drives growth retardation and docetaxel chemosensitivity enhancement in advanced prostate cancer
Source: Mol Cancer. 2024 Apr 24;23:79. doi: 10.1186/s12943-024-01994-0 (PMC11041046; doi:10.1186/s12943-024-01994-0)
Supplement: Supplementary file 2 — Additional file 2: Supplementary Table S2. [file 12943_2024_1994_MOESM2_ESM.docx]

**Supplementary Table S2.**

Clinical data of the PCa patients (n=36)

| **No.** | **Sex（M/F）** | **Pathologic diagnosis** | **Gleason** |
| --- | --- | --- | --- |
| 1 | Male | Prostate adenocarcinoma | 4+3=7 |
| 2 | Male | Prostate adenocarcinoma | 3+4=7 |
| 3 | Male | Prostate adenocarcinoma | 3+3=6 |
| 4 | Male | Prostate adenocarcinoma | 4+3=7 |
| 5 | Male | Prostate adenocarcinoma | 4+5=9 |
| 6 | Male | Prostate adenocarcinoma | 3+4=7 |
| 7 | Male | Prostate adenocarcinoma | 4+4=8 |
| 8 | Male | Prostate adenocarcinoma | 4+3=7 |
| 9 | Male | Prostate adenocarcinoma | 3+5=8 |
| 10 | Male | Prostate adenocarcinoma | 4+3=7 |
| 11 | Male | Prostate adenocarcinoma | 4+3=7 |
| 12 | Male | Prostate adenocarcinoma | 5+4=9 |
| 13 | Male | Prostate adenocarcinoma | 4+3=7 |
| 14 | Male | Prostate adenocarcinoma | 4+4=8 |
| 15 | Male | Prostate adenocarcinoma | 3+4=7 |
| 16 | Male | Prostate adenocarcinoma | 5+4=9 |
| 17 | Male | Prostate adenocarcinoma | 3+4=7 |
| 18 | Male | Prostate adenocarcinoma | 3+4=7 |
| 19 | Male | Prostate adenocarcinoma | 5+5=10 |
| 20 | Male | Prostate adenocarcinoma | 4+3=7 |
| 21 | Male | Prostate adenocarcinoma | 4+5=9 |
| 22 | Male | Prostate adenocarcinoma | 4+4=8 |
| 23 | Male | Prostate adenocarcinoma | 3+4=7 |
| 24 | Male | Prostate adenocarcinoma | 4+4=8 |
| 25 | Male | Prostate adenocarcinoma | 3+4=7 |
| 26 | Male | Prostate adenocarcinoma | 3+3=6 |
| 27 | Male | Prostate adenocarcinoma | 3+4=7 |
| 28 | Male | Prostate adenocarcinoma | 4+4=8 |
| 29 | Male | Prostate adenocarcinoma | 4+3=7 |
| 30 | Male | Prostate adenocarcinoma | 3+4=7 |
| 31 | Male | Prostate adenocarcinoma | 3+4=7 |
| 32 | Male | Prostate adenocarcinoma | 4+5=9 |
| 33 | Male | Prostate adenocarcinoma | 3+3=6 |
| 34 | Male | Prostate adenocarcinoma | 4+3=7 |
| 35 | Male | Prostate adenocarcinoma | 3+4=7 |
| 36 | Male | Prostate adenocarcinoma | 4+3=7 |
