# Supplementary material for: Co-transcriptional R-loops-mediated epigenetic regulation drives growth retardation and docetaxel chemosensitivity enhancement in advanced prostate cancer
Source: Mol Cancer. 2024 Apr 24;23:79. doi: 10.1186/s12943-024-01994-0 (PMC11041046; doi:10.1186/s12943-024-01994-0)
Supplement: Supplementary file 3 — Additional file 3: Supplementary Table S3. Data analysis of RNA-seq and DRIP-seq Scramble vs IGF2BP1. [file 12943_2024_1994_MOESM3_ESM.docx]

**Supplementary Table S3.** Data analysis of RNA-seq and DRIP-seq

Scramble vs IGF2BP1

| **Gene symbol** | **Log2 ratio of transcript** | **Log2 ratio of R-loop density** | **Gene symbol** | **Log2 ratio of transcript** | **Log2 ratio of R-loop density** |
| --- | --- | --- | --- | --- | --- |
| SEMA3F | 0.83893 | 0.32732 | BCL2L1 | -1.03785 | 0.92856 |
| BTBD7 | -0.16258 | -1.55250 | EIF2AK3 | -1.74147 | -1.05606 |
| WWTR1 | -0.42617 | -0.54930 | NME6 | -0.39046 | 1.57160 |
| PRDM11 | -1.33591 | 1.49878 | CFL1 | 0.64894 | 0.13241 |
| MNAT1 | 0.31763 | -0.54145 | RARG | -0.45396 | 0.75612 |
| SAMD4A | -0.44738 | -0.53509 | ZNF680 | 0.62162 | 0.34604 |
| PRKCH | -0.05060 | -0.65133 | XXYLT1 | -0.47596 | 1.00886 |
| STAU2 | 0.67910 | -0.47964 | MRPS22 | 0.69619 | 1.27693 |
| POLR2B | -0.03730 | 0.88369 | RAB6A | -0.49124 | 2.18669 |
| PARP12 | -0.45605 | 1.62920 | TCP11L1 | 0.70123 | 0.92856 |
| INTS13 | -0.10506 | 0.62761 | FAM91A1 | -1.10302 | -0.61353 |
| FAR2 | -2.09811 | 0.55558 | PPFIA3 | 0.30337 | 0.62285 |
| PDIA5 | -0.31194 | 0.49518 | PARP10 | -0.09381 | 0.06589 |
| PDE4A | 1.35289 | 0.69166 | R3HDM2 | -0.53963 | 0.57770 |
| NGEF | 0.49295 | 0.92856 | ZNF678 | -0.63631 | 0.74897 |
| KCNAB2 | 1.03195 | 1.14610 | RAD51B | 0.79292 | -0.46145 |
| NUCB2 | -1.09987 | 1.07436 | C8orf33 | 0.75716 | 0.73951 |
| PABPC1 | 0.78502 | 0.71645 | EXOC7 | -0.40340 | 0.37454 |
| RDH11 | 0.42721 | 0.89933 | ZNF623 | -0.30733 | 0.37708 |
| STK10 | 0.49569 | 0.60139 | CCSER1 | -0.73991 | -1.14616 |
| IGF2BP2 | -0.24177 | 1.30296 | MAML2 | 0.47757 | 2.16538 |
| ACTB | 0.62206 | 0.93884 | FOXO4 | -1.33475 | 0.91309 |
| TPD52 | -0.12977 | -0.40142 | POLR3C | -1.09612 | 1.33776 |
| NEBL | -1.27060 | 1.07436 | POLR1D | -0.75248 | 2.30079 |
| XRCC5 | 0.71745 | 0.73951 | BACE1 | 0.01713 | 0.55558 |
| AACS | -0.09543 | 0.31783 | RTN4RL2 | 2.47549 | 1.32121 |
| PLOD1 | 0.35673 | 0.76122 | FANCA | 1.45211 | -1.17889 |
| MTIF2 | 0.55985 | 1.48374 | UBQLN2 | 0.37257 | 1.17593 |
| SLC23A2 | 0.61842 | 0.51109 | ZNF490 | -1.18342 | 0.96102 |
| PXN | -0.34845 | 0.88369 | COL4A5 | -0.71756 | 1.92623 |
| NECAP1 | -0.83471 | 0.89569 | PRKAR1B | 0.23955 | 0.74897 |
| GPATCH2L | 0.38369 | -0.43997 | ZP3 | 0.68900 | 1.01175 |
| NUDC | 0.53199 | 0.43225 | RALGAPA2 | 0.45986 | 0.92856 |
| CERS4 | 0.54444 | 0.69605 | CLN3 | -0.60130 | 1.11388 |
| OSBPL8 | -0.53815 | -1.31676 | CLDN4 | 0.47679 | 0.81986 |
| MAP3K20 | 0.83994 | 0.76620 | JPT1 | 3.03887 | 0.73425 |
| ZFHX4 | -1.88935 | 1.37631 | C8orf76 | -0.51364 | 0.67878 |
| FKBP5 | 0.96629 | 1.16027 | SEMA4A | 0.35450 | 1.12128 |
| SIRT1 | -1.17413 | -0.59333 | ZNF512B | -0.33488 | 0.56919 |
| GHITM | -0.41664 | -1.05606 | PSMD12 | 0.33224 | 0.80713 |
| SPINT1 | 0.01548 | -0.49067 | SIPA1L1 | -0.68647 | -0.78656 |
| HPS6 | 1.15888 | 0.97200 | RPE | 0.62142 | 1.15796 |
| USP54 | -1.44709 | 0.73429 | ZNF71 | -0.16360 | 0.44980 |
| RNF169 | -0.49784 | 0.93353 | HYLS1 | 0.61262 | 0.47356 |
| NDST2 | -0.20034 | 0.57852 | ASPH | 0.40746 | 1.37121 |
| FAM111A | 2.21795 | 0.31659 | WWP2 | -0.40878 | -0.94504 |
| TSC22D4 | -0.23521 | 0.97320 | BAZ1A | 1.05145 | 0.57770 |
| SMAD3 | 0.80533 | 1.45052 | NCOA6 | -0.45090 | -1.20668 |
| CDK12 | -0.53095 | -1.45337 | ZNF358 | -0.33757 | 0.53559 |
| TUBA1C | 0.79936 | 1.12128 | SYS1 | -0.23547 | 0.91078 |
| AXL | -0.25507 | 1.27693 | DDR1 | 0.28516 | 1.24858 |
| RAB4A | 0.55734 | -0.59598 | AKT1S1 | -0.03971 | 0.76226 |
| ZSWIM1 | 0.45369 | 1.54365 | GABBR1 | 0.44095 | 1.27735 |
| CPT1C | 1.56856 | 1.09463 | TECPR1 | 0.68285 | 1.37121 |
| RGS14 | 1.11360 | 0.71645 | CRYZL1 | 0.63960 | 1.27693 |
| GLB1 | -0.14804 | -1.26415 | SLC48A1 | -0.31567 | 2.73720 |
| KRT8 | -0.16964 | 2.50389 | CLIC1 | -0.08684 | 0.86078 |
| SCAND1 | -0.19544 | -0.24820 | CEBPZOS | 0.20171 | -0.90133 |
| FAM241B | 0.66686 | 0.78058 | NSUN6 | -0.60940 | -0.48526 |
| TAF15 | 0.35994 | 0.51005 |  |  |  |

Scramble vs IGF2BP2

| **Gene symbol** | **Log2 ratio of transcript** | **Log2 ratio of R-loop density** | **Gene symbol** | **Log2 ratio of transcript** | **Log2 ratio of R-loop density** |
| --- | --- | --- | --- | --- | --- |
| SEMA3F | 0.53717 | 0.48047 | MLPH | -0.96696 | -1.01562 |
| ALS2 | 1.70796 | -0.14870 | EPHA4 | 0.67973 | -0.78613 |
| PLXND1 | 1.47727 | 1.52346 | ARHGEF2 | 0.82518 | -1.51374 |
| NFIX | 1.52010 | -0.47182 | PIK3R3 | 0.79706 | -0.39209 |
| POMT2 | 0.68058 | 0.59438 | PRRC2C | 0.44723 | -0.28122 |
| BAZ1B | 1.32397 | 0.18522 | OSBPL9 | -0.79944 | 0.30476 |
| WWTR1 | -0.73354 | -0.22251 | DDX59 | 0.58930 | -0.43469 |
| RNF10 | 1.00865 | -0.65262 | MLH3 | 0.59253 | 0.85963 |
| APBA2 | 1.21474 | 0.34175 | ATL2 | -0.67861 | 0.51225 |
| CASR | 0.65328 | -0.37753 | TRIM25 | -0.43483 | -0.88718 |
| PHLPP2 | -1.30810 | 0.29268 | ZMYM2 | 1.50996 | -0.29776 |
| POLQ | 0.54540 | 1.90342 | SERAC1 | 1.55840 | -0.27980 |
| CUL1 | 0.73331 | 0.52677 | CCDC18 | -0.62438 | 0.66825 |
| CYFIP2 | 1.04933 | -0.31745 | CALD1 | -0.86048 | 0.62563 |
| CDK14 | -1.15366 | 0.89534 | ITPR2 | 0.90214 | 0.94402 |
| ZC3H11A | -0.70877 | -0.55758 | WWP1 | -0.72958 | 0.26599 |
| INTS13 | 0.70354 | -0.11857 | DDX39A | 0.95207 | 0.35021 |
| PDE4A | 1.75746 | 1.01969 | NR4A1 | 2.03507 | -0.95434 |
| NGEF | 0.95207 | -1.55271 | STIL | 0.83474 | 1.08571 |
| TMEM260 | 0.85077 | -0.22424 | ARFGEF2 | 0.18945 | -0.53775 |
| PABPC1 | 0.85077 | 0.50731 | BTN2A2 | 0.85572 | 0.43532 |
| ADGRL1 | 1.44513 | 0.28577 | LRRFIP1 | 0.94619 | -0.09659 |
| STK10 | 0.54909 | 0.36323 | INSIG2 | -1.48731 | 0.58093 |
| FERMT2 | -0.55208 | -1.59811 | TNFSF9 | 0.54477 | 0.62086 |
| MAP3K13 | 0.89192 | -0.16658 | ERGIC3 | 0.59147 | -0.21110 |
| ZNF532 | -0.83048 | -0.21516 | SIX1 | 0.54477 | -0.59584 |
| ACTB | 0.71964 | 0.38017 | PLEKHG3 | 1.08147 | 0.53708 |
| BAZ2A | 1.05612 | -0.11397 | TEP1 | 1.07962 | 0.57065 |
| ARHGEF1 | 1.00865 | 0.20052 | DOCK6 | 0.27059 | 0.30072 |
| TM9SF3 | 0.98164 | -0.38869 | SLC27A1 | -0.39251 | 0.99421 |
| MAP2 | -1.59738 | 0.96594 | FCHO1 | -1.18901 | 0.97250 |
| NEBL | -1.26278 | -1.55907 | MAP1S | 1.32397 | 0.43038 |
| SAR1A | 0.66464 | -0.02968 | TRPM4 | 1.04553 | 0.86190 |
| PLOD1 | 0.54113 | 0.30965 | HIP1R | 0.67630 | 0.42951 |
| EIF3I | 1.58717 | 0.32494 | ACSS2 | 0.85815 | -0.88920 |
| PPP1R15A | 1.08952 | -1.84891 | RFTN1 | 1.69951 | 2.51923 |
| PIR | -0.75529 | -0.47791 | EXOC4 | -0.80406 | -0.36033 |
| DOCK9 | 1.63910 | -0.31061 | ZNF304 | 1.04474 | -0.78716 |
| KIF9 | -1.18901 | -0.51222 | BTBD2 | -0.93741 | -0.20098 |
| GPATCH2L | -0.39107 | 0.75046 | MKRN1 | 1.53149 | -0.03254 |
| SLC9A1 | 0.45489 | -0.69458 | TMEM254 | 0.59272 | -1.11969 |
| CCDC80 | -0.36073 | 2.39289 | NUMB | 0.90693 | -0.01282 |
| EFHC1 | 0.44723 | -0.60920 | MBD2 | -0.66424 | -0.37706 |
| SIRT1 | 0.58695 | -0.85605 | TMEM165 | 0.77164 | -0.31076 |
| MZF1 | 0.41026 | -1.37008 | GDF11 | 0.67011 | 1.05773 |
| HIF1A | 0.41301 | 0.15108 | UGGT1 | 0.94619 | 0.06441 |
| DICER1 | 0.80839 | -0.09723 | ARRB1 | 1.84326 | 1.44464 |
| SEC23A | -0.54610 | 0.07791 | NUMA1 | 1.12775 | 0.24398 |
| FNDC3A | 1.44070 | -0.72731 | TRIM29 | 0.47356 | -0.83692 |
| COG4 | 0.98164 | -0.02214 | RDX | 0.79706 | 0.42430 |
| CMC2 | 2.02472 | 1.04782 | ARHGAP29 | -0.42986 | 0.30481 |
| COTL1 | 0.89799 | 0.17544 | HADHB | -0.72408 | -0.30155 |
| CALB1 | 0.62003 | -1.34735 | FAM149B1 | -0.77207 | -0.79020 |
| CLASRP | 1.04553 | 0.21178 | PARP9 | 1.42191 | 0.19447 |
| SNX8 | -0.27311 | 0.68991 | AMIGO2 | 0.92461 | 0.16358 |
| AHR | -0.97019 | 0.22213 | GIT2 | 1.36789 | -0.28525 |
| LIMK1 | 1.06433 | 0.86591 | DENR | 0.96725 | -0.13592 |
| TMEM245 | 1.21001 | 0.27740 | CDH24 | 1.76504 | 1.42604 |
| KPNB1 | 1.04553 | 0.10657 | PCTP | -0.80406 | 0.61815 |
| LUC7L3 | 0.62428 | 0.70647 | AFG3L2 | -0.43614 | 0.33972 |
| JHY | 1.69951 | -0.59978 | COL6A1 | 0.47865 | 0.59907 |
| ST3GAL4 | 0.92941 | -0.55550 | KIAA0319L | -0.89485 | -0.47217 |
| BCL7A | 0.77164 | -1.08316 | UBAP2L | 1.58717 | -0.08015 |
| LTA4H | 0.76471 | -0.14043 | HAX1 | 1.29790 | -0.36876 |
| CREBL2 | 0.67973 | 0.38306 | SNAP47 | 1.01816 | 0.28410 |
| ATN1 | 0.97448 | -0.69845 | CDC42BPA | -0.61003 | -0.26856 |
| CEP72 | -0.53551 | 0.64345 | PARP1 | -0.56569 | 0.89971 |
| PLSCR4 | 1.90636 | -1.87141 | VGLL4 | 0.06002 | -0.42662 |
| CLCN2 | 1.02191 | 0.64391 | COL8A1 | -0.86048 | 1.24462 |
| LMAN2L | 0.52005 | -0.20518 | LHFPL2 | -0.90200 | -0.33731 |
| GTF3C2 | -1.04930 | 0.46124 | MRPL19 | 1.04474 | 0.98348 |

Scramble vs IGF2BP3

| **Gene symbol** | **Log2 ratio of transcript** | **Log2 ratio of R-loop density** | **Gene symbol** | **Log2 ratio of transcript** | **Log2 ratio of R-loop density** |
| --- | --- | --- | --- | --- | --- |
| PLCB4 | -0.85003 | -0.62248 | KIF2C | 1.01964 | 0.75669 |
| ZNF133 | -0.87332 | 0.18178 | MUTYH | 0.71767 | 0.72204 |
| OSBPL9 | -0.84229 | 0.47144 | TESK2 | 0.82439 | 0.38058 |
| USP24 | -0.84901 | -0.43703 | POMGNT1 | 1.22684 | 0.89830 |
| AP1G2 | 1.85855 | 0.58154 | LRRC41 | 1.63249 | -0.50400 |
| IFI44 | -1.40251 | -0.47950 | ATPAF1 | 1.88308 | -0.07166 |
| ARHGAP29 | -0.36621 | 0.72679 | ABCD3 | 0.82439 | 0.71291 |
| SLC4A7 | -2.03563 | -0.29423 | MOV10 | 1.01029 | -0.09260 |
| KIF9 | -0.84901 | -1.00827 | ATP1A1 | 1.52430 | 0.46388 |
| ARHGEF1 | 1.13228 | 0.25507 | TRIM45 | 0.56787 | 1.01792 |
| ARHGEF18 | 1.38096 | -0.34285 | CERS2 | 1.41925 | -0.40772 |
| HADHB | -0.71911 | -0.24992 | GATAD2B | 1.15099 | -0.23865 |
| GTF3C2 | -0.92295 | 0.54060 | RAB13 | 0.69243 | 1.10969 |
| CEBPZOS | -0.87332 | 0.09806 | TPM3 | 0.46841 | 0.55029 |
| CCDC88A | -1.23881 | -0.25511 | UBAP2L | 1.49071 | -0.36158 |
| INSIG2 | -1.73738 | 0.78855 | ZBTB7B | 1.60375 | -1.22658 |
| ASAP1 | 0.42453 | 0.08249 | THBS3 | 0.88876 | 0.44596 |
| LHFPL2 | -0.78287 | -0.17770 | RIT1 | 1.28208 | -0.71180 |
| ERGIC1 | -0.75455 | 0.40517 | LTBP3 | 2.06095 | -0.07583 |
| HTT | -0.88385 | 0.14922 | IQGAP3 | 0.92523 | 1.62024 |
| FIP1L1 | -0.66434 | -0.26841 | GPATCH4 | 1.29414 | -0.27911 |
| IGF2BP3 | 0.73908 | 4.06718 | ARHGEF11 | 1.25290 | -0.75691 |
| NDUFA5 | -1.44247 | 0.57341 | NECTIN4 | 0.67644 | 0.94373 |
| AGK | -0.42966 | 0.51459 | MAP3K20 | 1.05001 | 1.21361 |
| CDC42BPA | 1.15411 | -0.49848 | INAVA | 1.23695 | 0.43991 |
| ADGRG6 | -1.91248 | -1.00881 | ATP2B4 | 1.10969 | 0.33225 |
| CDK5RAP3 | 1.36868 | -0.29094 | CDK18 | 0.87307 | 1.36492 |
| CEP192 | 1.21048 | 0.15184 | MBD5 | 1.04739 | 0.09331 |
| CERS4 | 0.75770 | 0.84800 | CENPF | 1.20014 | 0.78943 |
| YWHAZ | -0.81775 | 0.56327 | MED12 | 1.65174 | 0.22285 |
| FAM111A | -0.53466 | 1.86748 | MIA3 | 0.55714 | -0.81683 |
| DPP3 | -0.68477 | 0.53973 | SRP9 | 0.94762 | 0.45192 |
| CFL1 | 0.27602 | 0.51496 | MEIS2 | 1.67050 | -1.04275 |
| RAB30 | -0.52142 | -0.31285 | HNRNPU | 0.97766 | -0.03179 |
| ANK3 | -1.15717 | -0.83500 | CAMK1 | 1.46969 | 0.54441 |
| CDK1 | -0.64536 | 1.25106 | SLC6A11 | 0.91348 | -0.11728 |
| GHITM | -1.14392 | -0.50947 | RFTN1 | 1.88308 | 3.21771 |
| COG5 | 1.16944 | 0.37616 | NR1D2 | 1.52674 | -0.33915 |
| SPATS2 | -0.78287 | -0.67042 | OSBPL10 | 1.12506 | -0.56797 |
| TYRO3 | -0.75610 | 0.59867 | LRRFIP2 | 0.93521 | -0.15256 |
| SEC23A | -0.74929 | 0.15352 | GORASP1 | 0.98233 | 0.41606 |
| GPATCH2L | -0.52142 | 0.74725 | NICN1 | 0.99678 | 0.82410 |
| BTBD7 | -1.32191 | 0.05209 | ARF4 | 1.03374 | -0.65665 |
| SEC14L1 | -0.81966 | 0.00731 | ATXN7 | 1.66916 | -0.50642 |
| USP7 | -1.51176 | 0.15576 | DCBLD2 | 1.80393 | 0.64190 |
| FANCA | -0.77082 | 0.98524 | TMEM45A | -0.78287 | -0.35304 |
| ZNF121 | -1.90484 | -0.71201 | USF3 | 0.53145 | -0.19562 |
| FCHO1 | -0.75455 | 1.03941 | GSK3B | 0.75942 | 0.15650 |
| MYADM | -0.78952 | -0.56830 | POLQ | 0.65799 | 1.58046 |
| MBD2 | -1.42668 | -0.23877 | PLSCR4 | 1.94537 | -1.63169 |
| CDH7 | -1.81439 | -1.33360 | MBNL1 | 2.10722 | -0.47608 |
| SLC23A2 | 0.68886 | 0.32126 | CLCN2 | 0.84389 | 0.60664 |
| ARFGEF2 | 0.45105 | -0.59849 | BDH1 | 0.71003 | 0.47905 |
| DOCK6 | 0.35251 | 0.43716 | NCOA1 | 0.97688 | -0.33286 |
| PTAFR | 0.65799 | 3.85361 | CRIM1 | 1.46699 | -0.06428 |
| PTPRF | 0.64610 | 0.63568 | SRSF7 | 1.60344 | 0.58430 |
| CMPK1 | 0.94329 | 0.47270 | PELI1 | 1.13228 | 0.74123 |
| DROSHA | 1.44954 | -0.13038 | PPP3R1 | 2.15226 | -0.73463 |
| ANP32E | 0.49901 | 0.82268 | INPP4A | 1.46699 | 0.46894 |
| ILF2 | -0.61153 | 0.33420 | UNC50 | 1.20014 | -0.44568 |
| DAP3 | 0.70714 | 0.27701 | RBMS1 | 1.23688 | 0.29815 |
| VGLL4 | -0.12061 | -0.33669 | SP3 | 1.74590 | 0.35651 |
| SEMA3F | 0.29496 | 0.83600 | NRP1 | 0.21155 | -0.52401 |
| ETFB | 1.21048 | 0.52683 | RPL37A | 0.81250 | 0.73703 |
| EXOC4 | -0.78287 | 0.18900 | CYFIP2 | 0.63810 | 0.11841 |
| GORASP2 | 0.38521 | -0.18077 | RNF145 | 1.69261 | -0.86515 |
| SLC25A46 | -0.74411 | 0.10616 | CPEB4 | 0.63401 | -0.75379 |
| UMAD1 | 0.35018 | 0.35078 | NUDC | 0.41610 | 0.38565 |
| ZNF107 | 0.53428 | 0.89108 | TMEM165 | 1.14103 | -0.34031 |
| BTN2A2 | 0.44827 | 0.44412 | PRKAR1B | 0.74897 | 0.23955 |
| FAM149B1 | -1.16314 | -0.54835 | FOXK1 | 1.09042 | -0.19328 |
| USP49 | 0.46781 | -0.34498 | ACTB | 0.93884 | 0.62206 |
| ZNF462 | 0.55751 | 0.28408 | RAC1 | 1.01411 | 0.32728 |
| EXD3 | -0.76390 | -0.31603 | PARP12 | 1.62920 | -0.45605 |
| SDC2 | 0.81846 | -0.96823 | EGFR | 1.20468 | 0.34095 |
| ANKRD46 | 0.47222 | -0.35166 | ZP3 | 1.01175 | 0.68900 |
| ATAD2 | -0.50295 | 1.57158 | PCNX2 | 0.48166 | -0.85678 |
| GABBR1 | 2.09622 | 0.45524 | TECPR1 | 1.37121 | 0.68285 |
| PARP10 | 0.04889 | -0.05055 | TSC22D4 | 0.97320 | -0.23521 |
| GDAP1 | 1.93310 | 0.75221 | PRKCH | -0.65133 | -0.05060 |
| NHS | -0.34460 | 1.02428 | NSD3 | 0.96445 | -0.35331 |
| GLB1 | -1.16746 | 0.19492 | ASPH | 1.37121 | 0.40746 |
| NXF1 | 0.40692 | -1.35830 | ZFHX4 | 1.37631 | -1.88935 |
| GRAMD1B | 1.61949 | 0.87673 | PABPC1 | 0.71645 | 0.78502 |
| UBASH3B | 0.48632 | 1.51109 | RABL3 | -0.69365 | 0.41093 |
| CLMP | 0.73308 | 0.78565 | RAD51B | -0.46145 | 0.79292 |
| FZD8 | 0.49531 | -1.22881 | C8orf76 | 0.67878 | -0.51364 |
| RPS24 | 0.36570 | -0.32461 | RNF169 | 0.93353 | -0.49784 |
| ZCCHC24 | 0.70549 | -0.15001 | ZNF623 | 0.37708 | -0.30733 |
| CREBL2 | 0.70403 | 0.75410 | FOXO4 | 0.91309 | -1.33475 |
| IGFBP6 | 0.65272 | -0.33402 | COL4A5 | 1.92623 | -0.71756 |
| TMEM116 | 0.45492 | 0.54353 | COPB1 | 1.58772 | -0.14057 |
| AACS | 0.33592 | -0.23272 | PRDM11 | 1.49878 | -1.33591 |
| MMP14 | 0.42581 | 1.16524 | RTN4RL2 | 1.32121 | 2.47549 |
| INPP4B | 1.28208 | 0.93093 | SAMD4A | -0.53509 | -0.44738 |
| ERG28 | 0.73908 | 2.84068 | NAA40 | 1.33154 | 0.72015 |
| DBF4B | 0.48301 | 0.60448 | ZPR1 | 0.90420 | -0.36308 |
| TRIM25 | 0.35168 | -1.68752 | HYLS1 | 0.47356 | 0.61262 |
| EXOC7 | 0.54074 | -0.17137 | SLC38A10 | 0.56625 | -0.25582 |
| MEX3D | 0.34956 | 1.15560 | CUL2 | 0.54684 | 0.27464 |
| PDE4A | 0.85623 | 1.14276 | CUEDC2 | 1.24929 | 0.56354 |
| SLC27A1 | 0.21989 | 1.49021 | ATN1 | 1.40976 | -0.79027 |
| TRPM4 | 1.06568 | 1.10250 | GALNT6 | 0.63555 | 0.59446 |
| NDUFA3 | 0.49998 | 0.67745 | PXN | 0.88369 | -0.34845 |
| ZNF71 | 0.82254 | -0.23753 | PSMA6 | 0.89933 | 0.41213 |
| MZF1 | 0.52963 | -1.13092 | RALGAPA1 | 1.11388 | -0.19749 |
| SNRPB | 0.71527 | 0.22350 | SUSD6 | 0.42461 | -1.11726 |
| KCNAB2 | 1.17884 | 1.05168 | COQ6 | 0.60733 | 1.34251 |
| RRBP1 | 0.95314 | -0.08982 | TENM4 | 0.69029 | -2.00685 |
| ENTPD6 | 1.65174 | -0.30658 | MMD | 0.53622 | 1.22847 |
| NOL4L | 3.04643 | 1.25804 | TNNT1 | 1.24929 | 0.56070 |
| ITCH | 1.66191 | 0.09933 | TBCD | 1.16027 | 1.08739 |
| EIF6 | 1.05602 | -0.49553 | CLN3 | 1.11388 | -0.60130 |
| SOGA1 | 1.47324 | -0.63582 | RPL36 | 0.69471 | -0.11776 |
| OSER1 | 0.62654 | -1.06519 | TNFSF9 | 0.66079 | -1.14467 |
| UBE2V1 | 0.84688 | -0.01897 | TRIM29 | 0.65926 | 0.14957 |
| NFATC2 | 1.22747 | 0.32230 | ZNF358 | 0.53559 | -0.33757 |
| ATP9A | 1.14520 | -0.27492 | ZNF490 | 0.96102 | -1.18342 |
| SALL4 | 1.89866 | 1.30504 | SPINT2 | 0.92127 | 0.44945 |
| NPEPL1 | 0.64701 | -0.21828 | AXL | 1.27693 | -0.25507 |
| GNAS | 1.42335 | -0.06312 | SAE1 | 1.06055 | 0.25161 |
| BACH1 | 1.07705 | 0.31944 | LMTK3 | 1.12428 | 1.16426 |
| KDM2A | 0.98233 | -0.35325 | PPFIA3 | 0.62285 | 0.30337 |
| ETS2 | 0.94762 | -0.80769 | CPT1C | 1.09463 | 1.56856 |
| AGPAT3 | 1.44040 | 0.10183 | WWTR1 | -0.54930 | -0.42617 |
| COL6A1 | 0.86745 | 0.81907 | SMCHD1 | 1.44725 | -0.03974 |
| PISD | 1.14979 | 0.45915 | RIOK3 | 0.62761 | -1.17556 |
| KPNB1 | 1.43629 | -0.08311 | LMNA | 0.68503 | -0.22242 |
| HSPG2 | 1.46699 | 1.19809 | PABPC4 | 2.84402 | 0.23621 |
| SERINC2 | 0.84953 | 1.06709 | ST3GAL3 | 1.33531 | 1.13365 |
| ZMYM1 | 0.48301 | 0.56660 |  |  |  |
