# Supplementary figures and images for: Co-transcriptional R-loops-mediated epigenetic regulation drives growth retardation and docetaxel chemosensitivity enhancement in advanced prostate cancer
Source: Mol Cancer. 2024 Apr 24;23:79. doi: 10.1186/s12943-024-01994-0 (PMC11041046; doi:10.1186/s12943-024-01994-0)

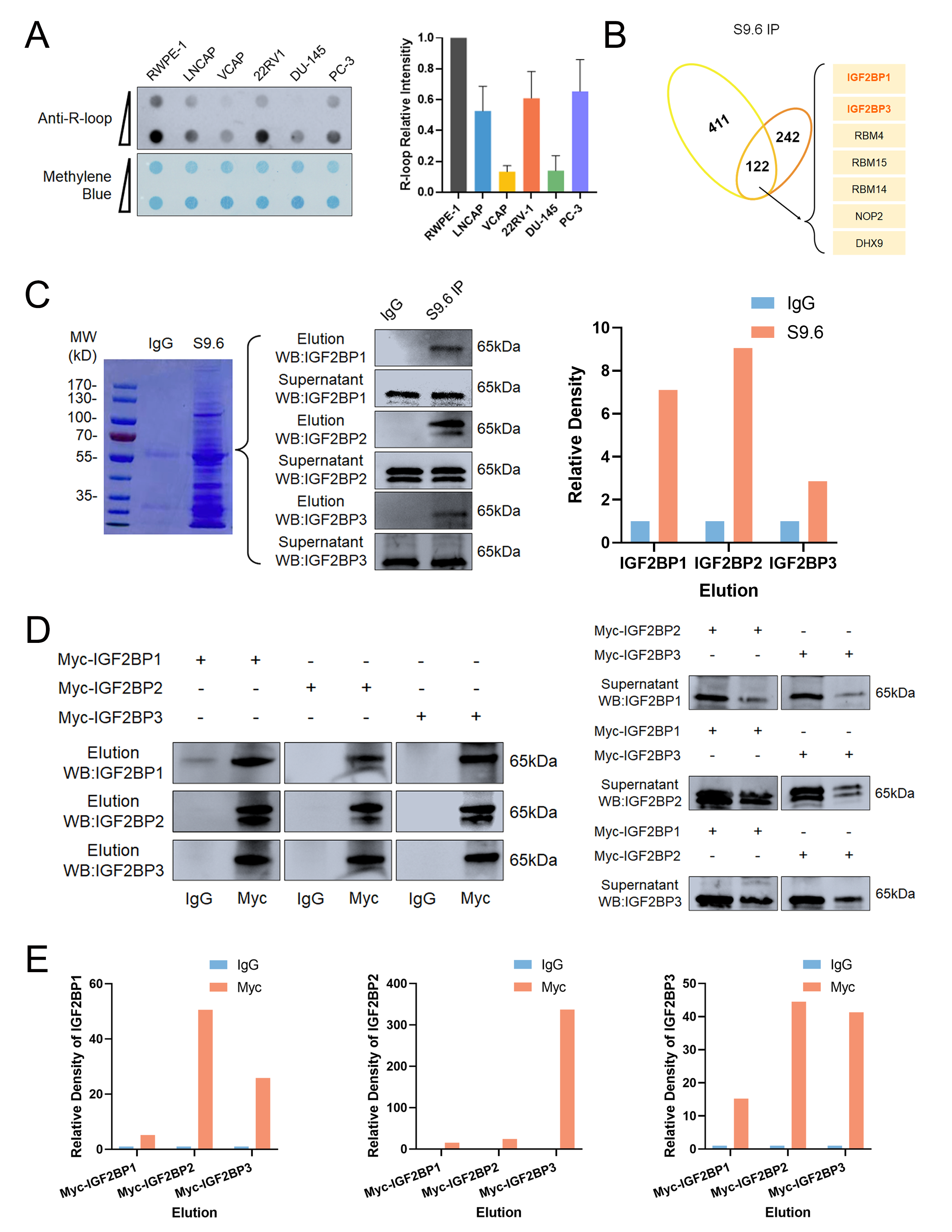

Supplement: Supplementary file 4 — Additional file 4: Supplementary Fig. 1. (A). Isolated R-loops from RPWE-1 and other prostate cancer cell lines (LNCAP, VCAP, 22RV1, DU-145, PC-3) were analyzed by Dot-blot. (Left) representative dot-blot results; (Right): quantification of dot blot. (B). R-loop regulators were identified by LC-MS/MS analysis using S9.6 antibody in two previously published studies(Yellow: RNA/DNA Hybrid Interactome Identifies DXH9 as a Molecular Player in Transcriptional Termination and R-Loop-Associated DNA Damage; Orange: Human proteins that interact with RNA/DNA hybrids). The list of genes included in the published S9.6 IP results were compared using Venn diagram. (C). Validation of S9.6 IP LC-MS/MS results by western blot assay. (Left): representative western blot results; (Right): quantification of western blot. (D). PC-3 cell lysates were immunoprecipitated with IgG or anti-Myc-tagged antibody. Precipitates were blotted for IGF2BP1/2/3. (E). Quantification of western blot. [file 12943_2024_1994_MOESM4_ESM.png]

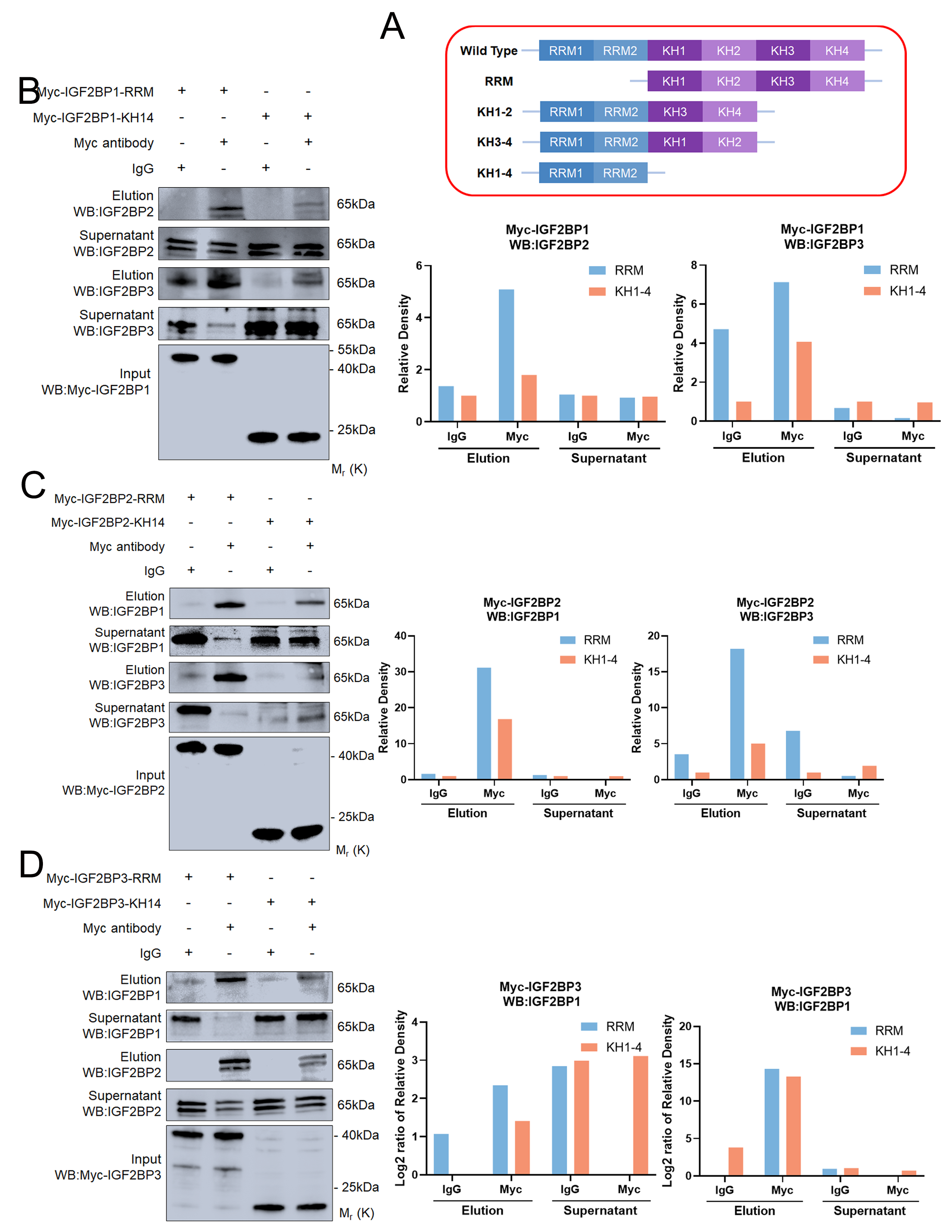

Supplement: Supplementary file 5 — Additional file 5: Supplementary Fig. 2. (A). Schematic diagram of the IGF2BPs fragments. (B-D). Interaction of individual IGF2BP protein fragments and other full length IGF2BP proteins detected with Co-IP. (Left): Representative western blot results. (Right): Quantification of western blot. [file 12943_2024_1994_MOESM5_ESM.png]

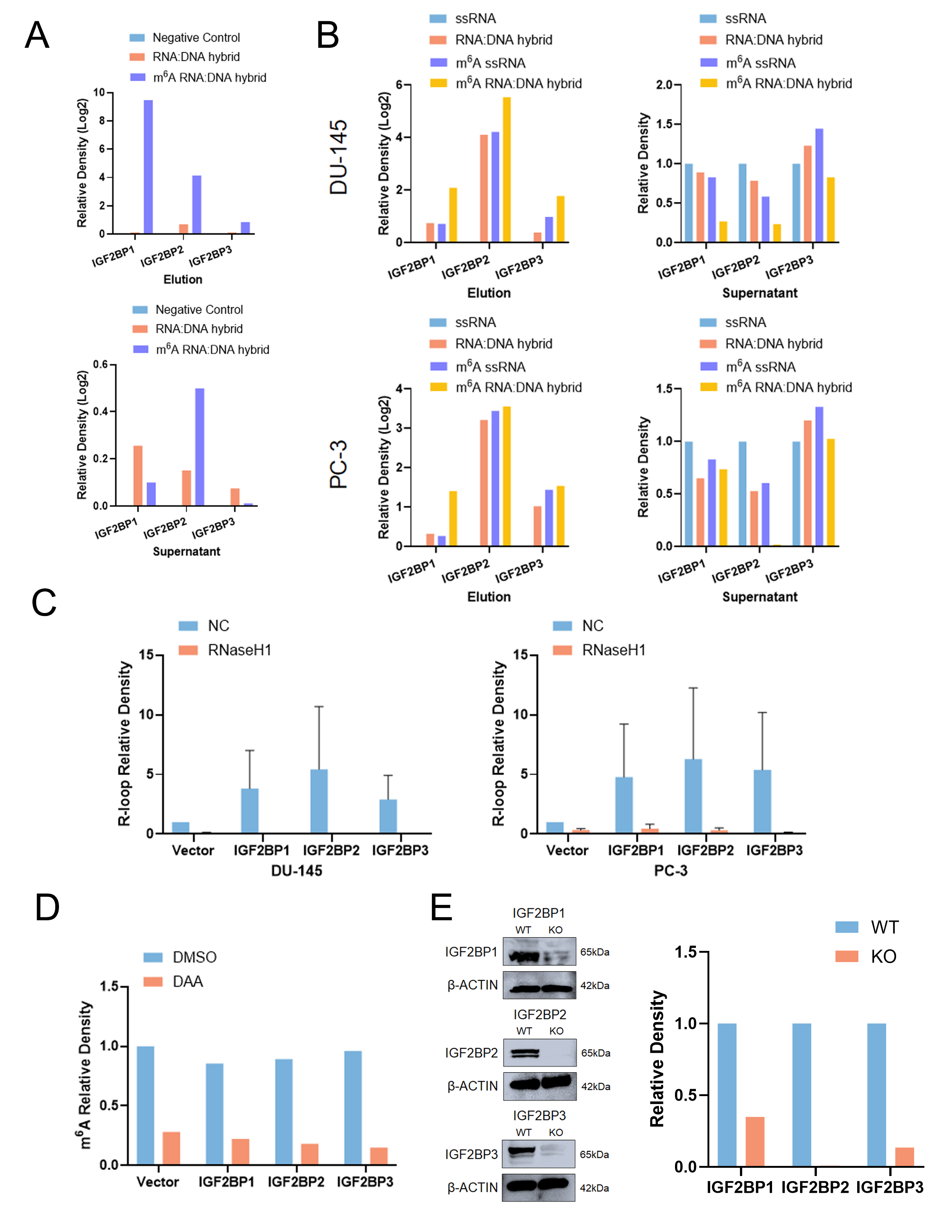

Supplement: Supplementary file 6 — Additional file 6: Supplementary Fig. 3. (A). Quantification of western blot results in Fig.2B. (B). Quantification of western blot results in Fig.2C. (C). Quantification of dot blot results in Fig.2F. Data are presented as means ± SD. (D). Quantification of dot blot results in Fig.2H. (E). CRISPR-Cas9 mediated KO of IGF2BP1/2/3 in PC-3 cells as detected by western blot. (Left): representative western blot results; (Right) :quantification of western blot. [file 12943_2024_1994_MOESM6_ESM.png]

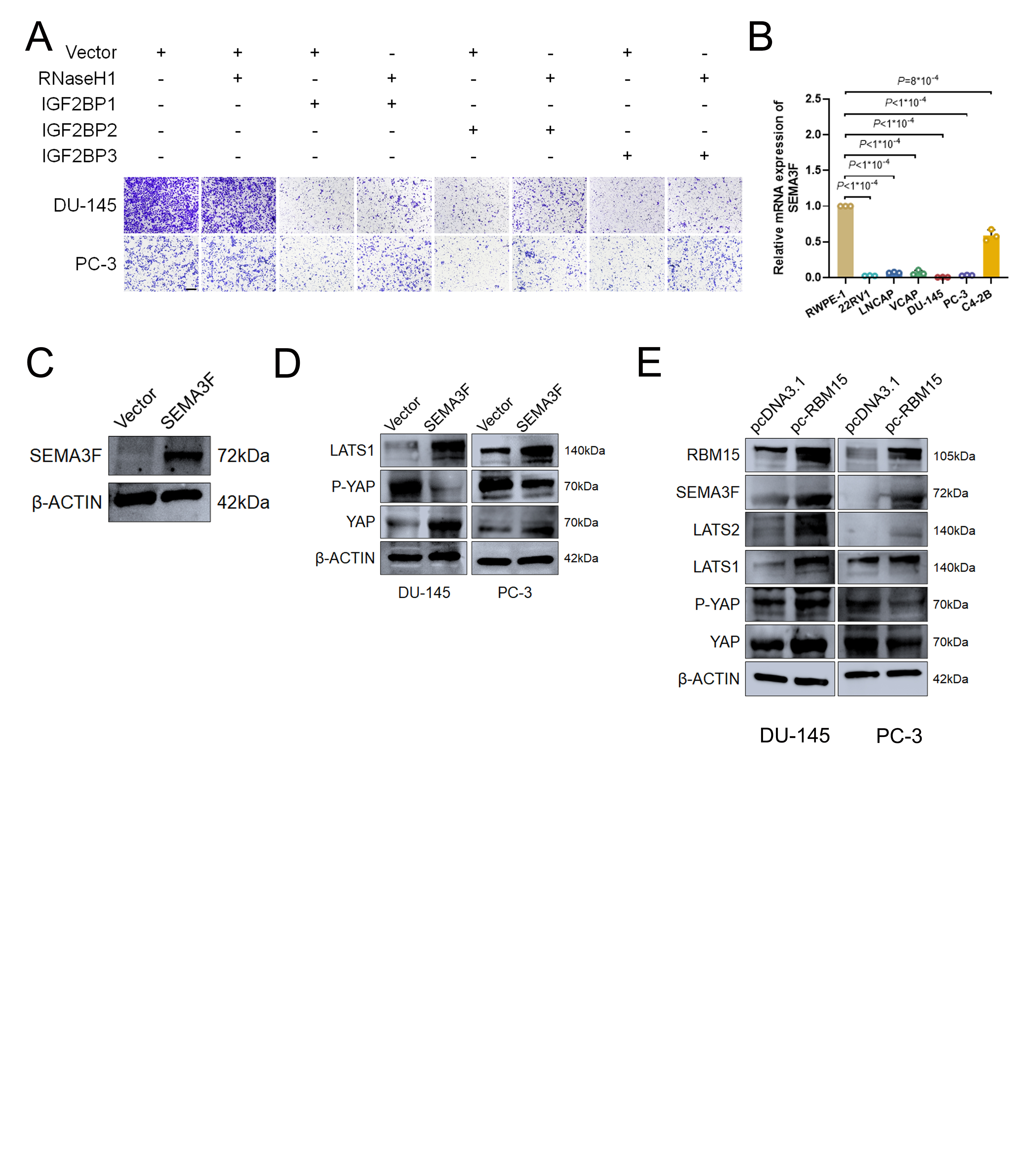

Supplement: Supplementary file 7 — Additional file 7: Supplementary Fig. 4. (A). IGF2BP1 KO PC-3 cells, as same as IGF2BP2 and IGF2BP3, were transfected with other full length IGF2BP proteins. The R-loop levels in each group were evaluated by dot-blot. (Upper): representative dot blot results; (Lower): quantification of dot blot. Data are presented as means ± SD, two-tailed unpaired t-test. (B). Quantification of western blot results in Fig.3C. *p-value < 0.05,**p-value < 0.01, ***p-value < 0.001,****p-value < 0.0001. [file 12943_2024_1994_MOESM7_ESM.jpg]

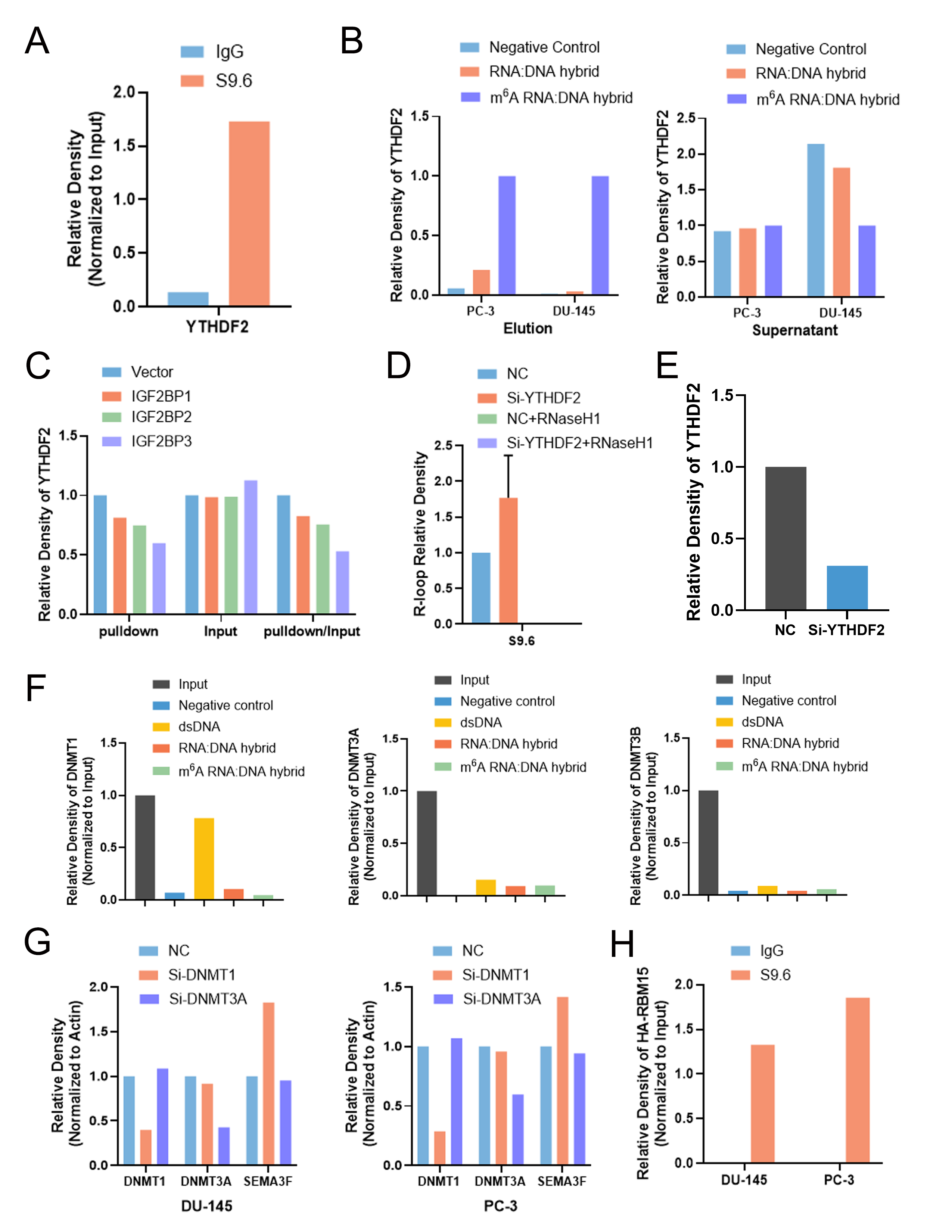

Supplement: Supplementary file 8 — Additional file 8: Supplementary Fig. 5. (A). Quantification of western blot results in Fig.4A. (B). Quantification of western blot results in Fig.4B. (C). Quantification of western blot results in Fig.4C. (D). Quantification of dot blot results in Fig.4D. Data are presented as means ± SD. (E). Quantification of western blot results in Fig.4D. (F). Quantification of western blot results in Fig.6D. (G). Quantification of western blot results in Fig.6E. (H). Quantification of western blot results in Fig.7A. [file 12943_2024_1994_MOESM8_ESM.png]

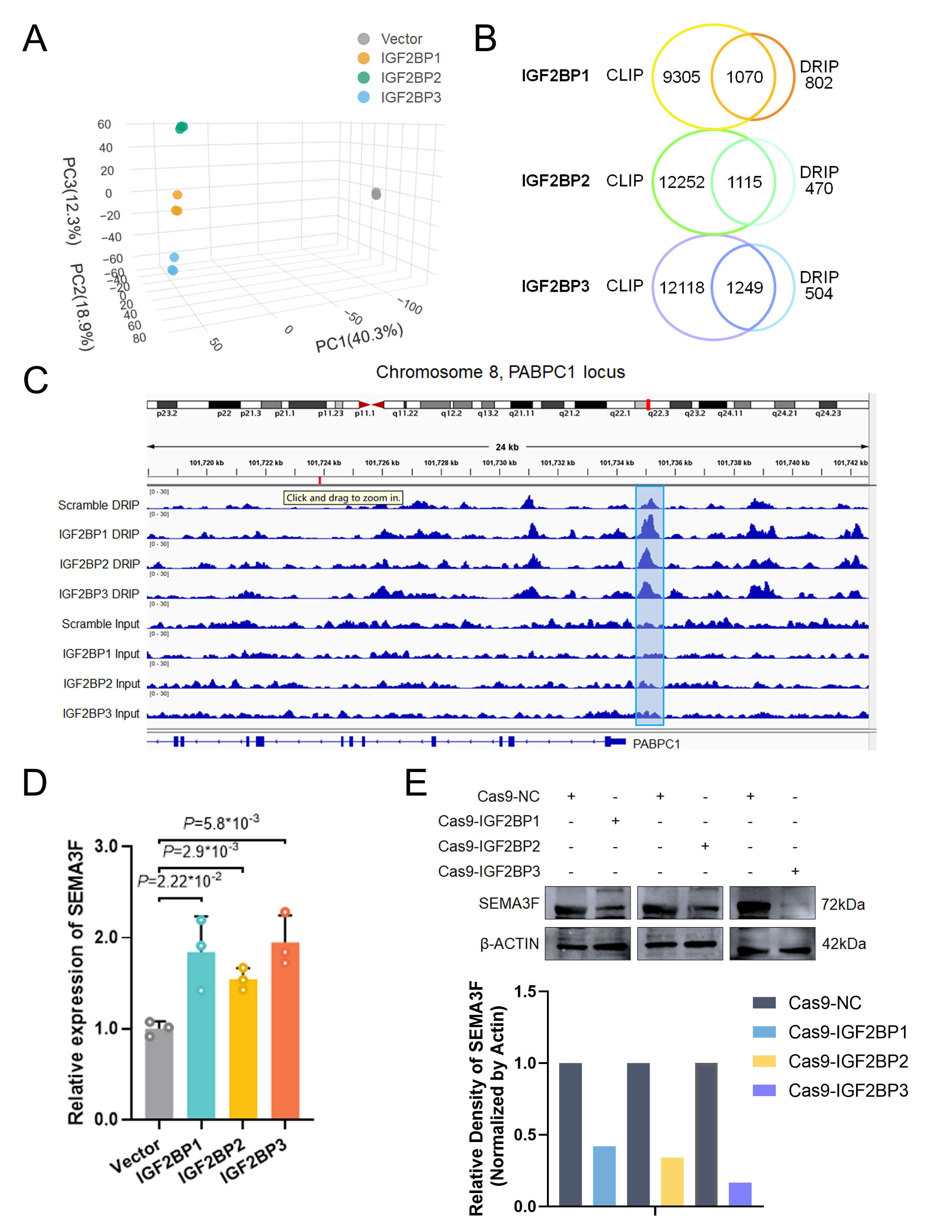

Supplement: Supplementary file 9 — Additional file 9: Supplementary Fig. 6. (A). Principal component analysis (PCA) constructed using the information from RNA-seq simultaneously. (B). The list of genes included in the DRIP-seq results and published PAR-CLIP-seq results were compared using Venn diagram. (C). The coverage plots of S9.6 DRIP densities in the promoter region of the indicated gene (PABPC1). (D). Relative expression of SEMA3F mRNA in IGF2BPs overexpression PC-3 cells compared to control by RT-qPCR. Data are presented as means ± SD, two-tailed unpaired t-test. (E). Effect of IGF2BPs KO on SEMA3F protein levels. (Upper): Representative western blot results; (Lower): Quantification of western blot. [file 12943_2024_1994_MOESM9_ESM.png]

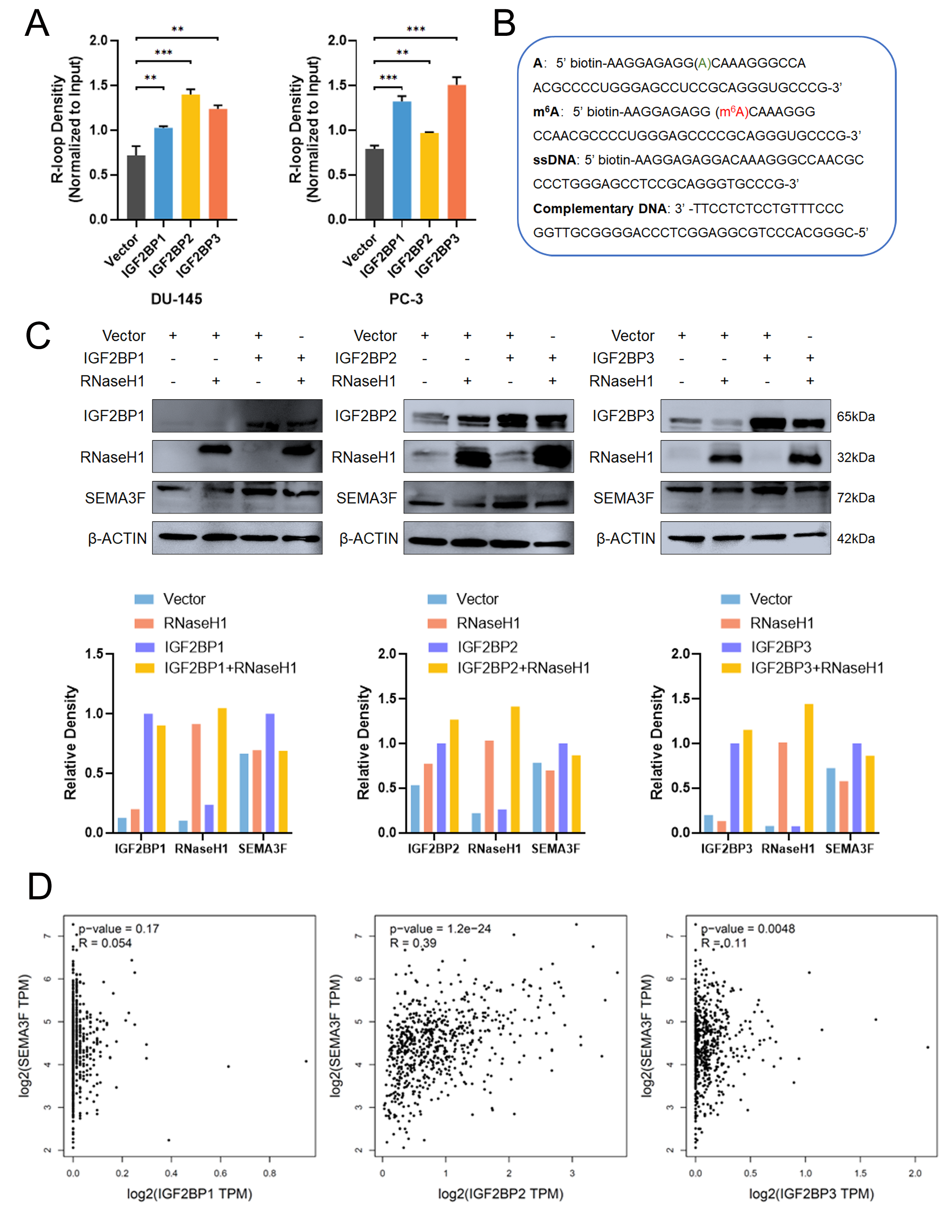

Supplement: Supplementary file 10 — Additional file 10: Supplementary Fig. 7. (A). Quantification of dot blot results in Fig.3B. Data are presented as means ± SD. (B). The ssDNA and RNA:DNA hybrid probes with methylated (red) or unmethylated (green) adenosine were shown. (C). Effect of RNaseH1 overexpression on endogenous IGF2BPs-induced SEMA3F upregulation in western blot assays. (Upper): representative western blot results; (Lower): quantification of western blot. (D). Correlation of mRNA expression between IGF2BPs and SEMA3F from GEPIA portal determined by Pearson coefficient. *p-value < 0.05,**p-value < 0.01, ***p-value < 0.001,****p-value < 0.0001. [file 12943_2024_1994_MOESM10_ESM.png]

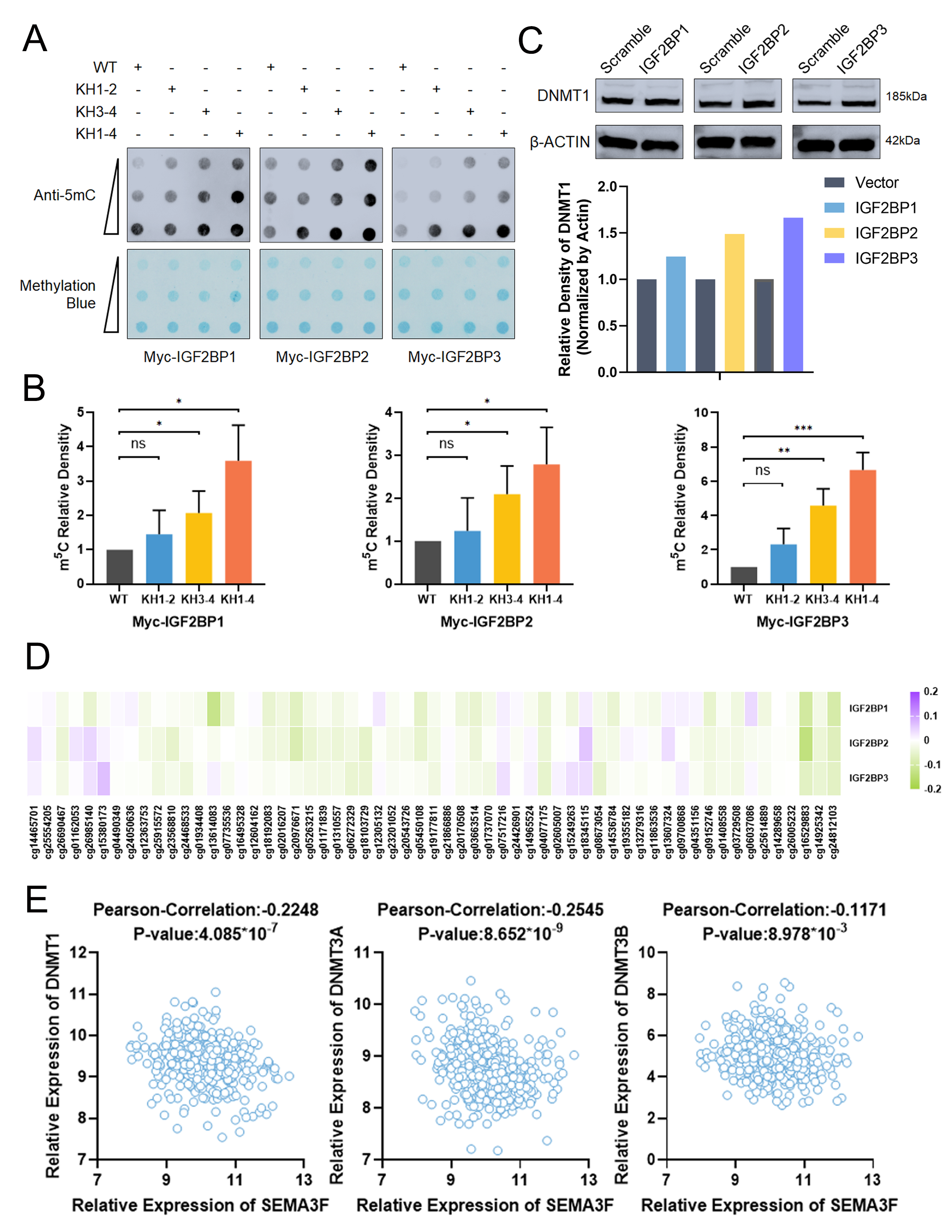

Supplement: Supplementary file 11 — Additional file 11: Supplementary Fig. 8. (A-B). Global DNA 5mC levels in IGF2BP-KO cells treated with wild-type or mutated IGF2BPs. A: Representative dot blot results are shown. B: Quantification of dot blot results. Data are presented as means ± SD, two-tailed unpaired t-test. (C). Effect of IGF2BPs overexpression on DNMT1 in western blot assays. (Upper): Representative western blot results; (Lower): Quantification of western blot. (D). Methylation level changes of CpG sites on the IGF2BPs co-targets promoter by IGF2BPs overexpression. (E). Correlation of mRNA expression between DNMTs and SEMA3F from GEPIA portal determined by Pearson coefficient.*p-value < 0.05, **p-value < 0.01, ***p-value < 0.001,****p-value < 0.0001. [file 12943_2024_1994_MOESM11_ESM.png]

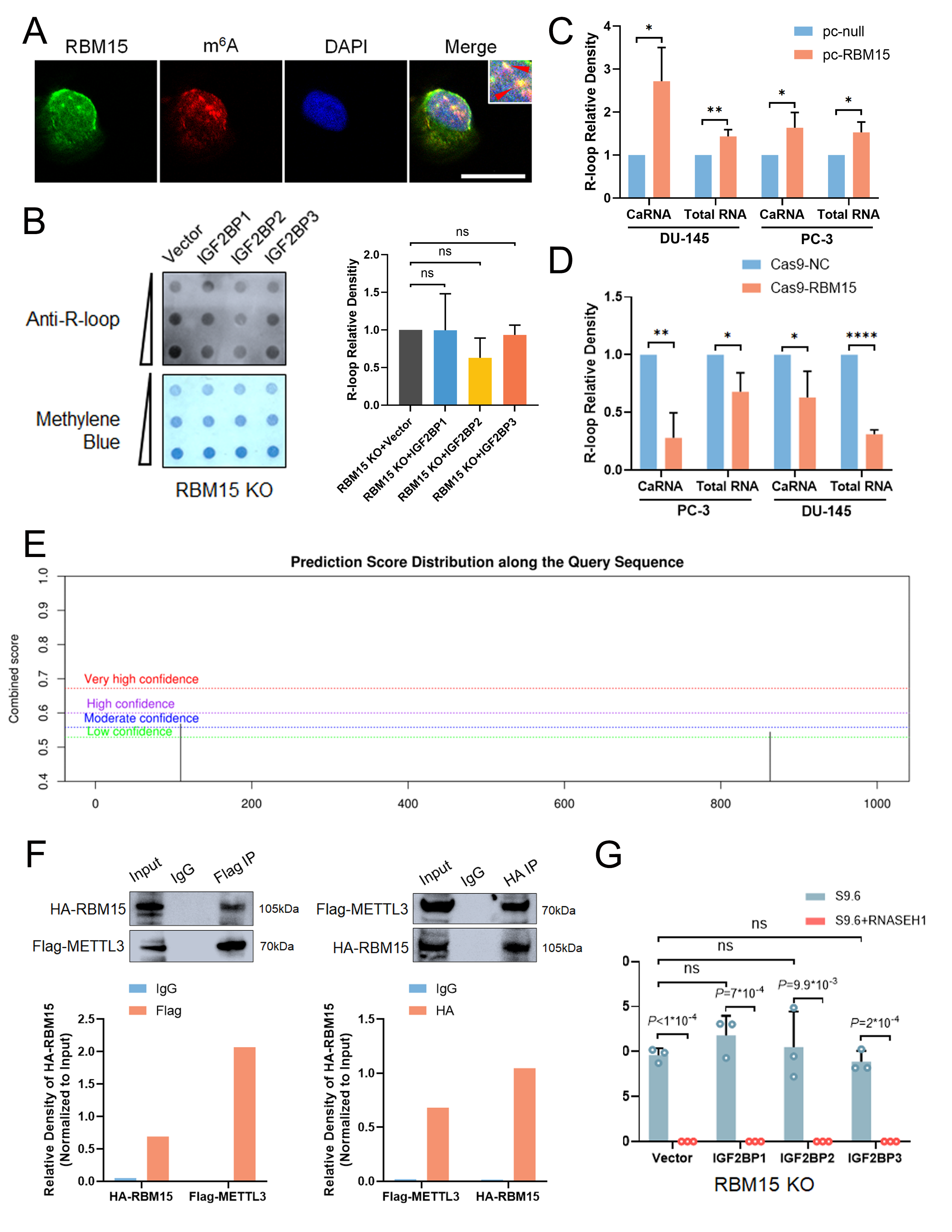

Supplement: Supplementary file 12 — Additional file 12: Supplementary Fig. 9. (A). PC-3 cells were immunostained for RBM15 and anti-m6A antibody; representative images are shown (scale bar: 20μm). (B). RBM15 KO PC-3 cells were transfected by Myc-IGF2BPs and vector, and the R-loop levels were assessed by dot-blot. (Left) representative dot-blot results; (Right): quantification of dot blot. Data are presented as means ± SD, two-tailed unpaired t-test. (C). Quantification of dot blot results in Fig.7F. Data are presented as means ± SD, two-tailed unpaired t-test. (D). Quantification of dot blot results in Fig.7H. Data are presented as means ± SD, two-tailed unpaired t-test. (E). Bioinformatics site SRAMP predicts m6A methylation sites in the sequence of SEMA3F promoter. (F). 293T purified Flag-METTL3 protein was mixed with 293T purified HA-RBM15 protein. The mixture was immunoprecipitated with IgG, anti-Flag-tagged and anti-HA-tagged antibody. Precipitates were blotted for Flag-purified METTL3 and HA-purified RBM15. (Upper): Representative western blot results. (Lower): Quantification of western blot assay. (G). R-loop levels of SEMA3F promoter in IGF2BPs overexpression RBM15-KO PC-3 cells compared to control by DRIP-qPCR. RNaseH1 treated samples were used as negative control. Data are presented as means ± SD, two-tailed unpaired t-test. *p-value < 0.05, **p-value < 0.01,***p-value < 0.001, ****p-value < 0.0001. [file 12943_2024_1994_MOESM12_ESM.png]

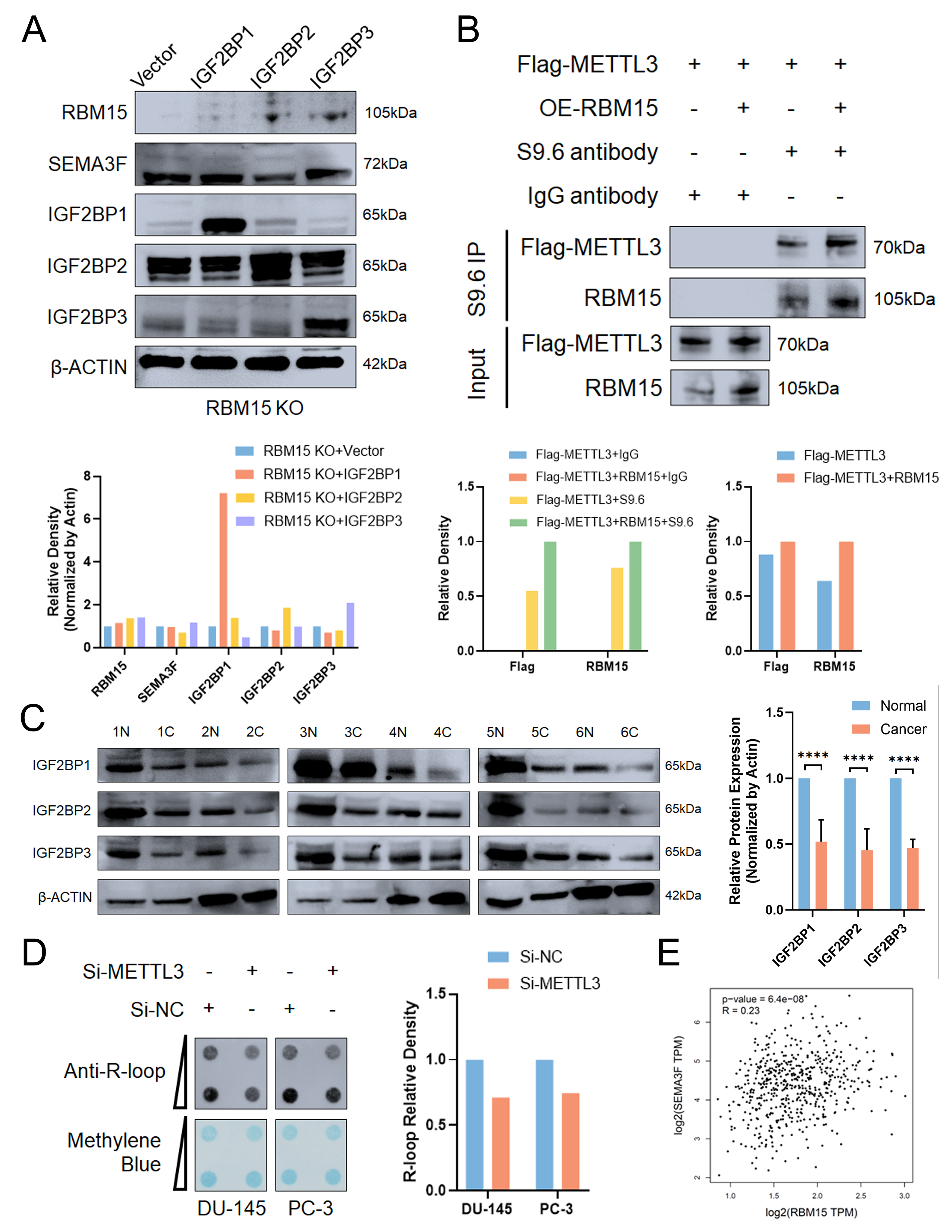

Supplement: Supplementary file 13 — Additional file 13: Supplementary Fig. 10. (A). RBM15 KO PC-3 cells were transfected by Myc-IGF2BPs and vector, and the expression of SEMA3F was evaluated by western blot. (Upper): Representative western blot results; (Lower): Quantification of western blot. (B). Identification of R-loop binding proteins by S9.6 IP and western blot assay. Experiments were performed with Flag-purified METTL3 protein, IgG antibody, RBM15 antibody and S9.6 antibody in PC-3 cell lysates. (Upper): Representative western blot results. (Lower): Quantification of western blot assay. (C). IGF2BP proteins were determined in PCa samples and paired adjacent normal tissues (n=6). (Left): Representative western blot results. (Right): Quantification of western blot assay. Data are presented as means ± SD, two-tailed unpaired t-test. (D). PC-3 cells were transfected with negative control and METTL3 siRNA. The R-loop levels were assessed by dot-blot. (Left): Representative dot blot results. (Right): Quantification of dot blot assay. Data are presented as means ± SD. (E). Correlation of mRNA expression between RBM15 and SEMA3F from GEPIA portal determined by Pearson coefficient. *p-value < 0.05,**p-value < 0.01, ***p-value < 0.001,****p-value < 0.0001. [file 12943_2024_1994_MOESM13_ESM.png]

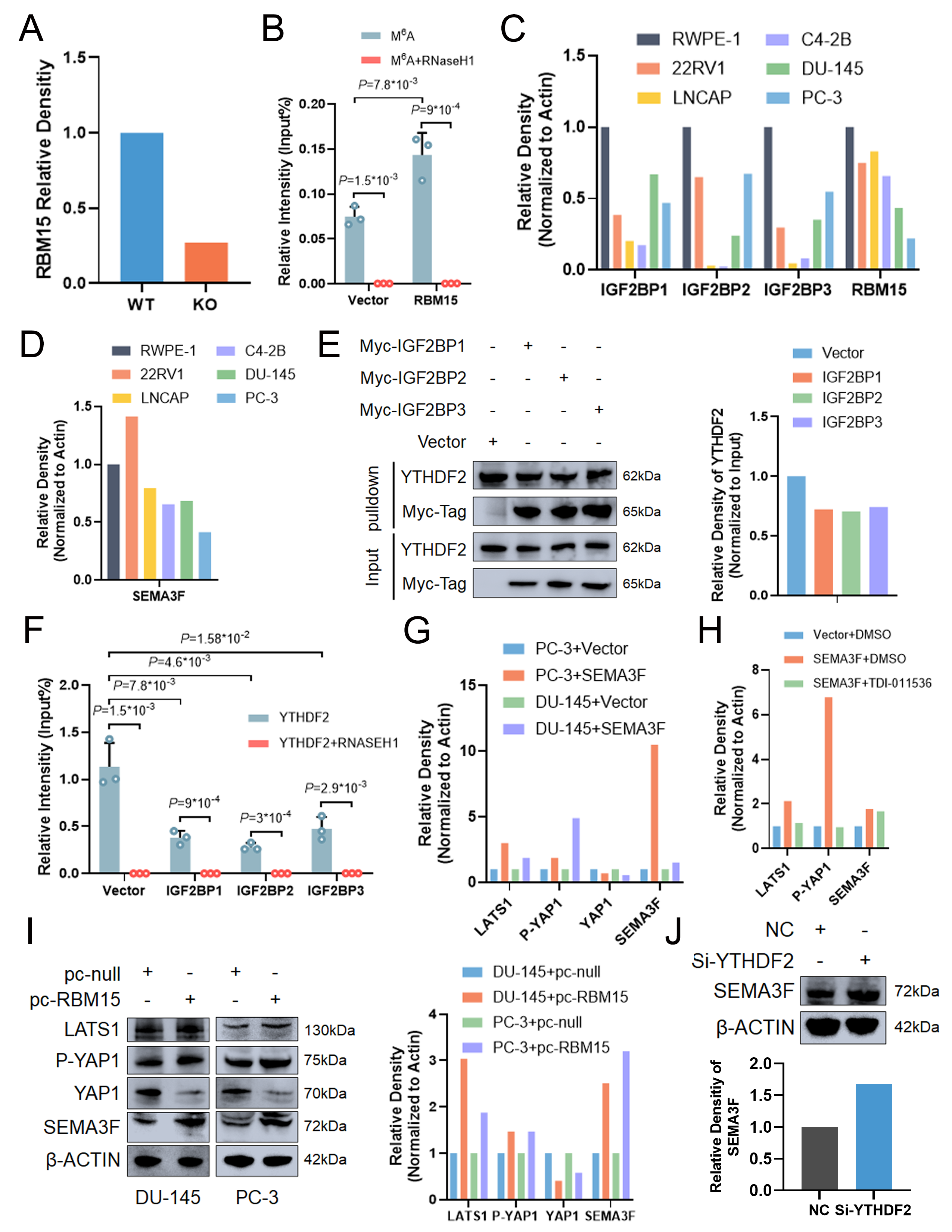

Supplement: Supplementary file 14 — Additional file 14: Supplementary Fig. 11. (A). Quantification of western blot results in Fig.7B. (B). Re-ChIP analysis in PC-3 cells with anti-RBM15 and anti-m6A antibodies. RNaseH1 treated samples were used as negative control. Data are presented as means ± SD, two-tailed unpaired t-test. (C). Quantification of western blot results in Fig.8C. (D). Quantification of western blot results in Fig.10D. (E). Pulldown followed by western blot indicated in vitro binding of m6A-modified RNA:DNA hybrid probe (using SEMA3F promoter sequence) with YTHDF2 in control and IGF2BPs overexpression PC-3 cells. (Left): Representative western blot results. (Right): Quantification of western blot assay. (F). The levels of SEMA3F promoter in IGF2BPs overexpression DU-145 and PC-3 cells compared to control by ChIP-qPCR (using YTHDF2 antibody and IgG antibody), Data are presented as means ± SD, two-tailed unpaired t-test. (G). Quantification of western blot results in Fig.10J. (H). Quantification of western blot results in Fig.10K. (I). Effect of RBM15 overexpression on Hippo pathway in DU-145 and PC-3 cells by western blot assay. (Left): Representative western blot results. (Right): Quantification of western blot assay. (J). Effect of YTHDF2 knockdown on SEMA3F protein levels in PC-3 cells by western blot assay. (Upper): Representative western blot results. (Lower): Quantification of western blot assay. [file 12943_2024_1994_MOESM14_ESM.png]

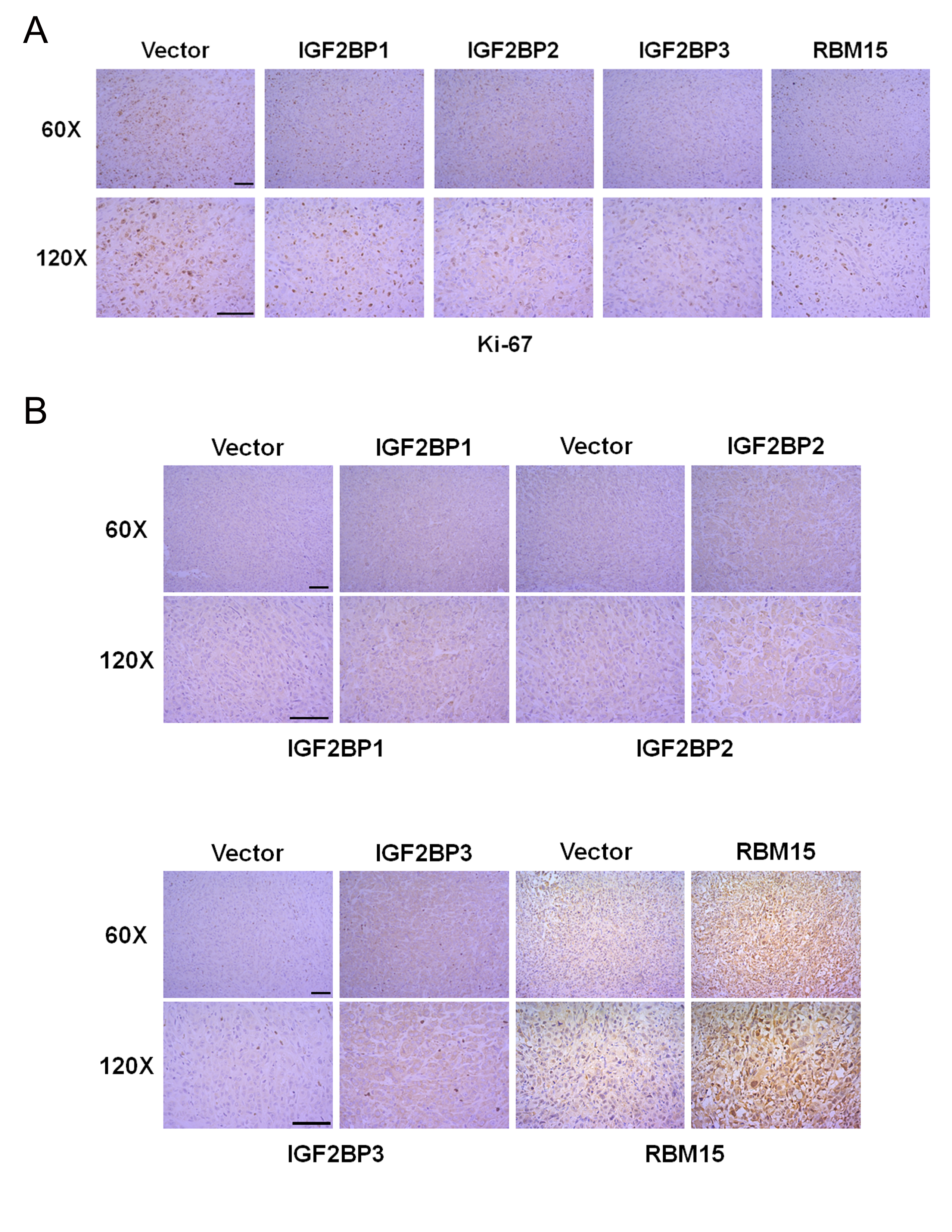

Supplement: Supplementary file 15 — Additional file 15: Supplementary Fig. 12. (A). Immunohistochemical labelling showed the downregulated expression of Ki-67 after RBM15 or IGF2BPs overexpression (A)(scale bar: 100μm). (B). Immunohistochemical labelling showed the overexpression of RBM15 and IGF2BPS after RBM15 or IGF2BPs overexpression (scale bar: 100μm). [file 12943_2024_1994_MOESM15_ESM.png]

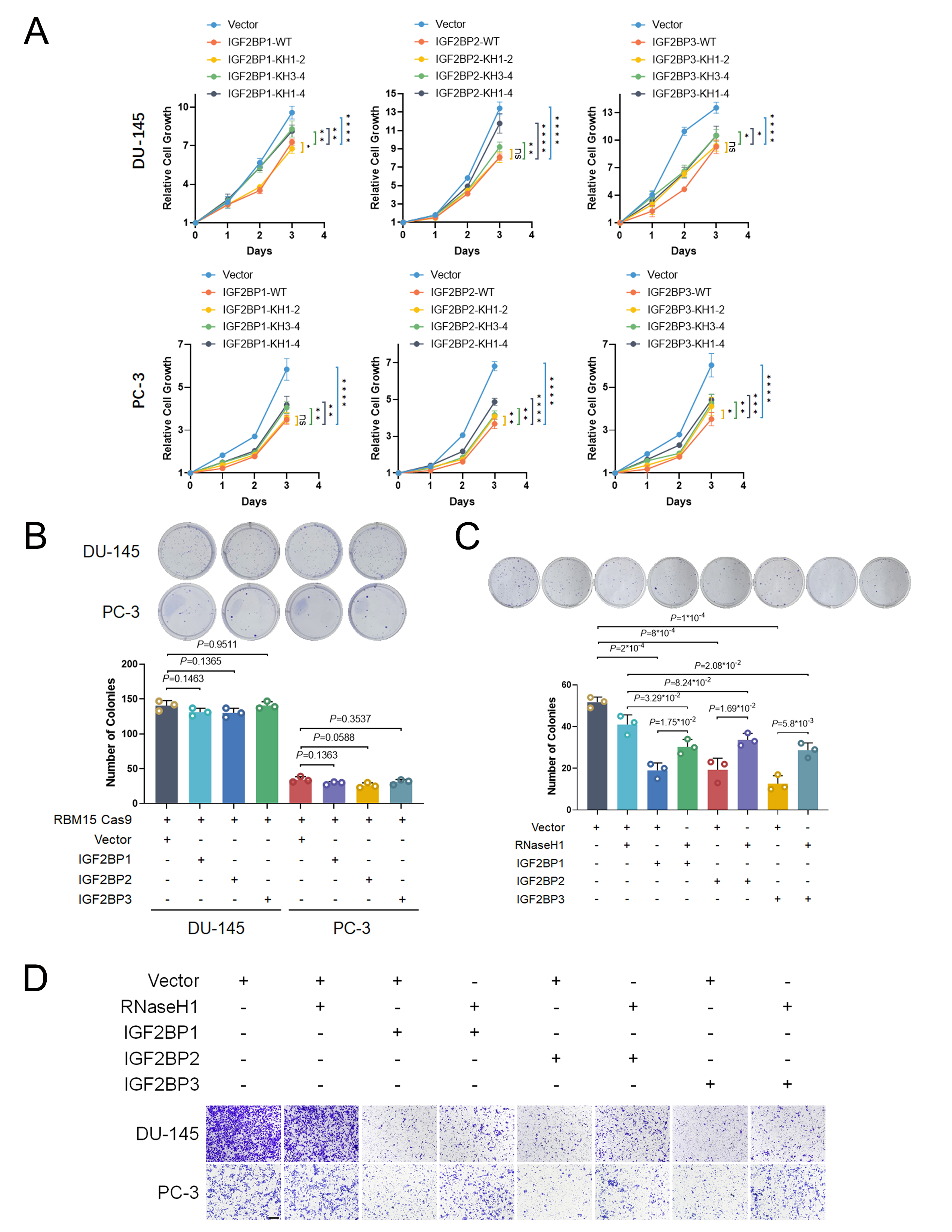

Supplement: Supplementary file 16 — Additional file 16: Supplementary Fig. 13. (A). CCK-8 assays revealed the cell proliferation abilities of IGF2BP-KO cells treated with wild-type or mutated IGF2BPs. Data are presented as means ± SD. (B). Clone formation capacities of IGF2BPs overexpression RBM15 KO PCa cells were assessed by the clone formation assay compared to control. Data are presented as means ± SD, two-tailed unpaired t-test. (C). Effect of RNaseH1 overexpression on endogenous IGF2BPs-induced inhibition of clone formation capacities in DU-145 cells. (D). Effect of RNaseH1 overexpression on endogenous IGF2BPs-induced inhibition of cell migration capacities in DU-145 and PC-3 cells. [file 12943_2024_1994_MOESM16_ESM.png]
