# Supplementary material for: Co-transcriptional R-loops-mediated epigenetic regulation drives growth retardation and docetaxel chemosensitivity enhancement in advanced prostate cancer
Source: Mol Cancer. 2024 Apr 24;23:79. doi: 10.1186/s12943-024-01994-0 (PMC11041046; doi:10.1186/s12943-024-01994-0)
Supplement: Supplementary file 17 — Additional file 17. [file 12943_2024_1994_MOESM17_ESM.pdf]

**Figure 1**

**Figure 1 A**

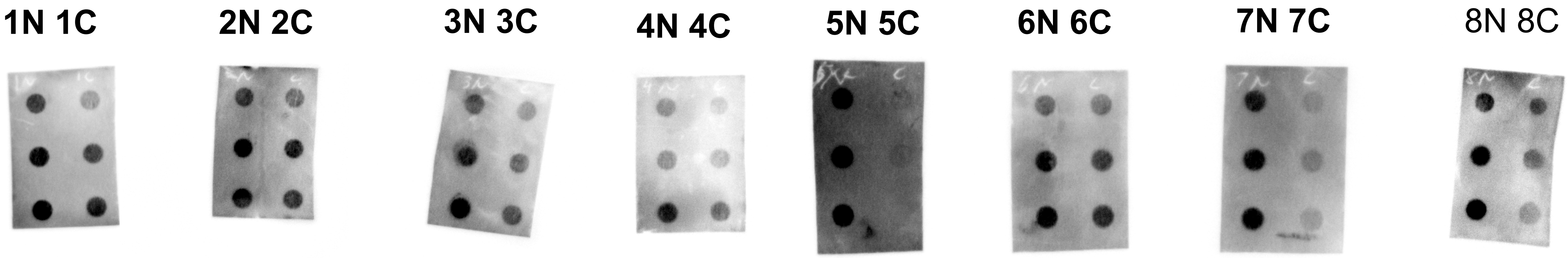


**Figure 1 C**

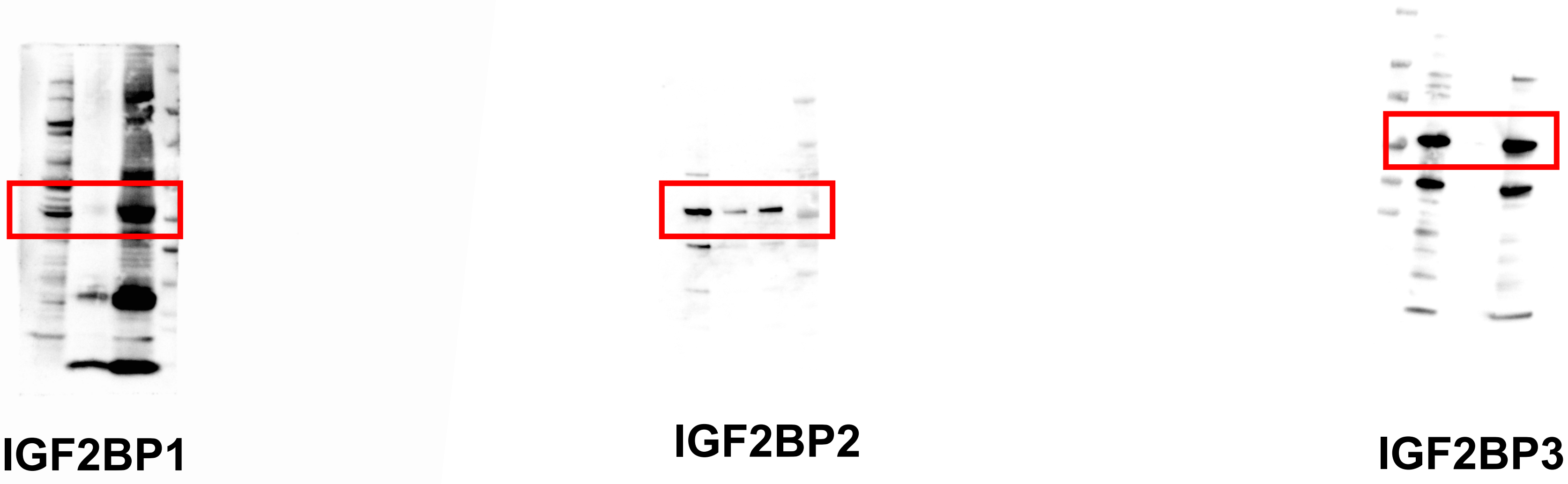

# Figure 2

## Figure 2 B

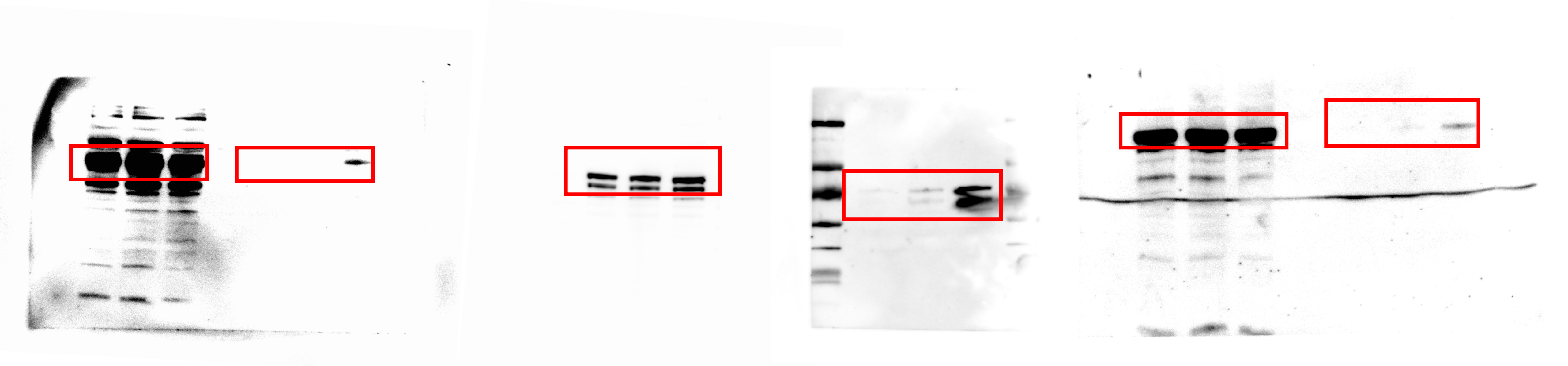

Supernatant: IGF2BP1 | Elution: IGF2BP1 | Supernatant: IGF2BP2 | Elution: IGF2BP2 | Supernatant: IGF2BP3 | Elution: IGF2BP3

Figure 2

Figure 2 C

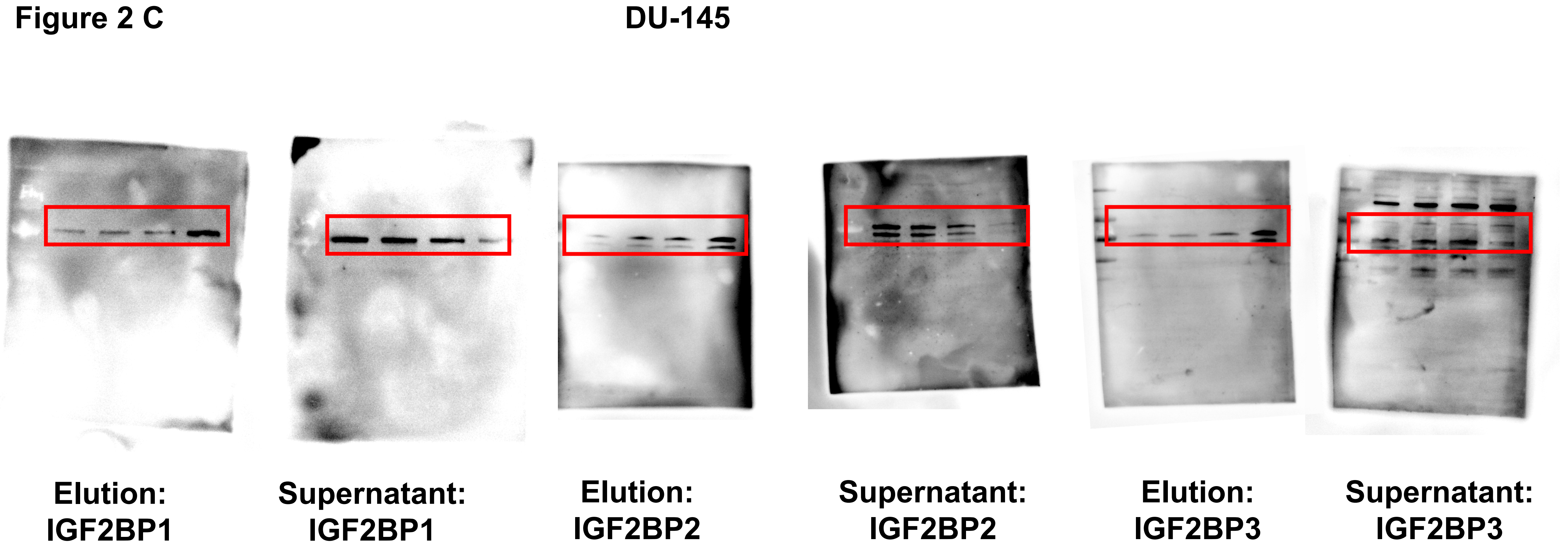

# Figure 2

## Figure 2 C

PC-3

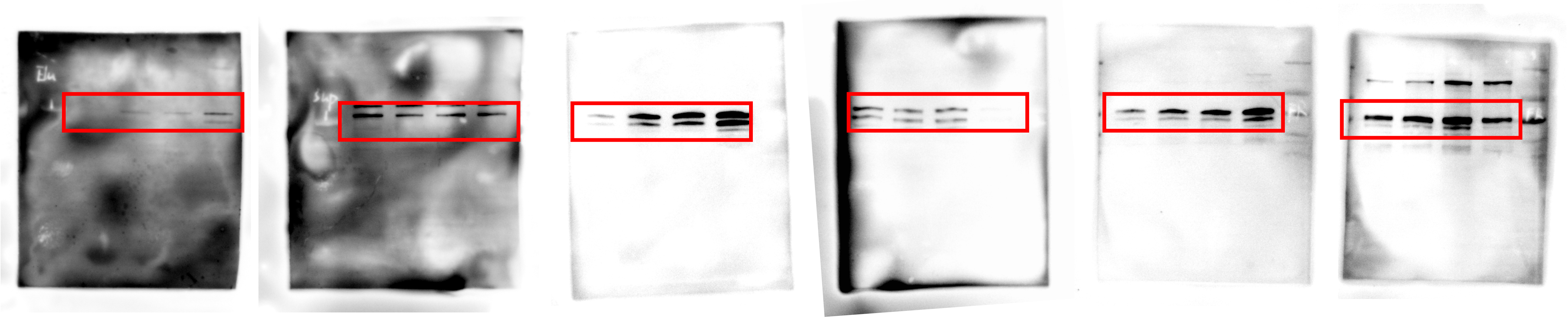

Elution: IGF2BP1 | Supernatant: IGF2BP1 | Elution: IGF2BP2 | Supernatant: IGF2BP2 | Elution: IGF2BP3 | Supernatant: IGF2BP3

## Figure 2

## Figure 2 F

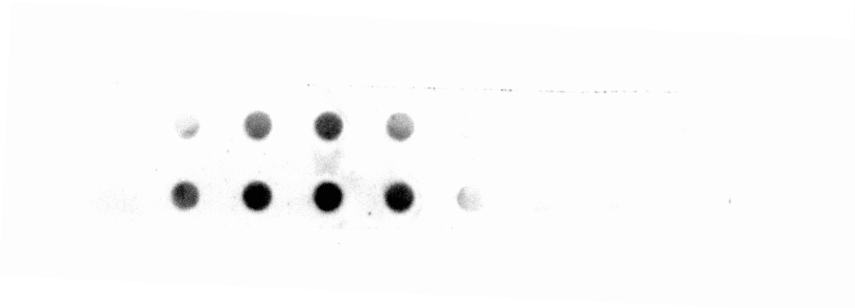

DU-145

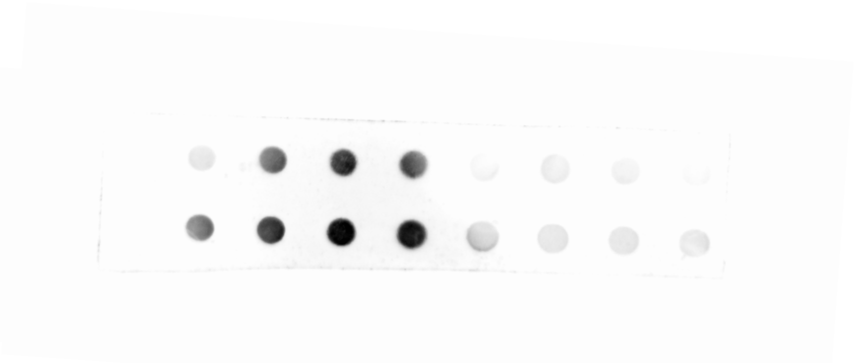

PC-3

## Figure 2 G

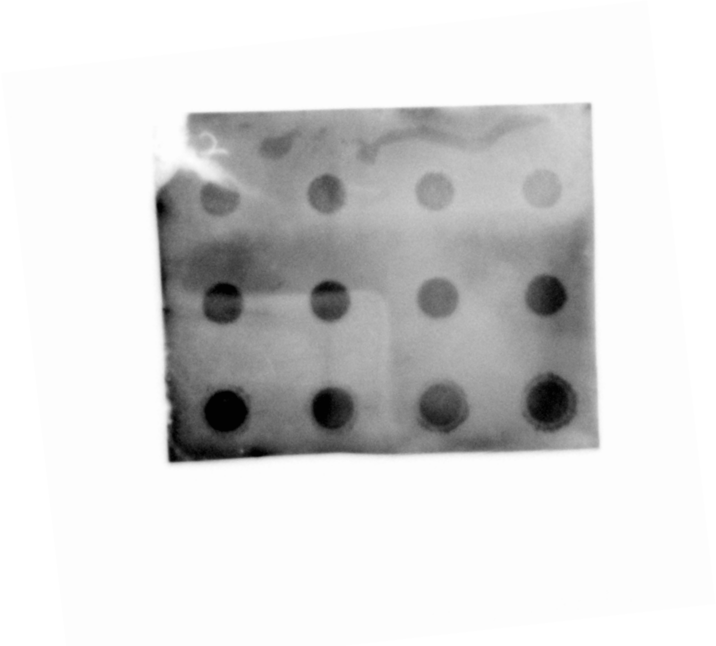

## Figure 2 H

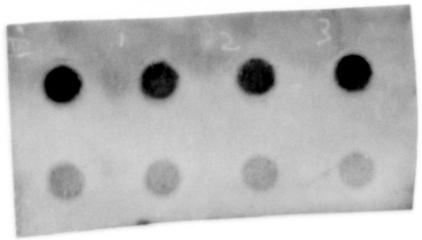

**Figure 3**

**Figure 3 A**

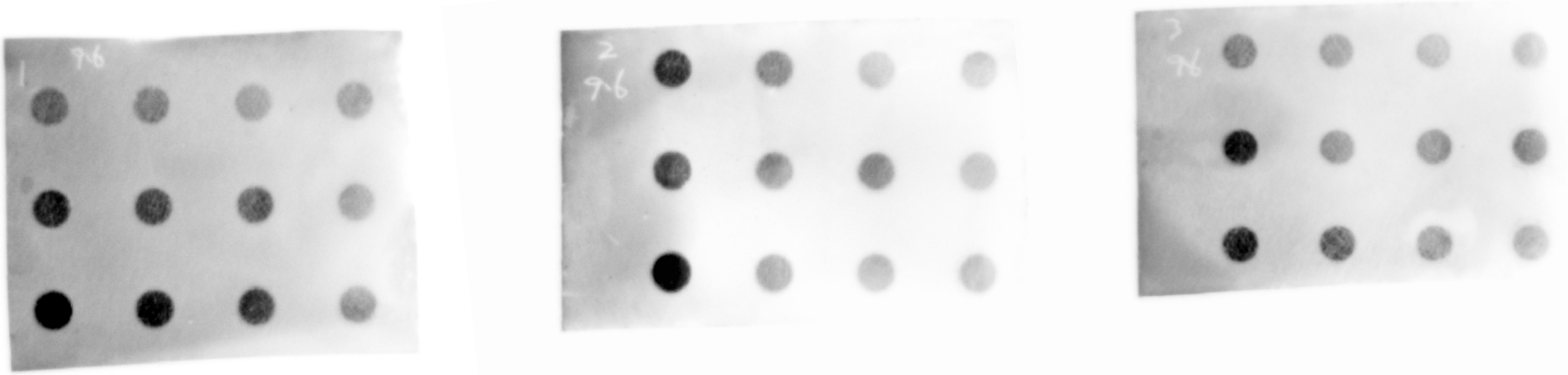

**IGF2BP1** **IGF2BP2** **IGF2BP3**

## Figure 3

### Figure 3C

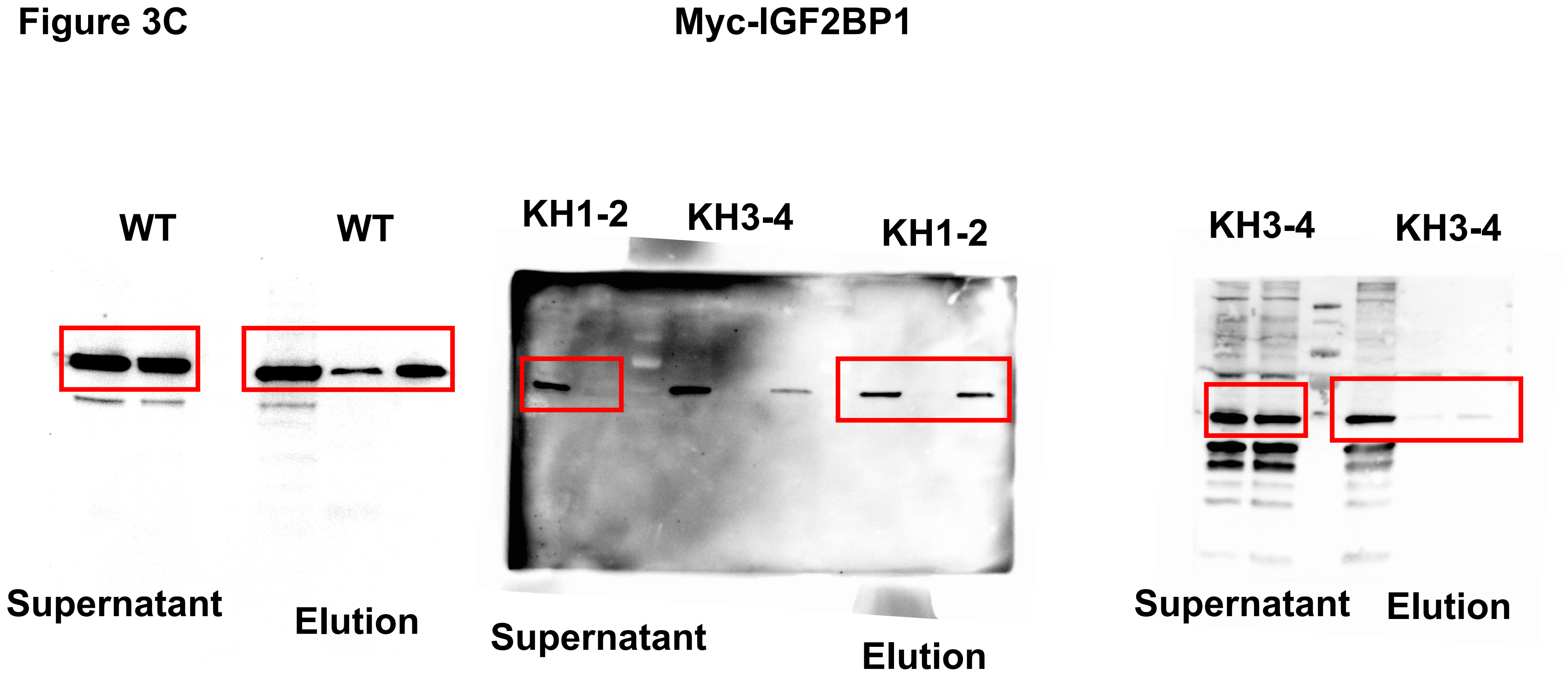

## Figure 3

### Figure 3 C

Myc-IGF2BP1

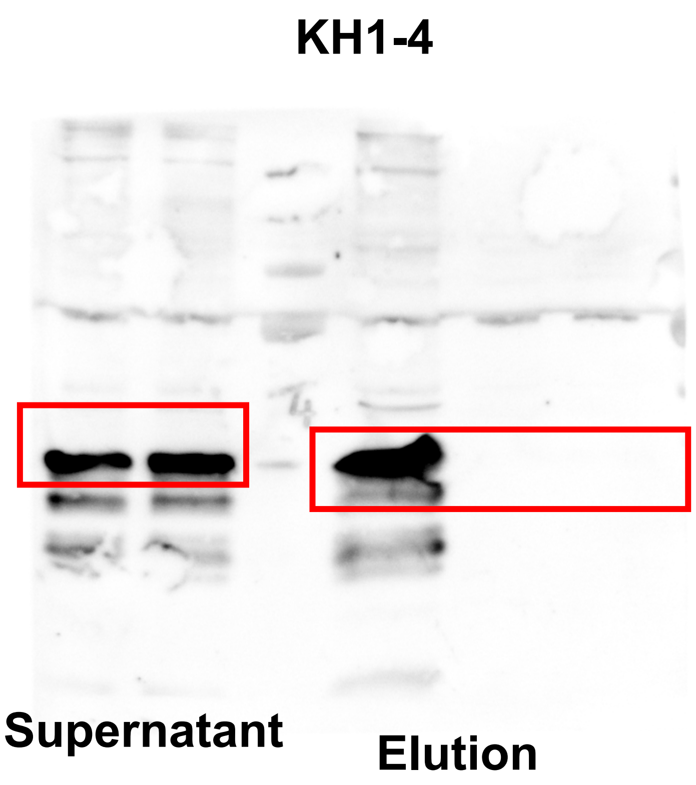


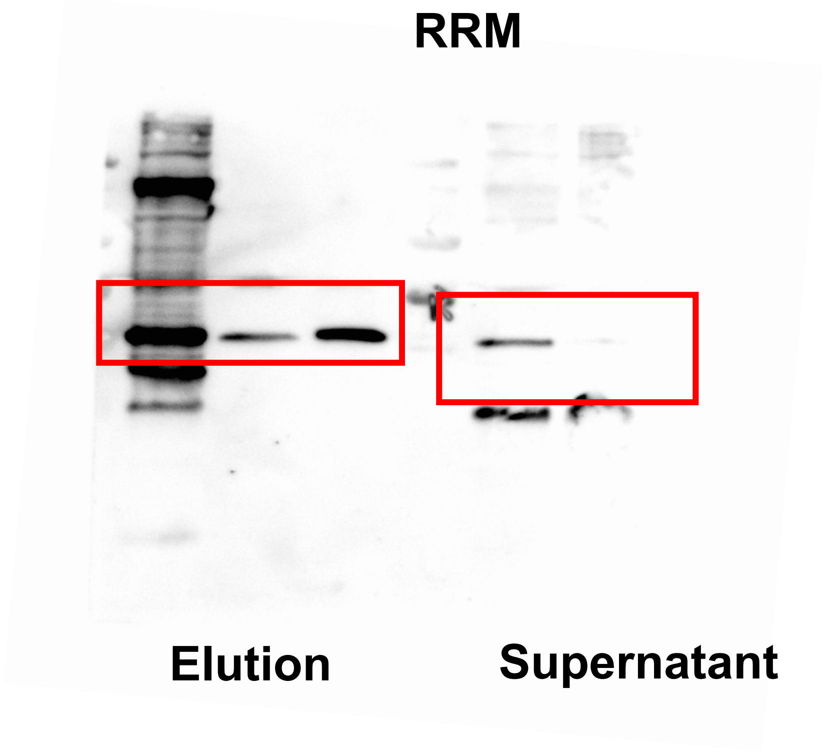

# Figure 3

## Figure 3 C

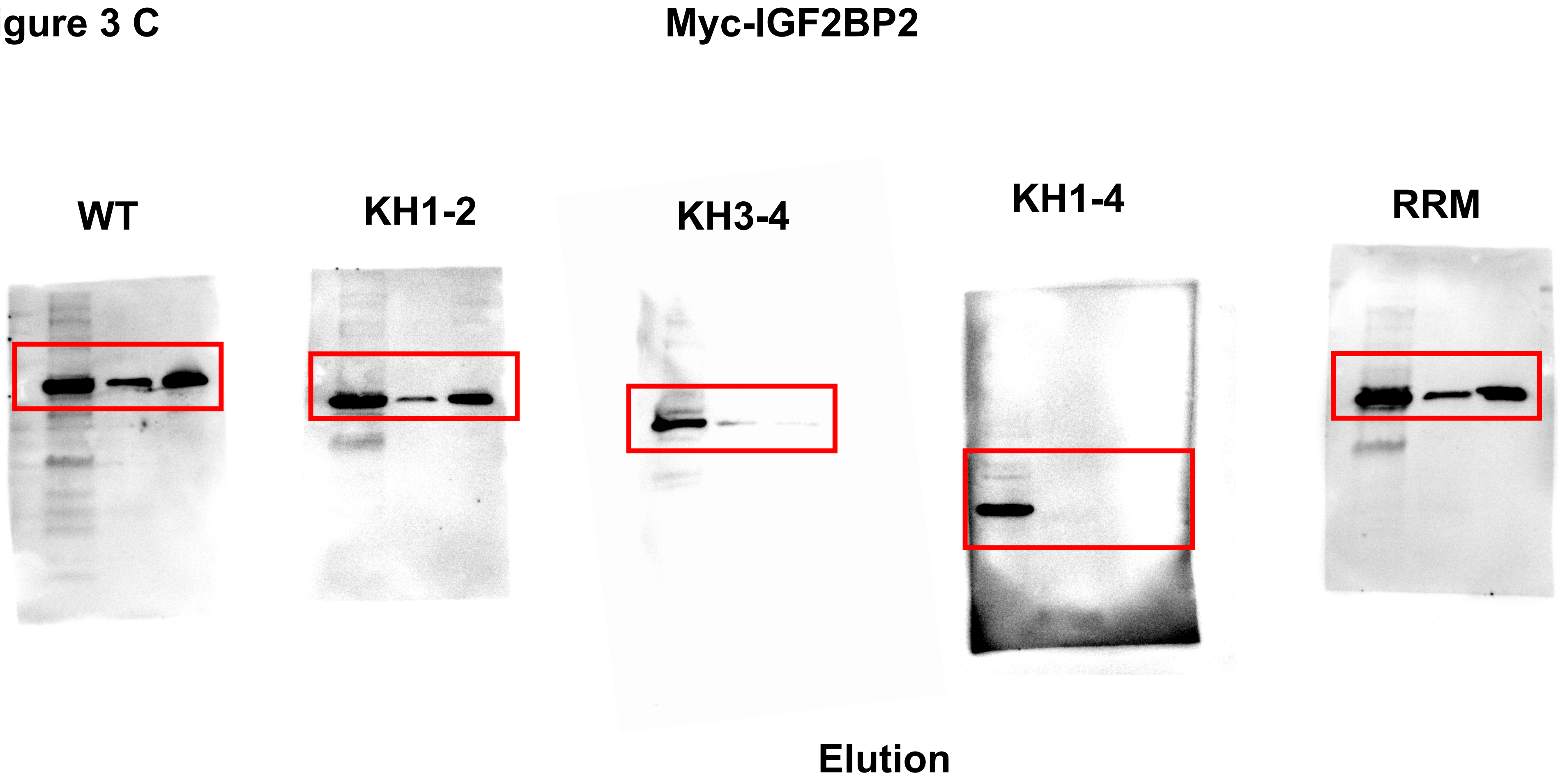

# Figure 3

## Figure 3 C

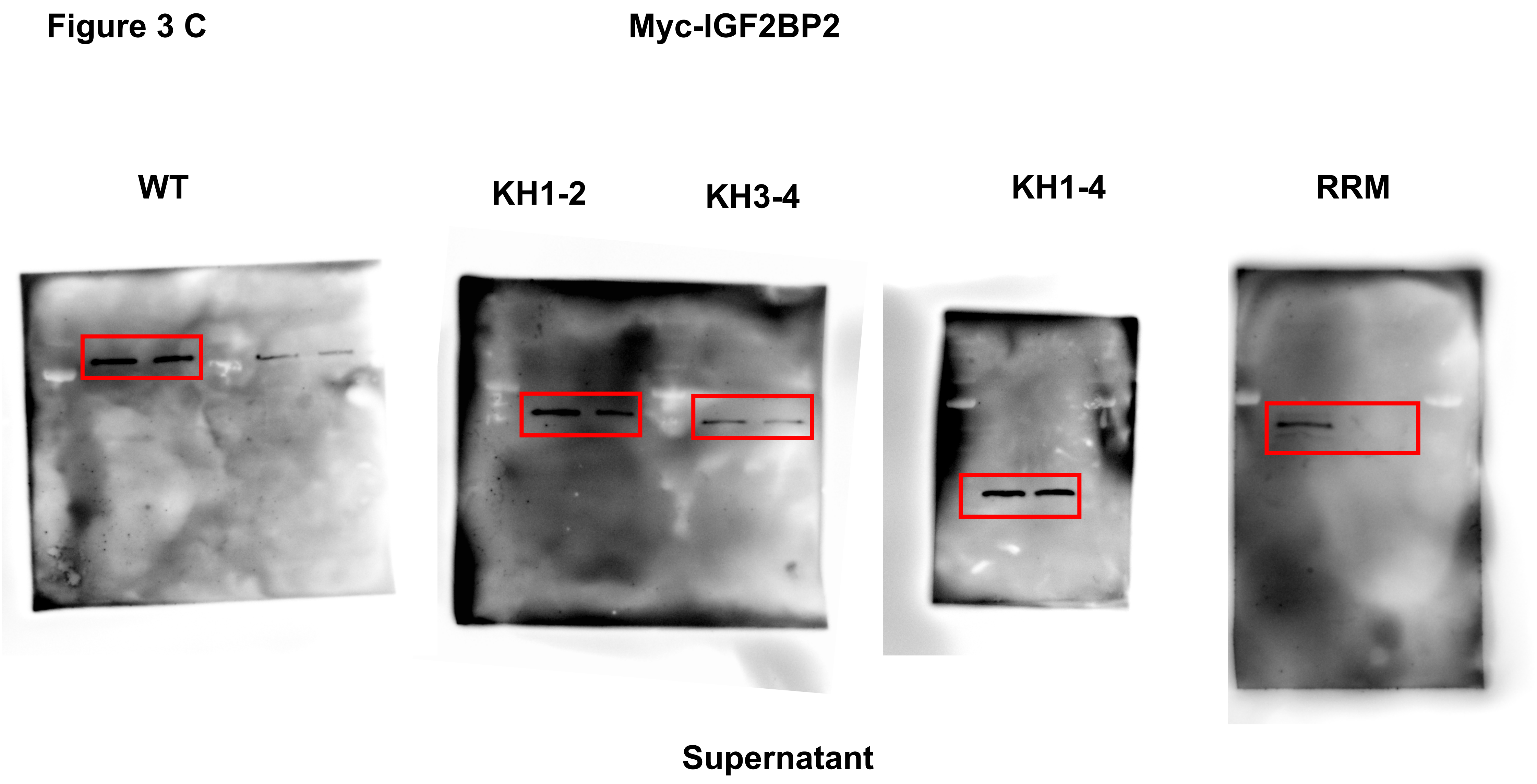

# Figure 3

## Figure 3 C

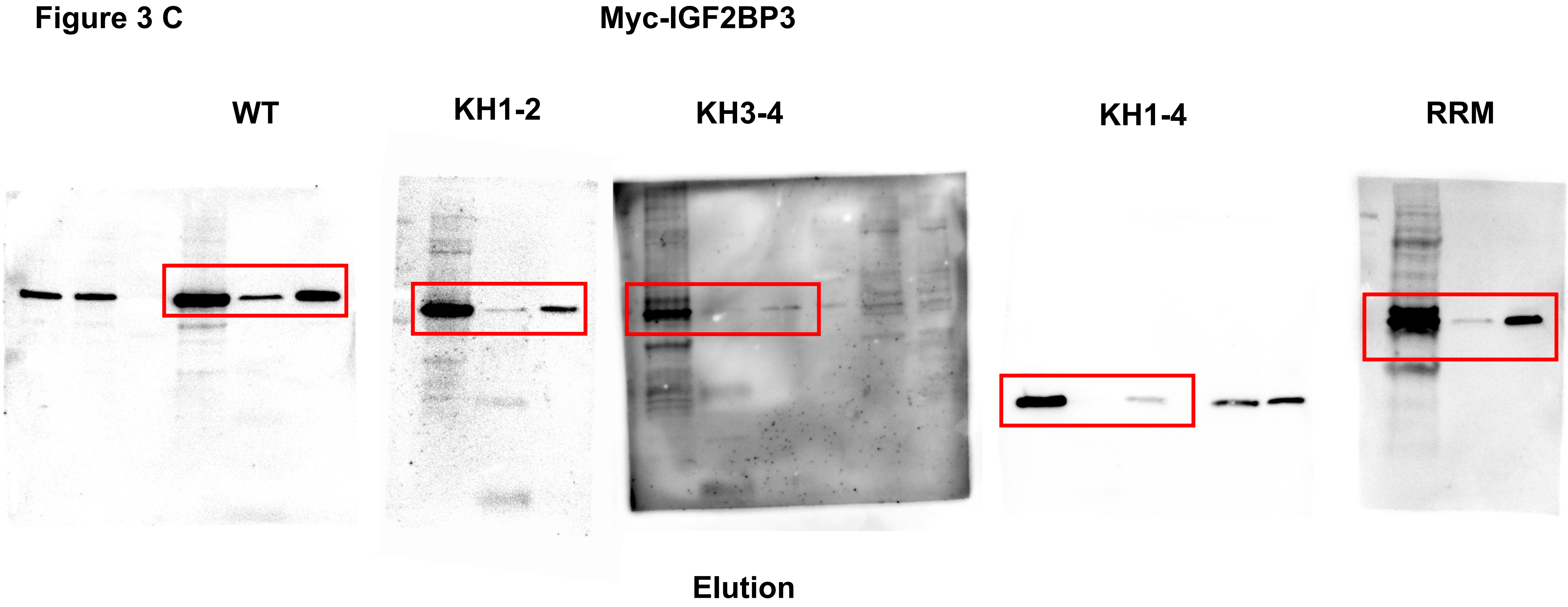

Figure 3

Figure 3 C

Myc-IGF2BP3

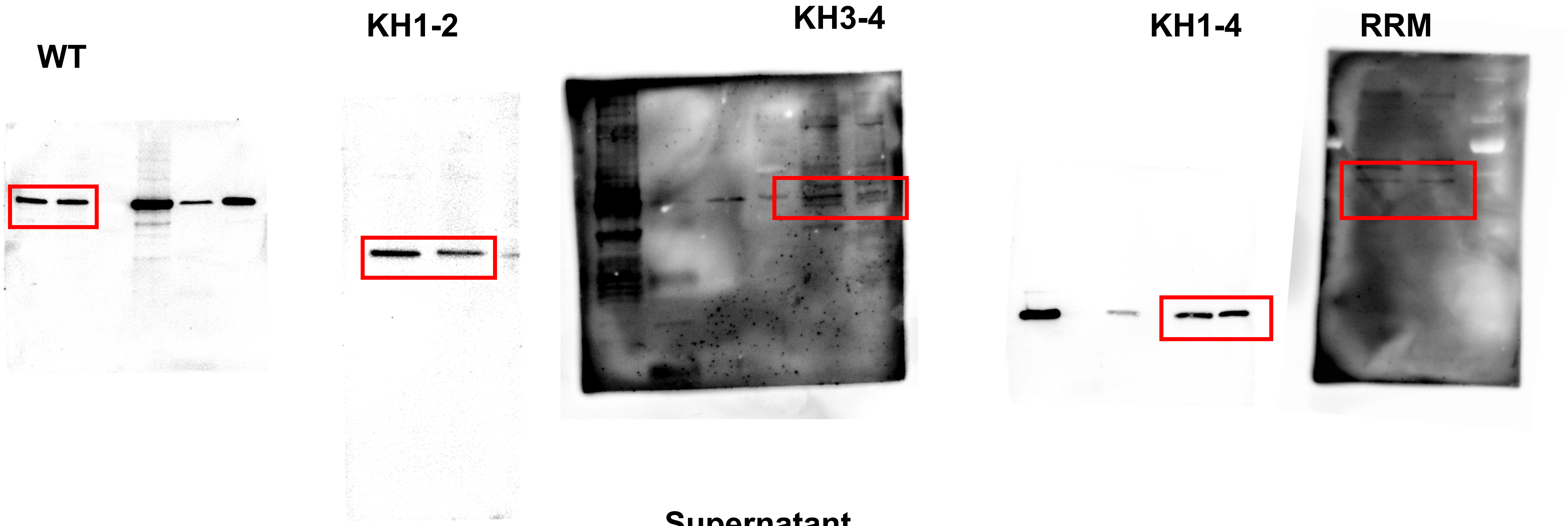


Supernatant

Figure 4A

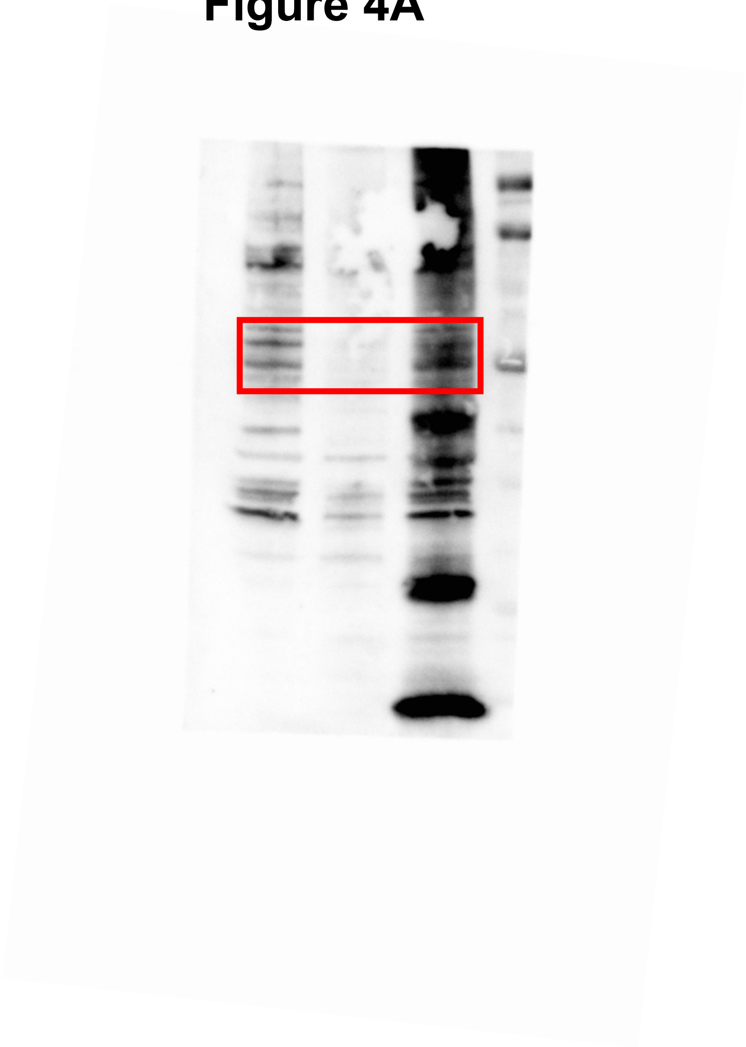

Figure 4B

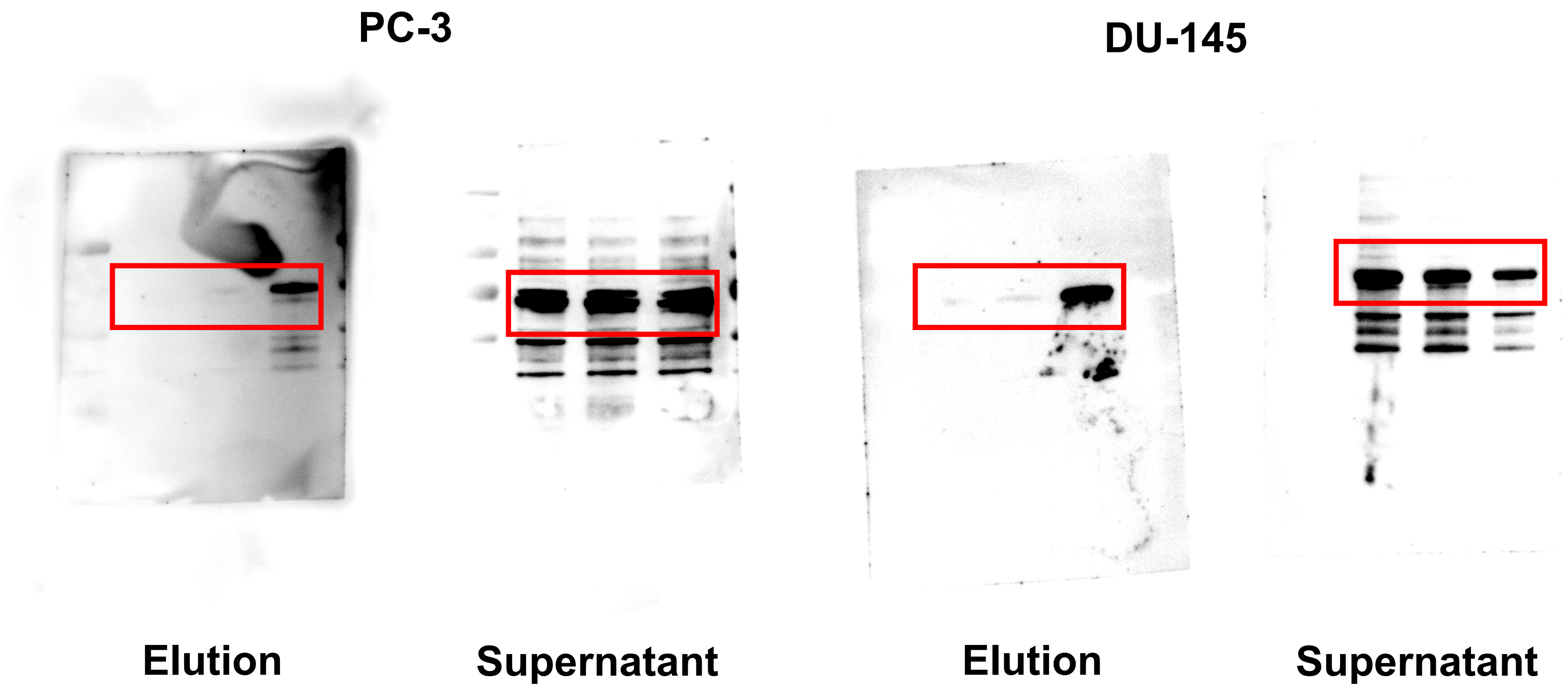

Figure 4C

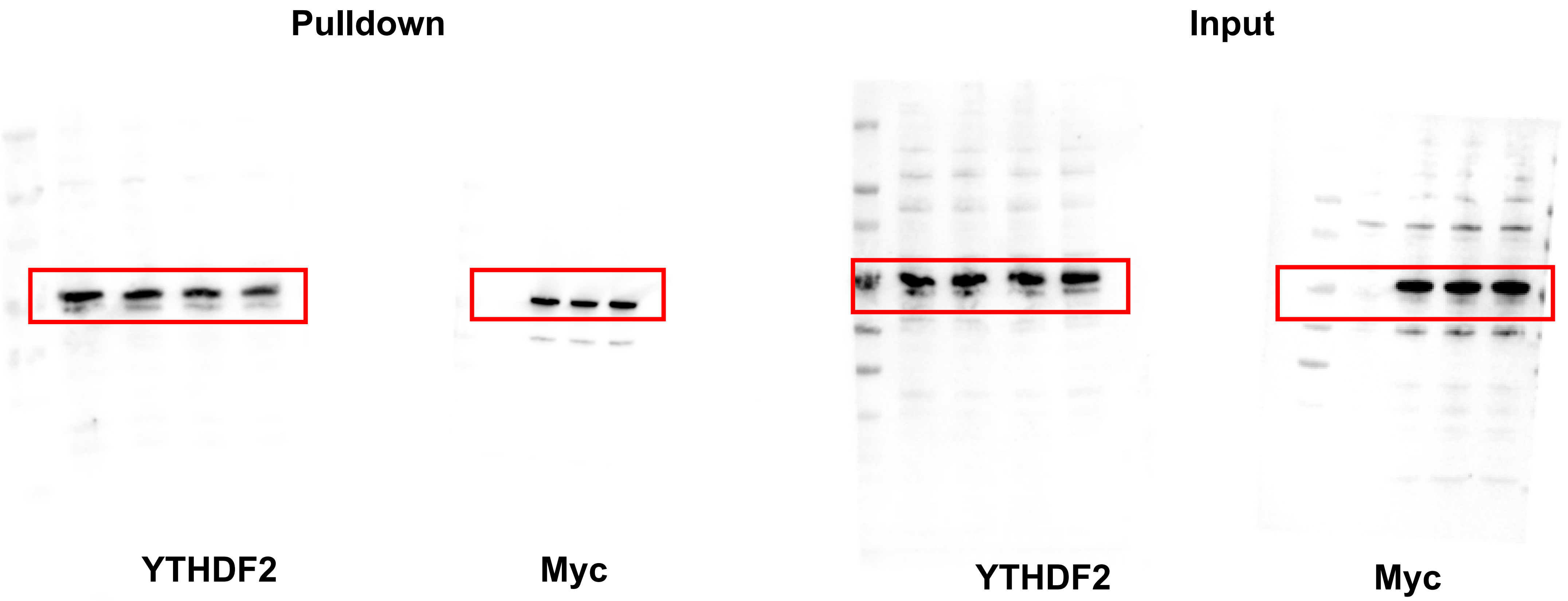

## Figure 4D

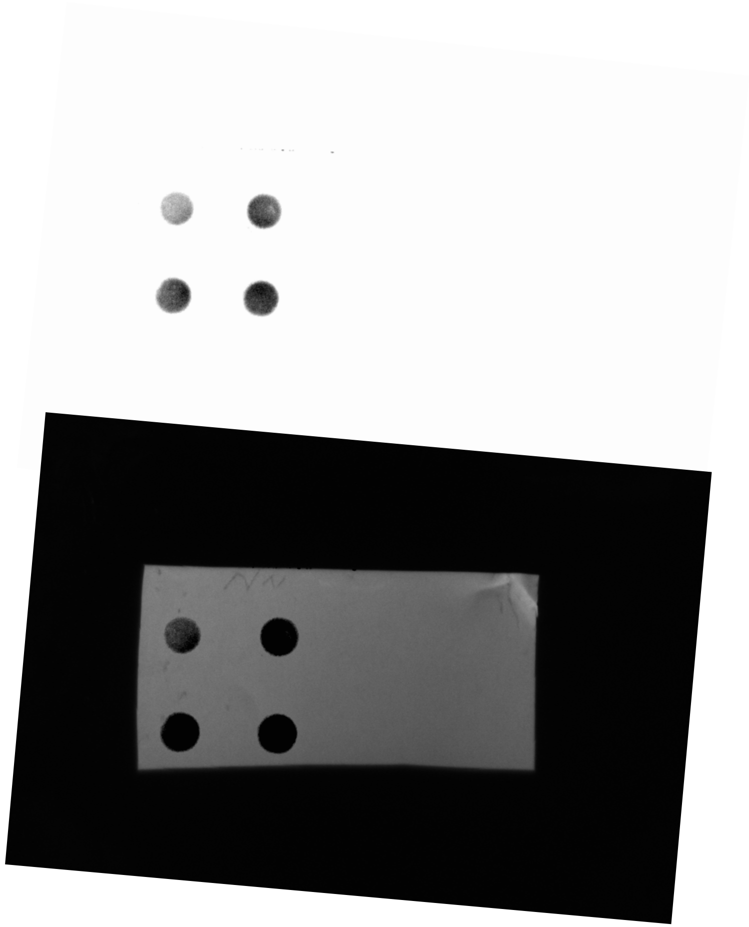

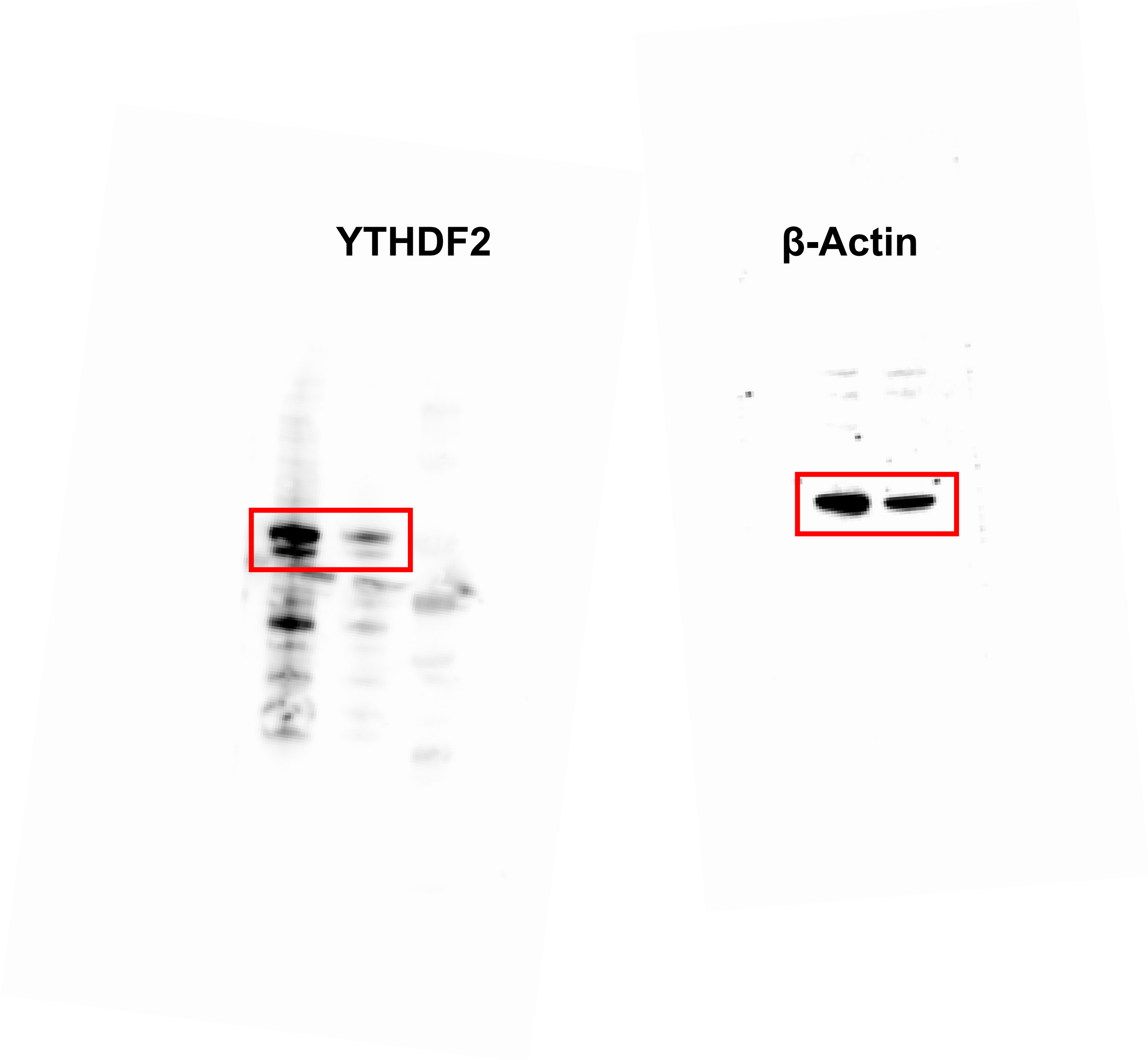

## Figure 6B

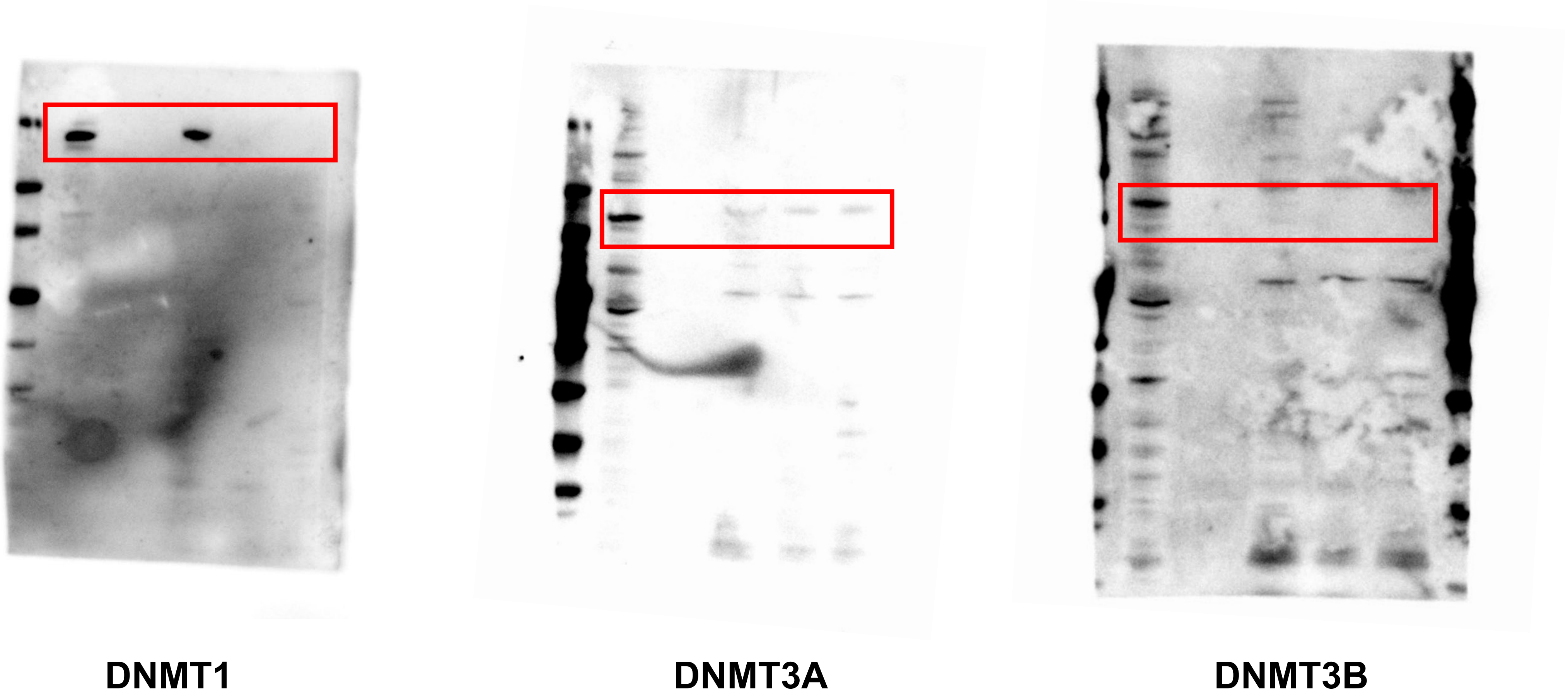

Figure 6C

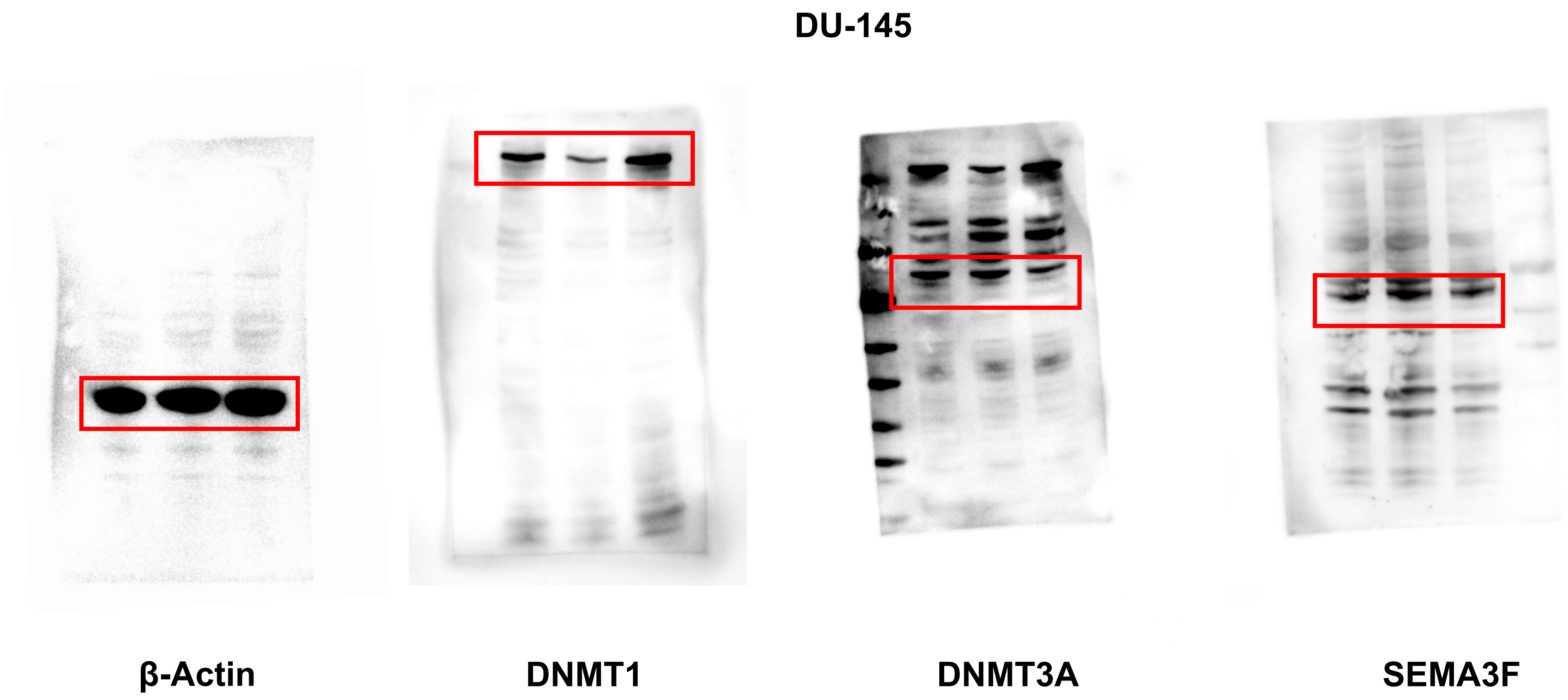

**Figure 6C**

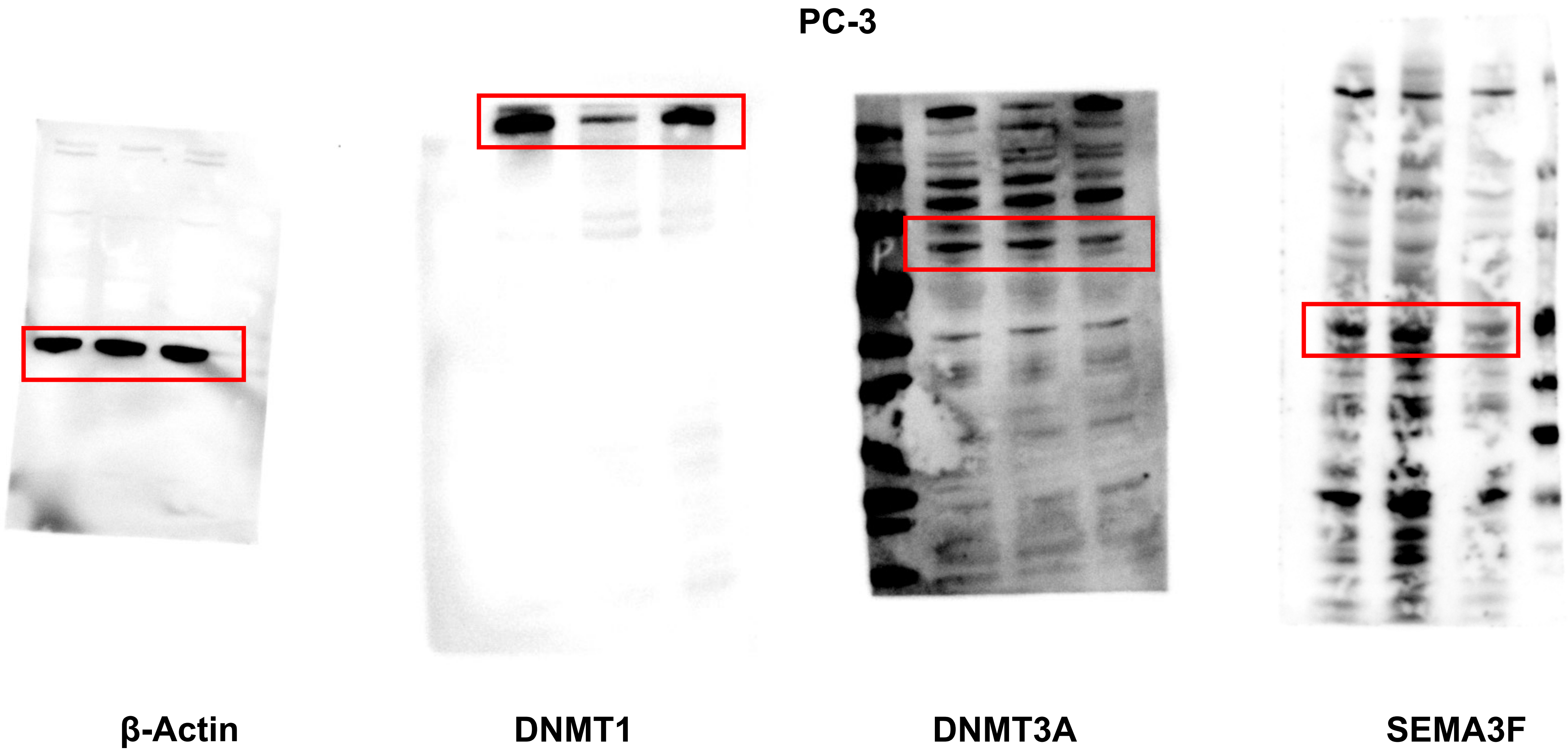

## Figure 7A

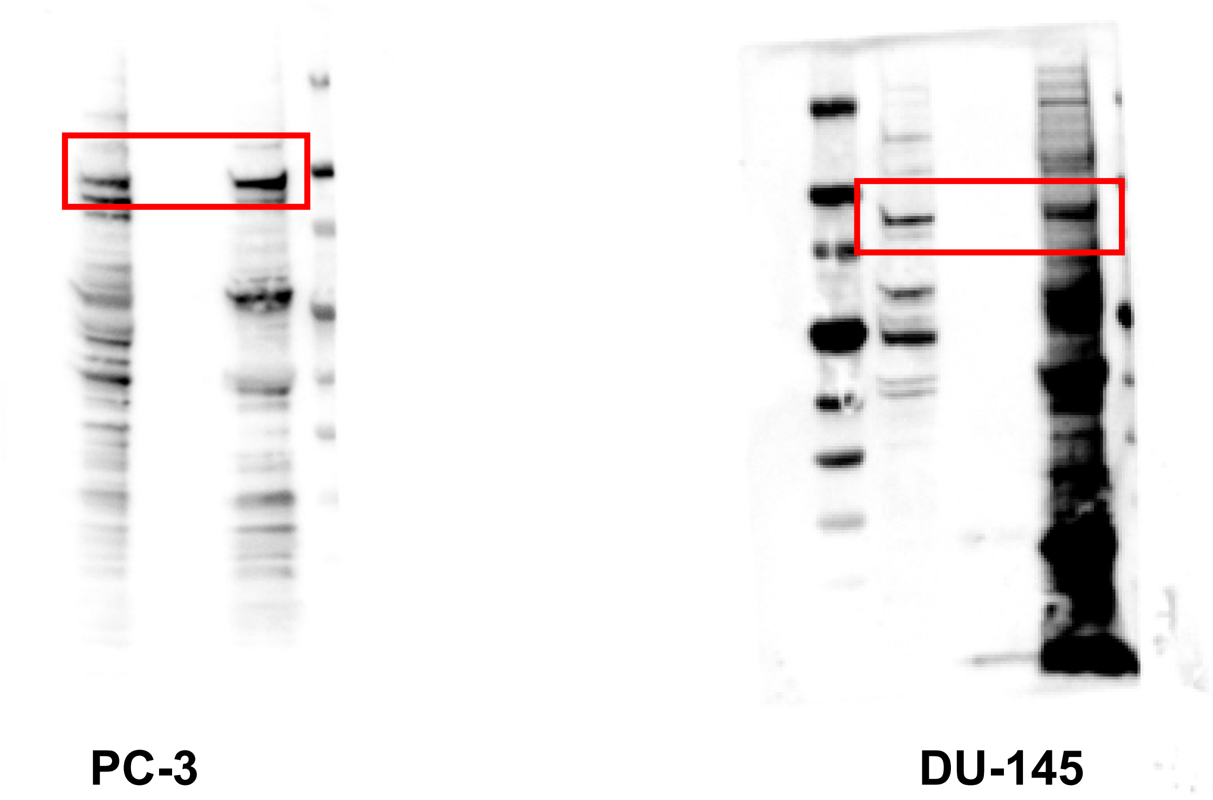

Figure 7B

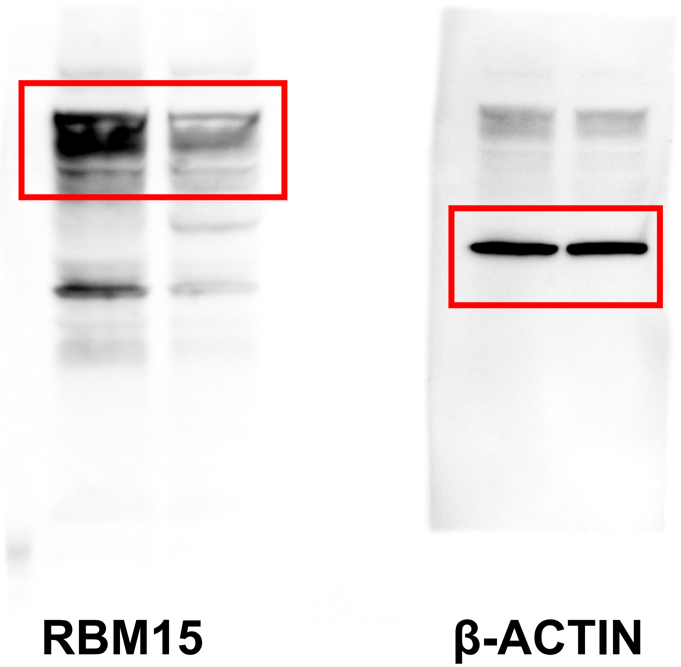

**Figure 7C**

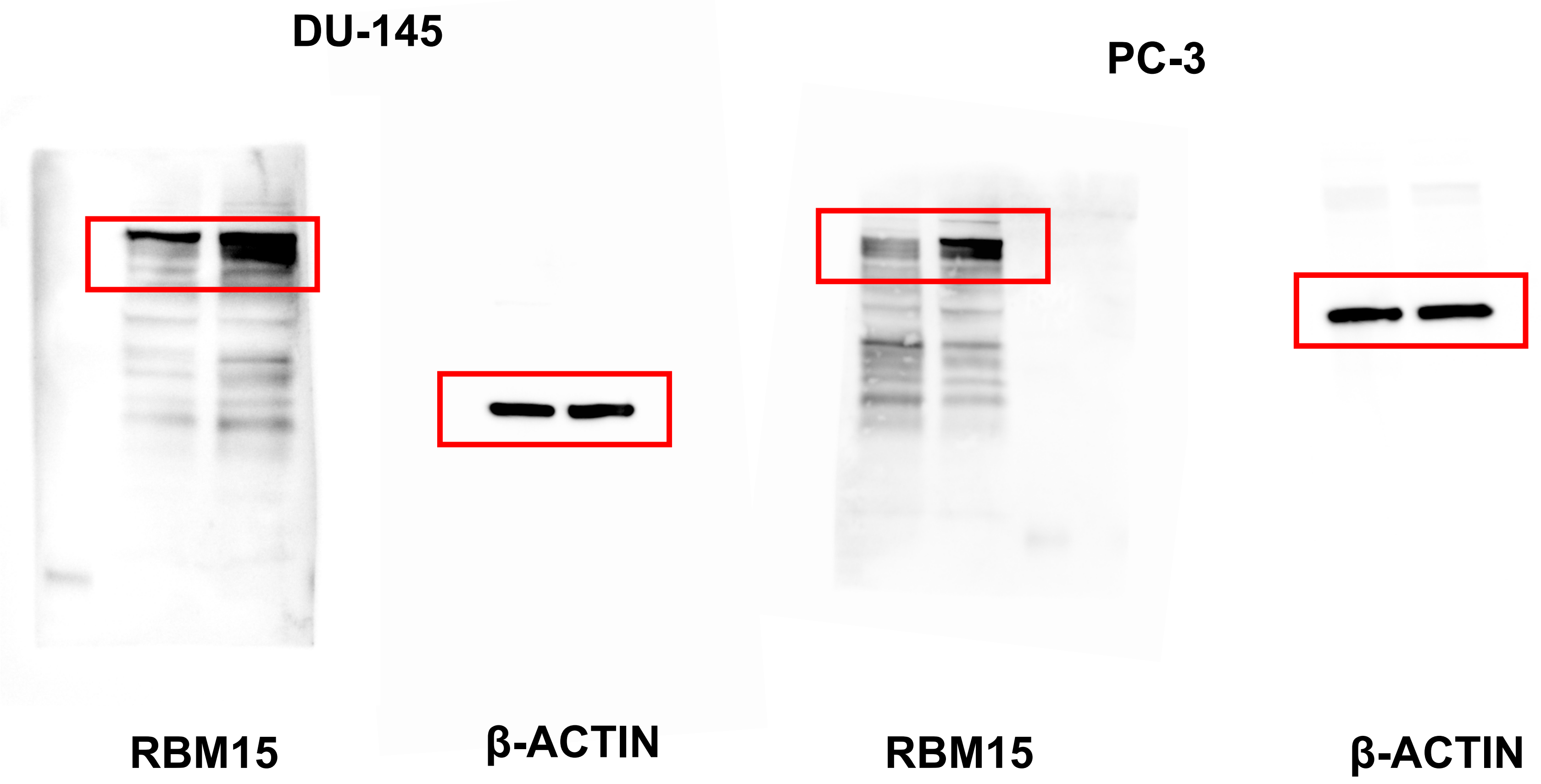

Figure 7 E

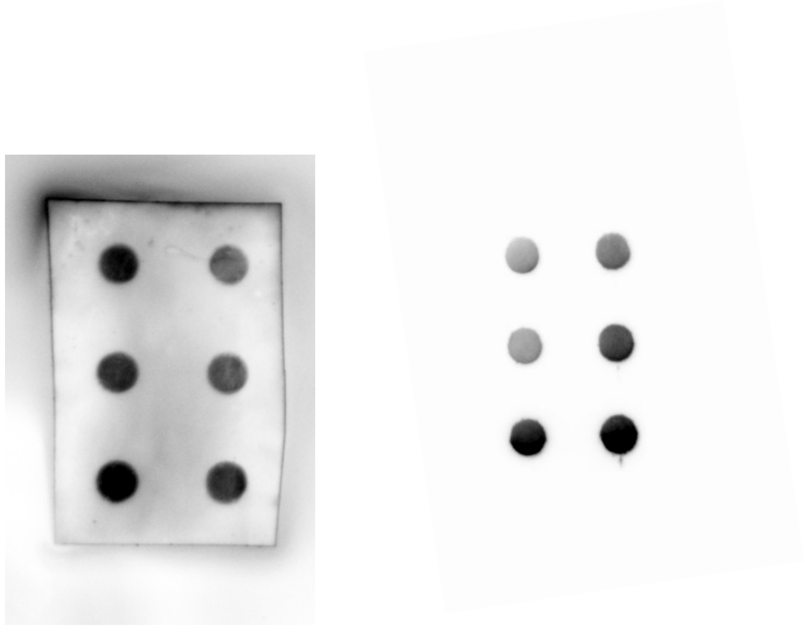

Cas9-RBM15

pc-RBM15

Figure 7 F

PC-3

DU-145

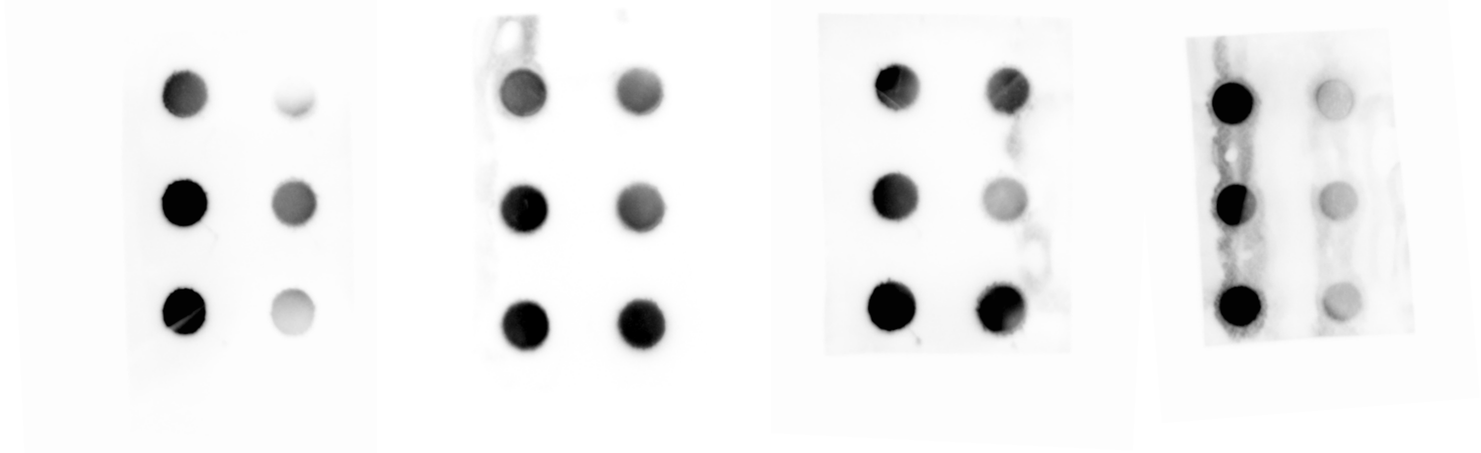

caRNA

Total RNA

caRNA

Total RNA

# Figure 7

## Figure 7H

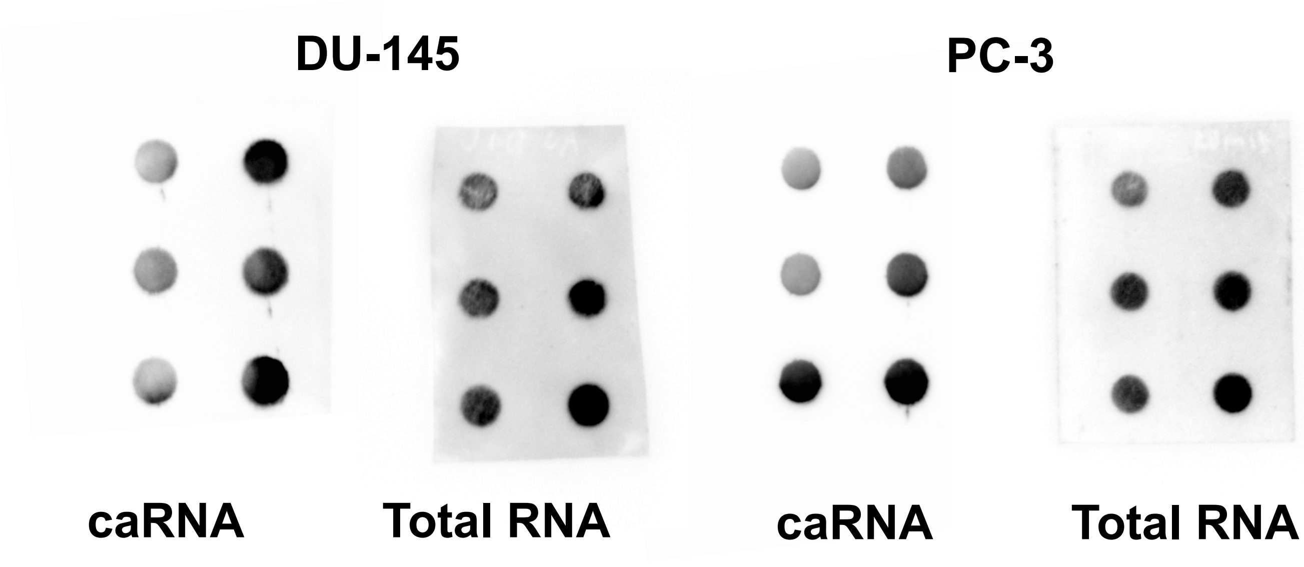

**Figure 8**

**Figure 8C**

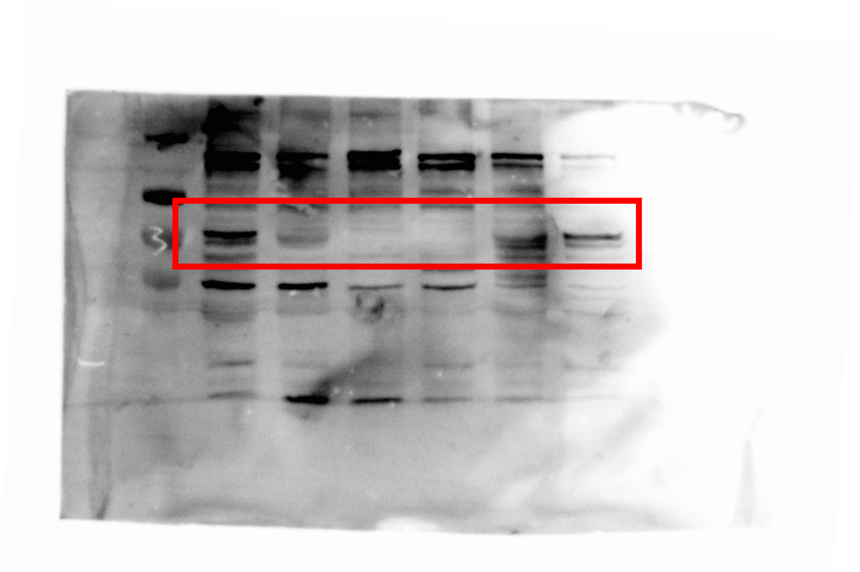

IGF2BP1

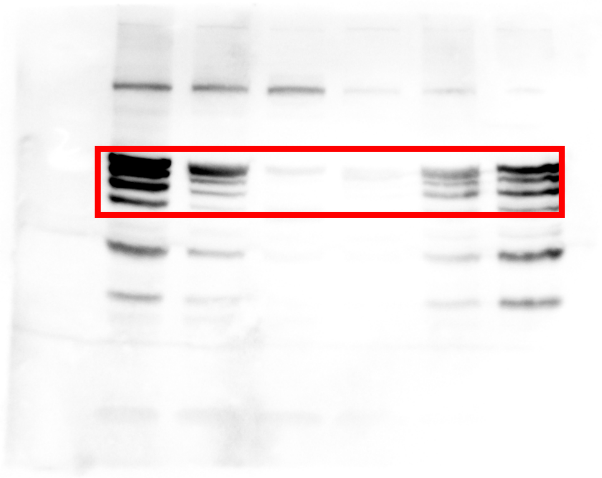

IGF2BP2

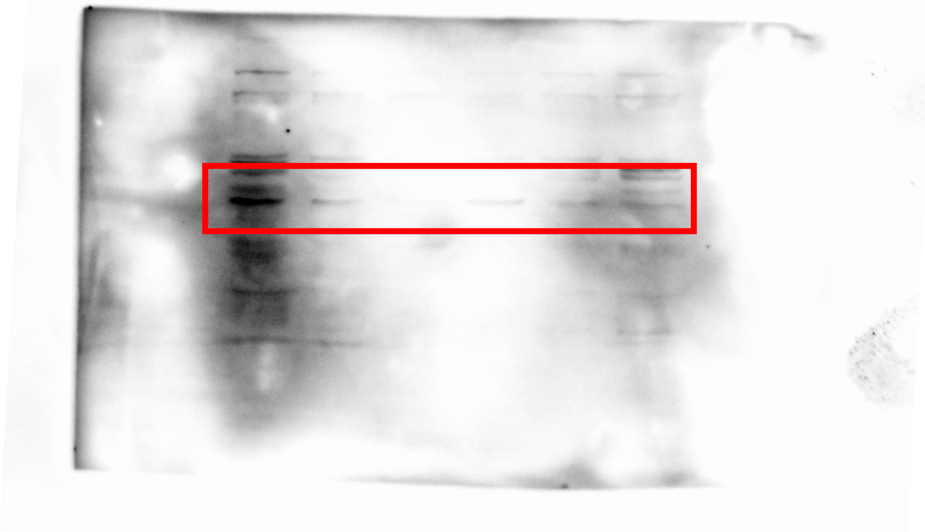

IGF2BP3

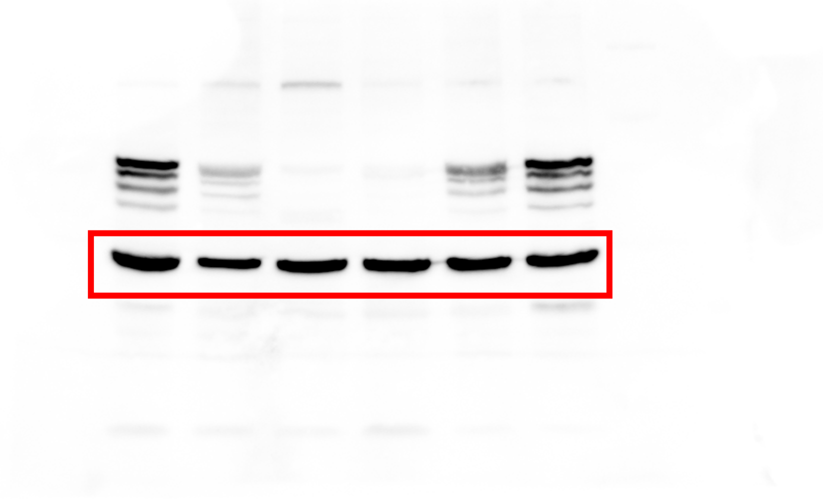

β-ACTIN

Figure 8

Figure 8 C

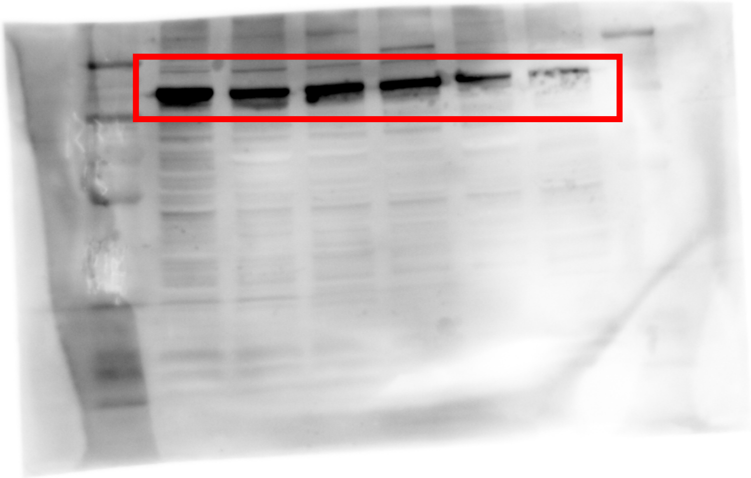

RBM15

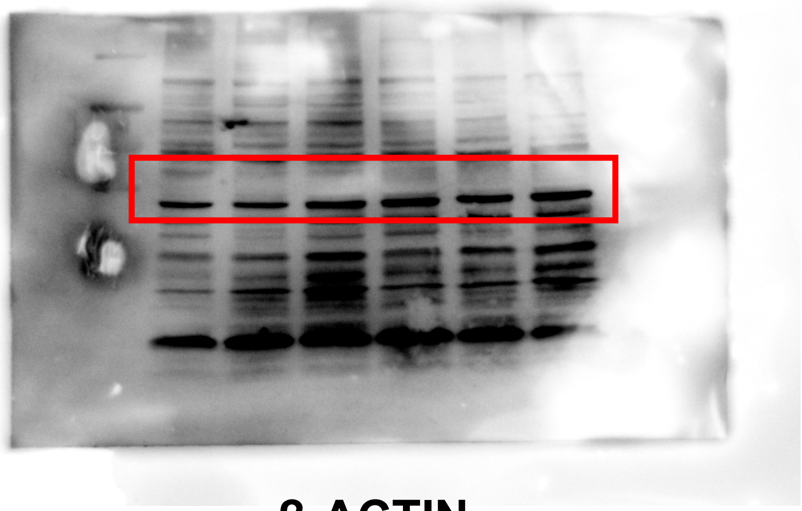

β-ACTIN

Figure 10

Figure 10 D

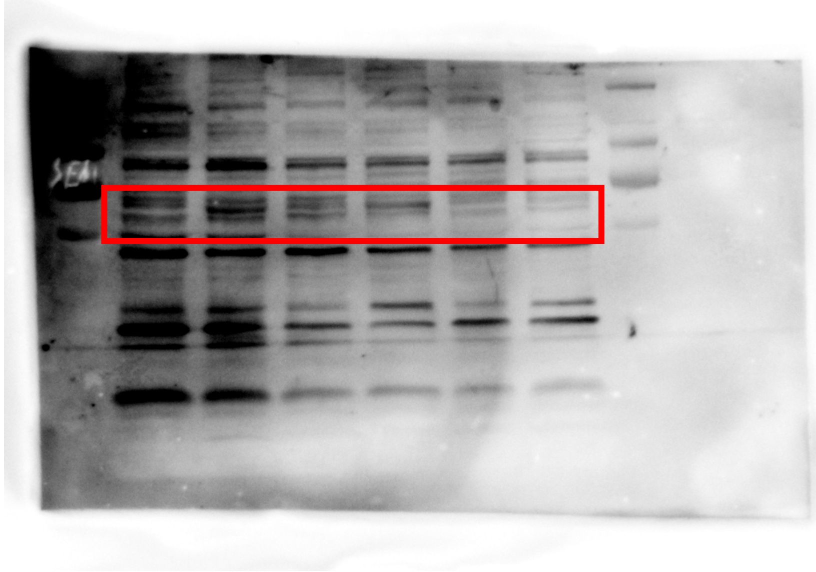

SEMA3F

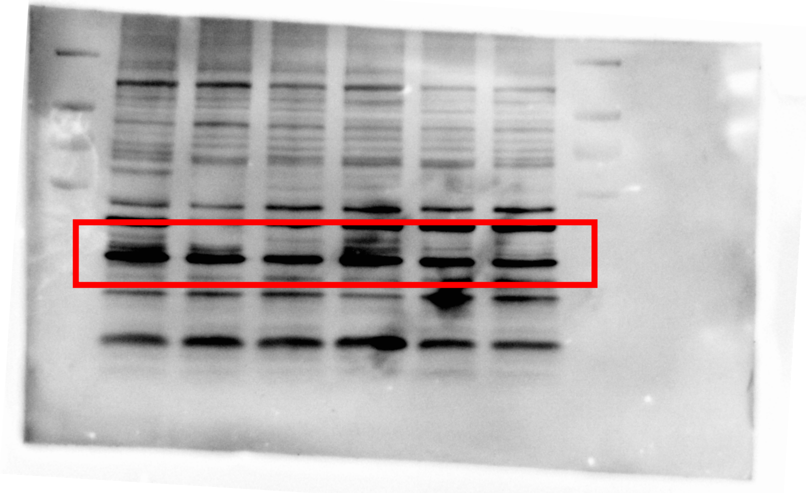

β-ACTIN

**Figure 10 I**

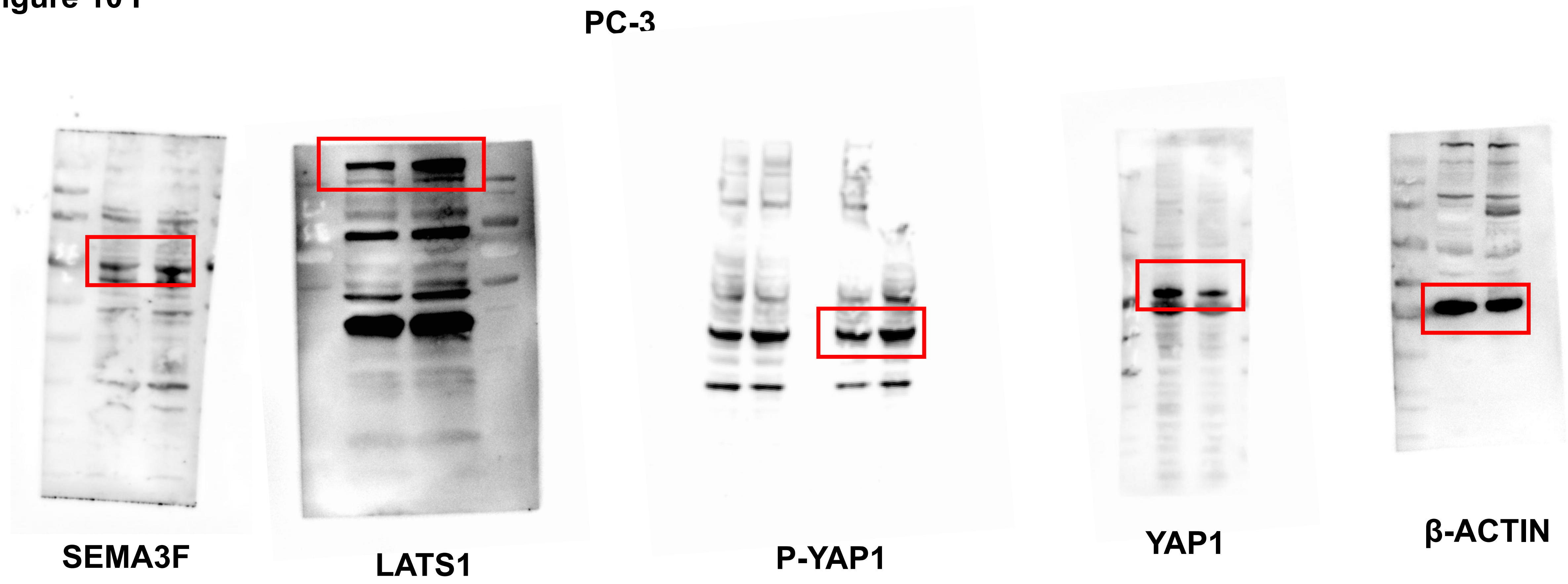

Figure 10 I

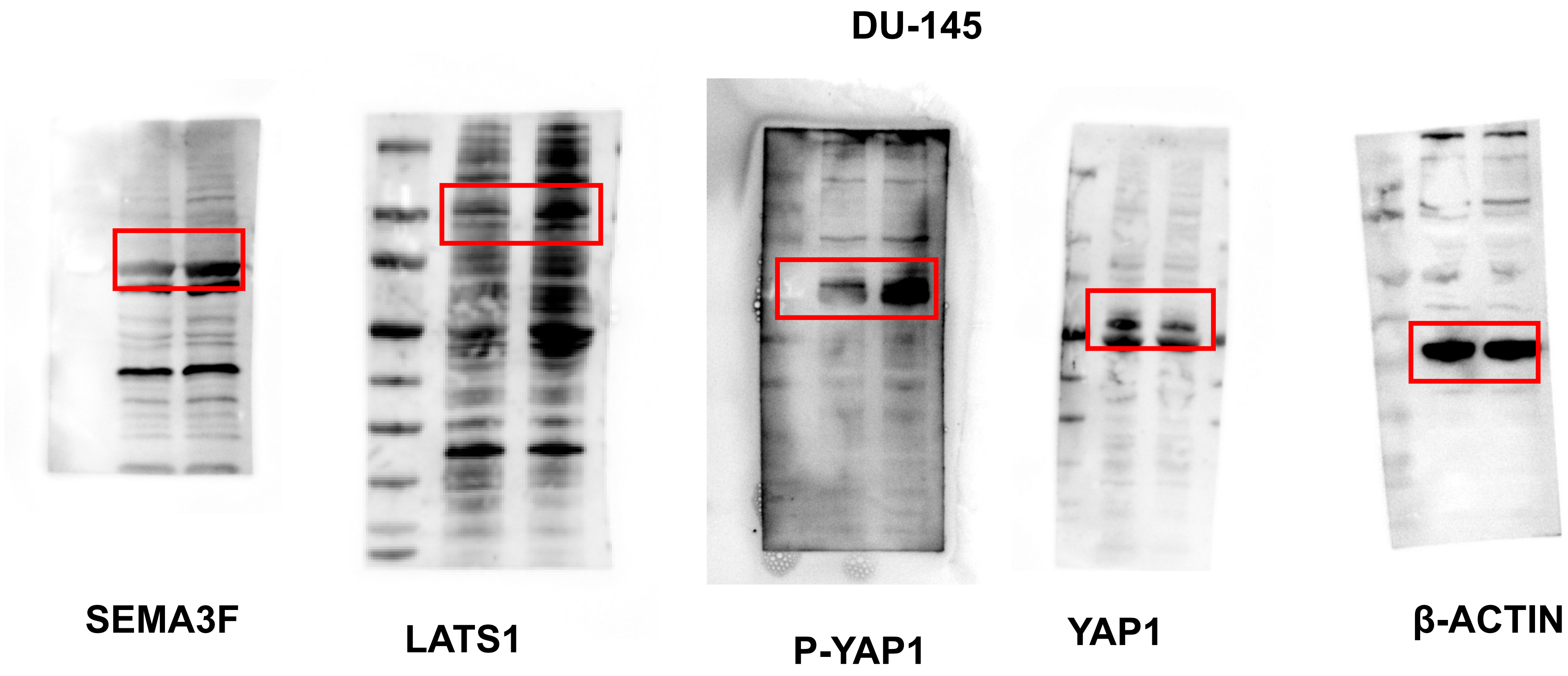

**Figure 10 J**

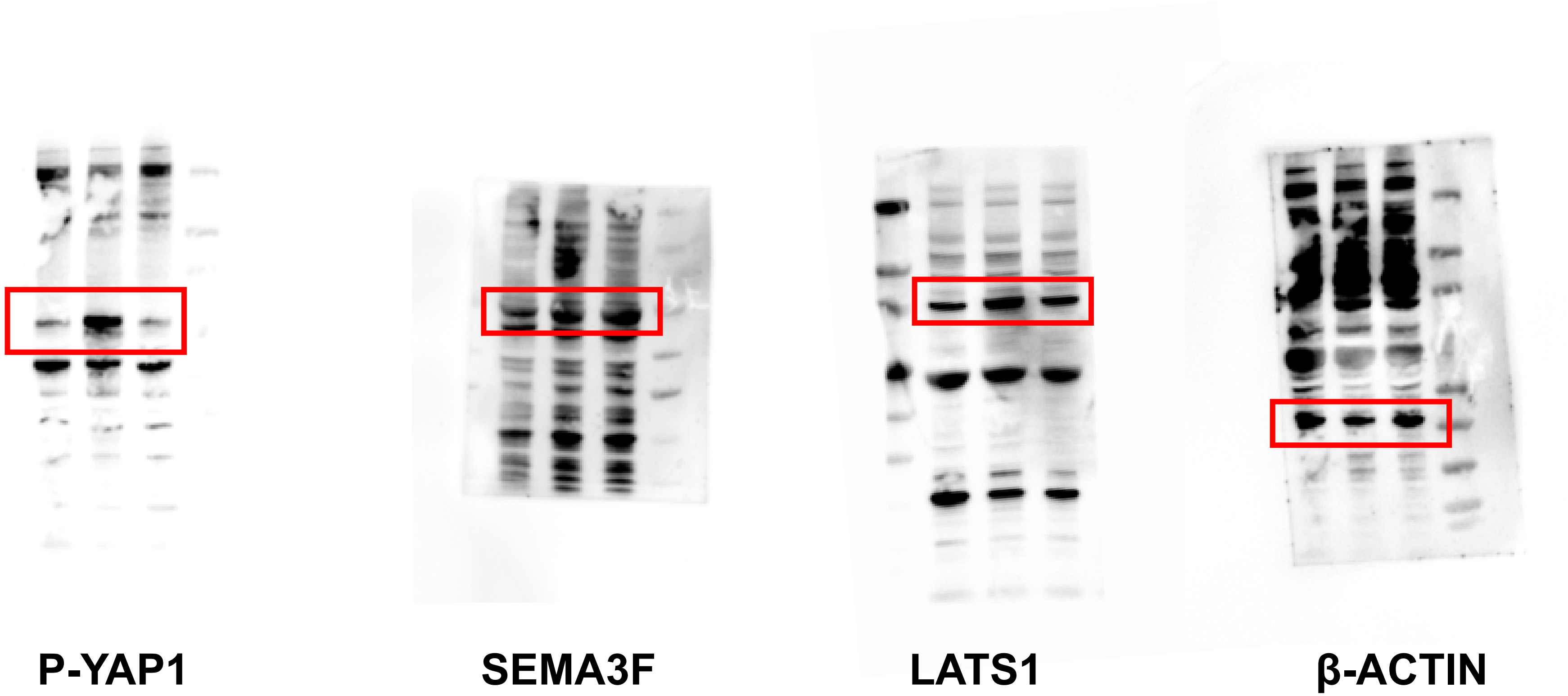

P-YAP1 SEMA3F LATS1 β-ACTIN

# Supplementary Figure 1

## Supplementary Figure 1 A

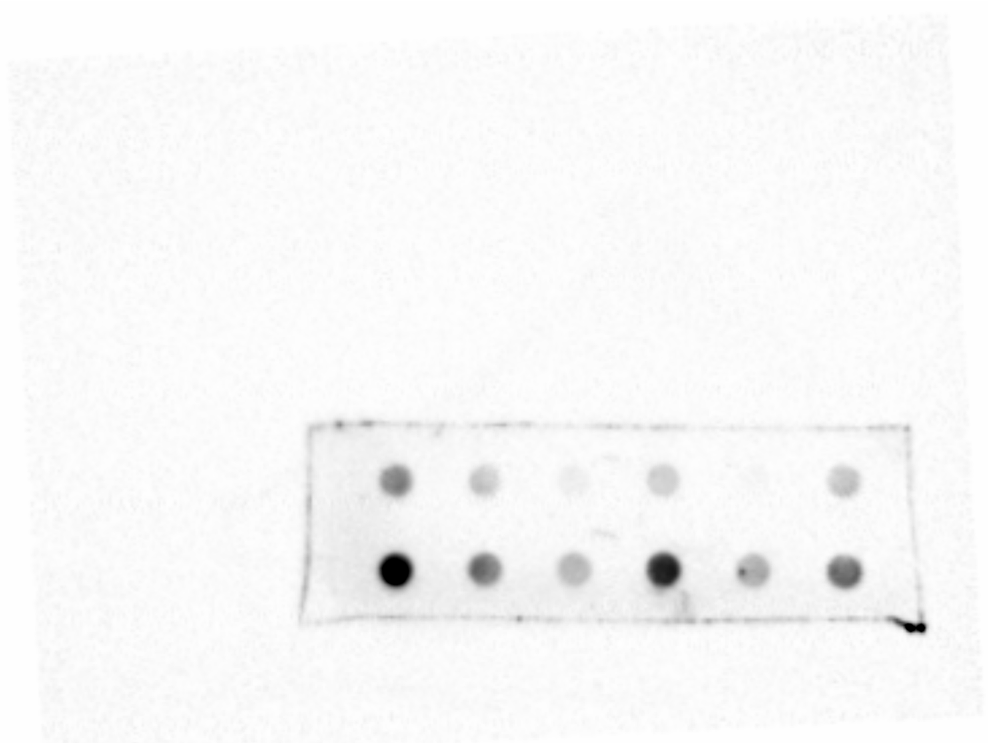

**Supplementary Figure 1 C**

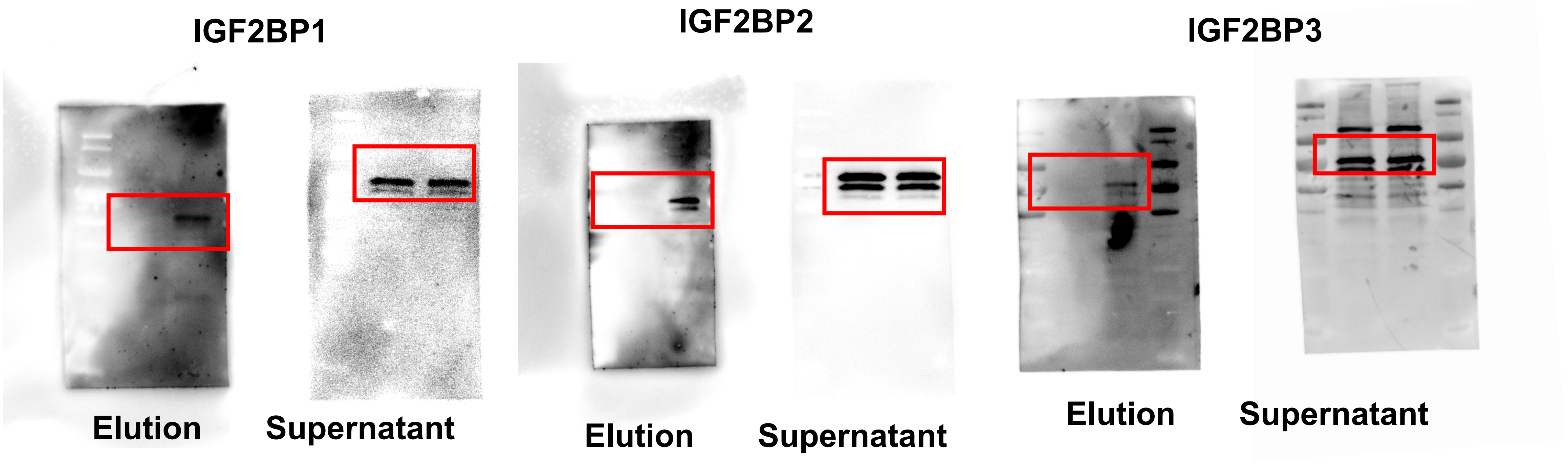

Supplementary
Figure 1 D

Elution:IGF2BP1

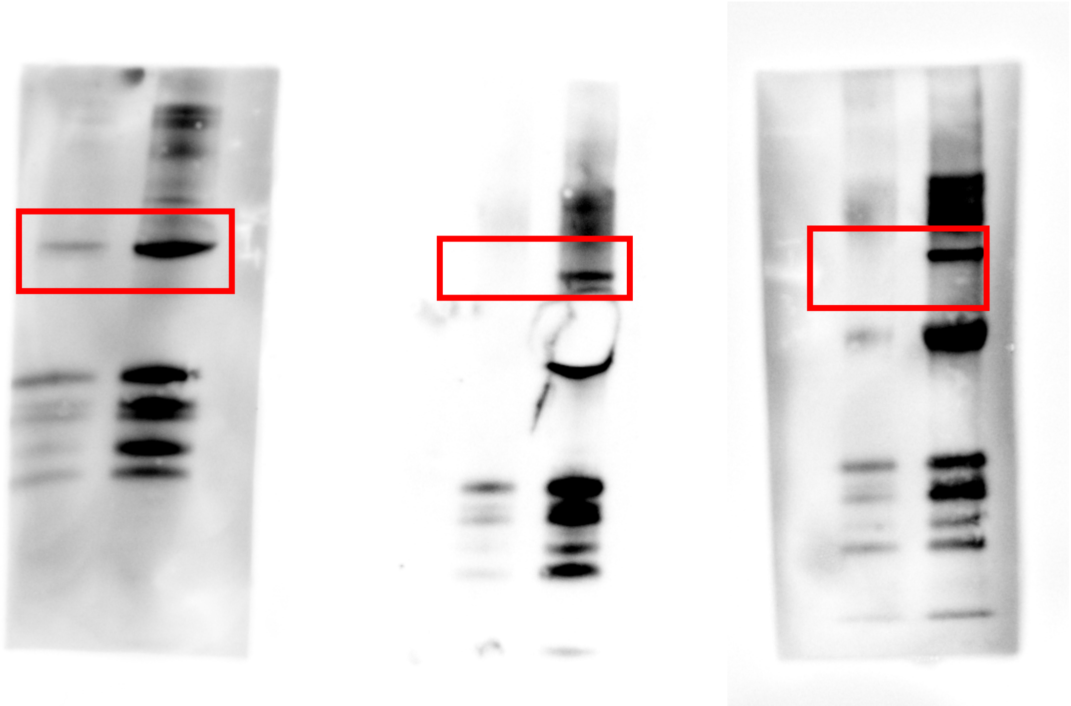

Myc-IGF2BP1
Myc IP

Myc-IGF2BP2
Myc IP

Myc-IGF2BP3
Myc IP

Supplementary
Figure 1 D

Elution:IGF2BP2

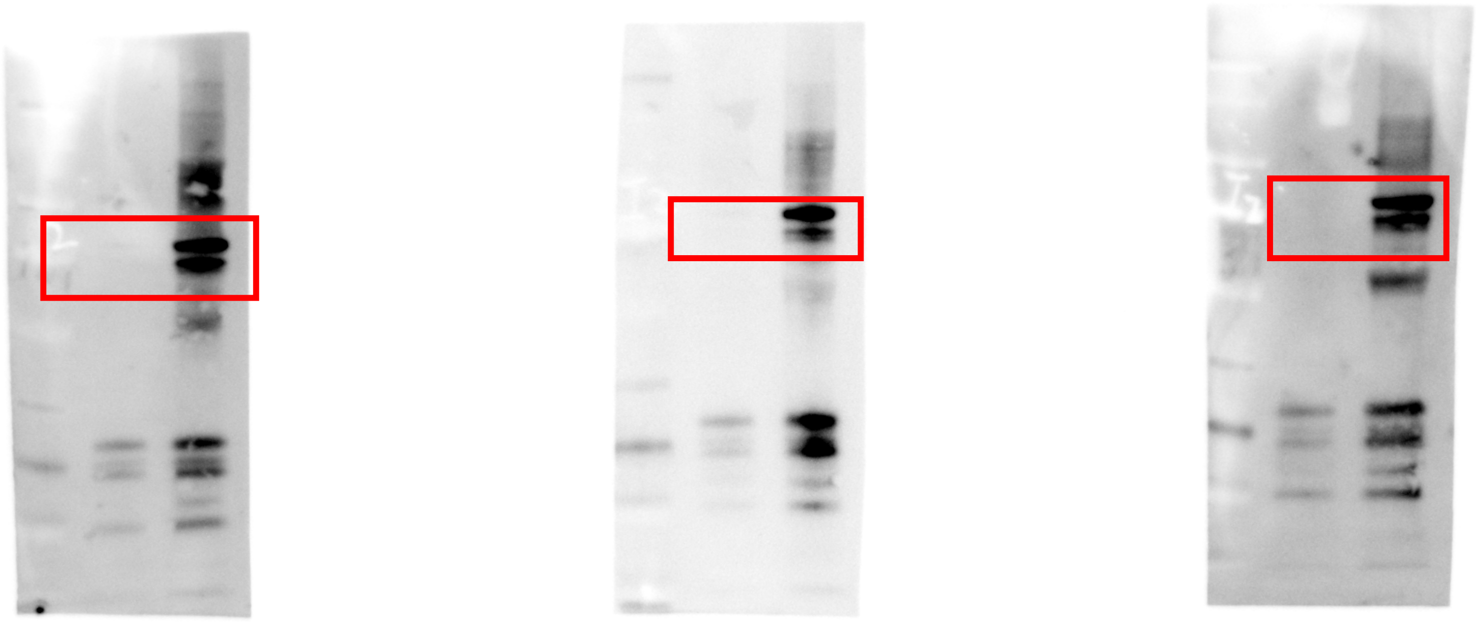

Myc-IGF2BP1
Myc IP

Myc-IGF2BP2
Myc IP

Myc-IGF2BP3
Myc IP

Supplementary
Figure 1 D

Elution:IGF2BP3

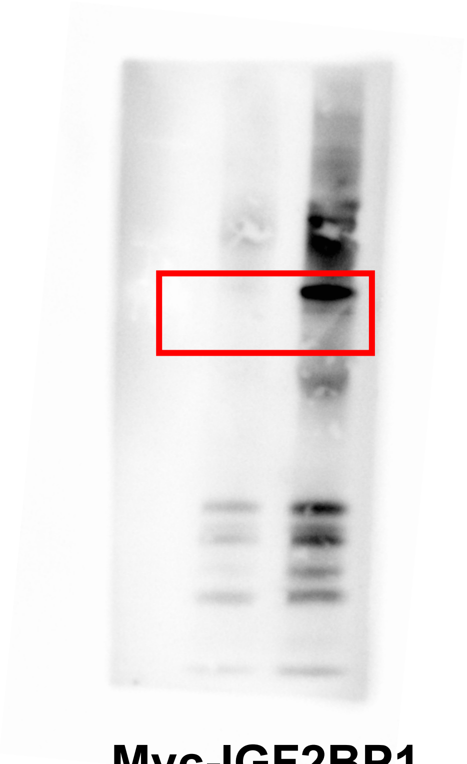

Myc-IGF2BP1
Myc IP

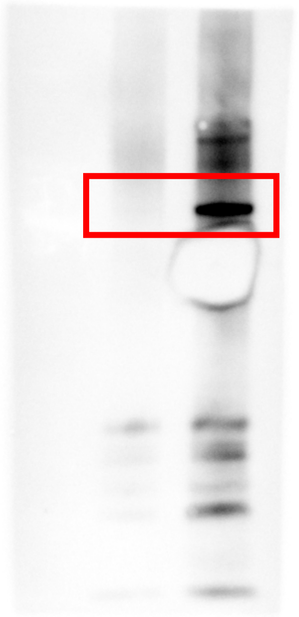

Myc-IGF2BP2
Myc IP

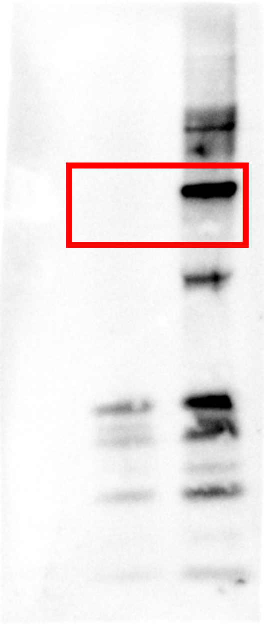

Myc-IGF2BP3
Myc IP

## Supplementary Figure 1

## Supplementary Figure 1 D

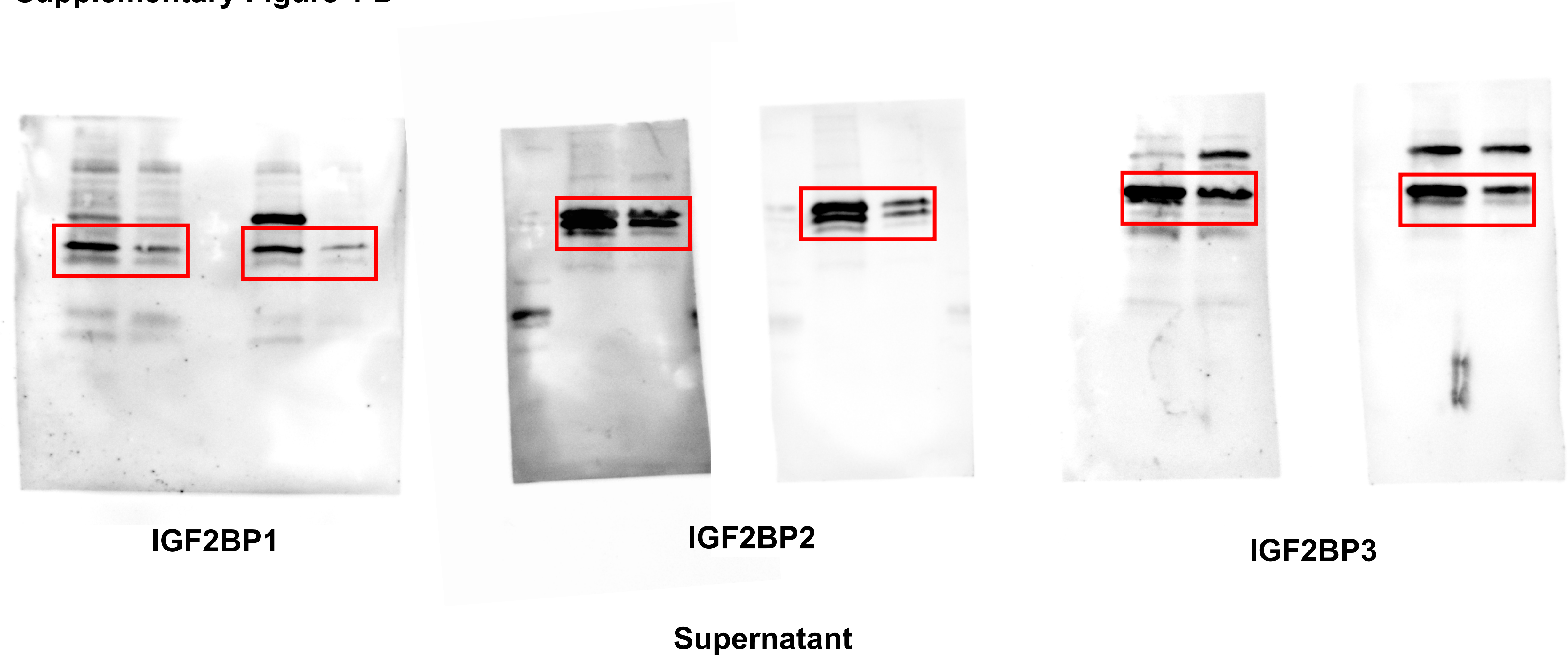

Supplementary
Figure 2 B

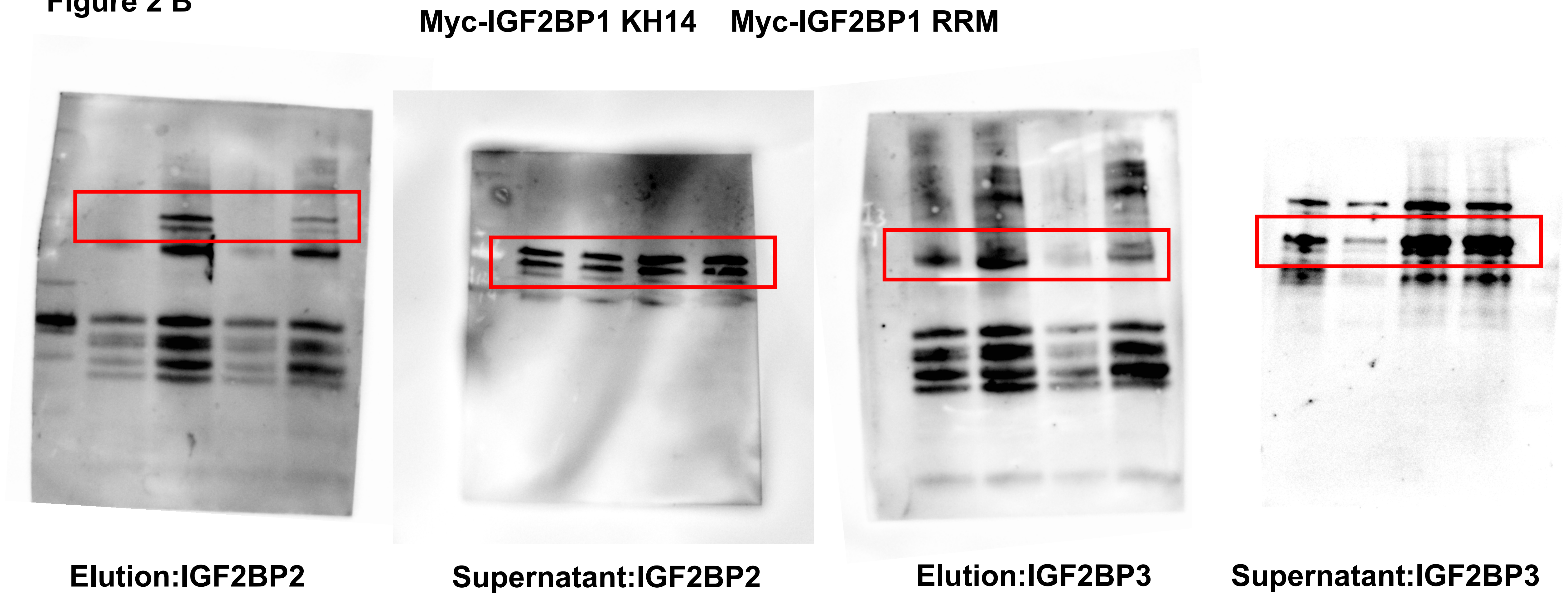

Supplementary Figure 2 C

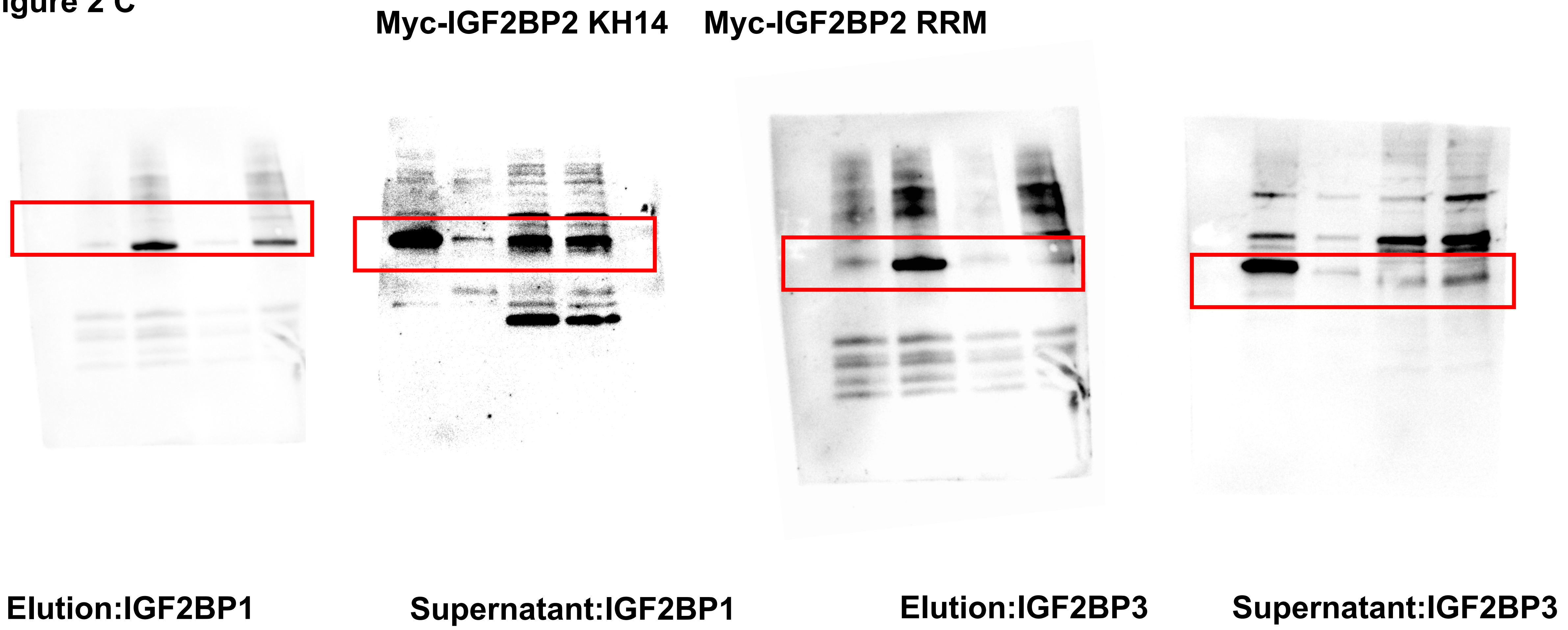

Supplementary Figure 2 D

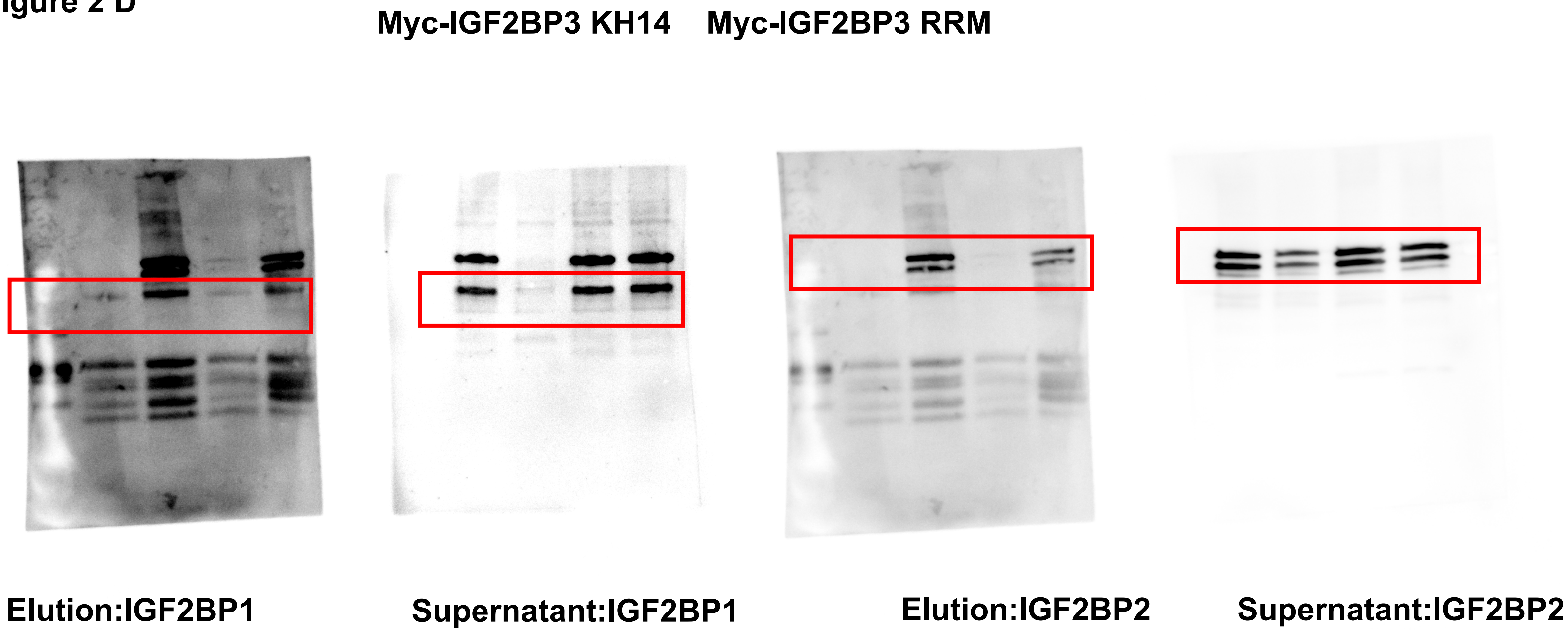

Supplementary
Figure 2 B

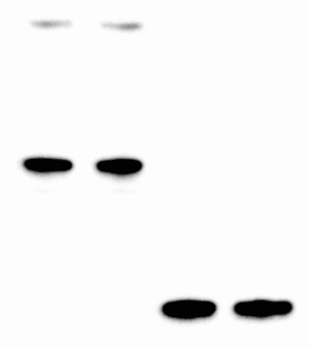

Input:Myc-IGF2BP1

Supplementary
Figure 2 C

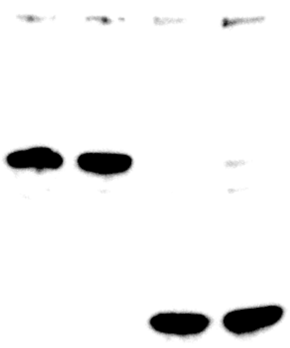

Input:Myc-IGF2BP2

Supplementary
Figure 2 D

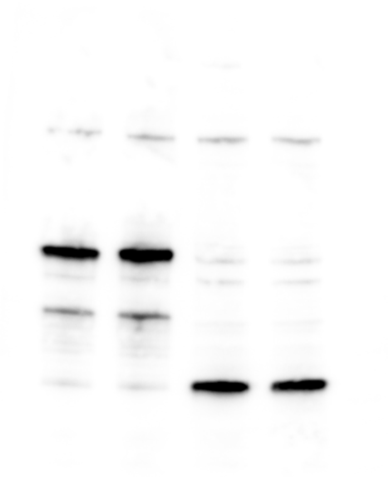

Input:Myc-IGF2BP3

**Supplementary Figure 3E**

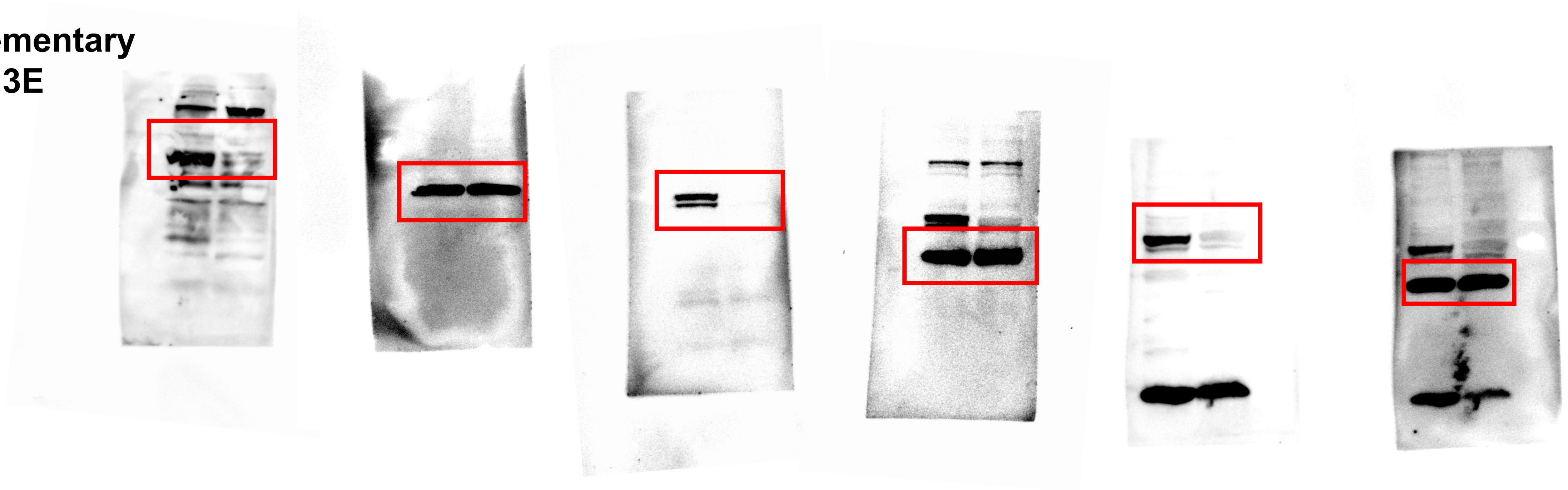

IGF2BP1 β-ACTIN IGF2BP2 β-ACTIN IGF2BP3 β-ACTIN

**Supplementary Figure 4A**

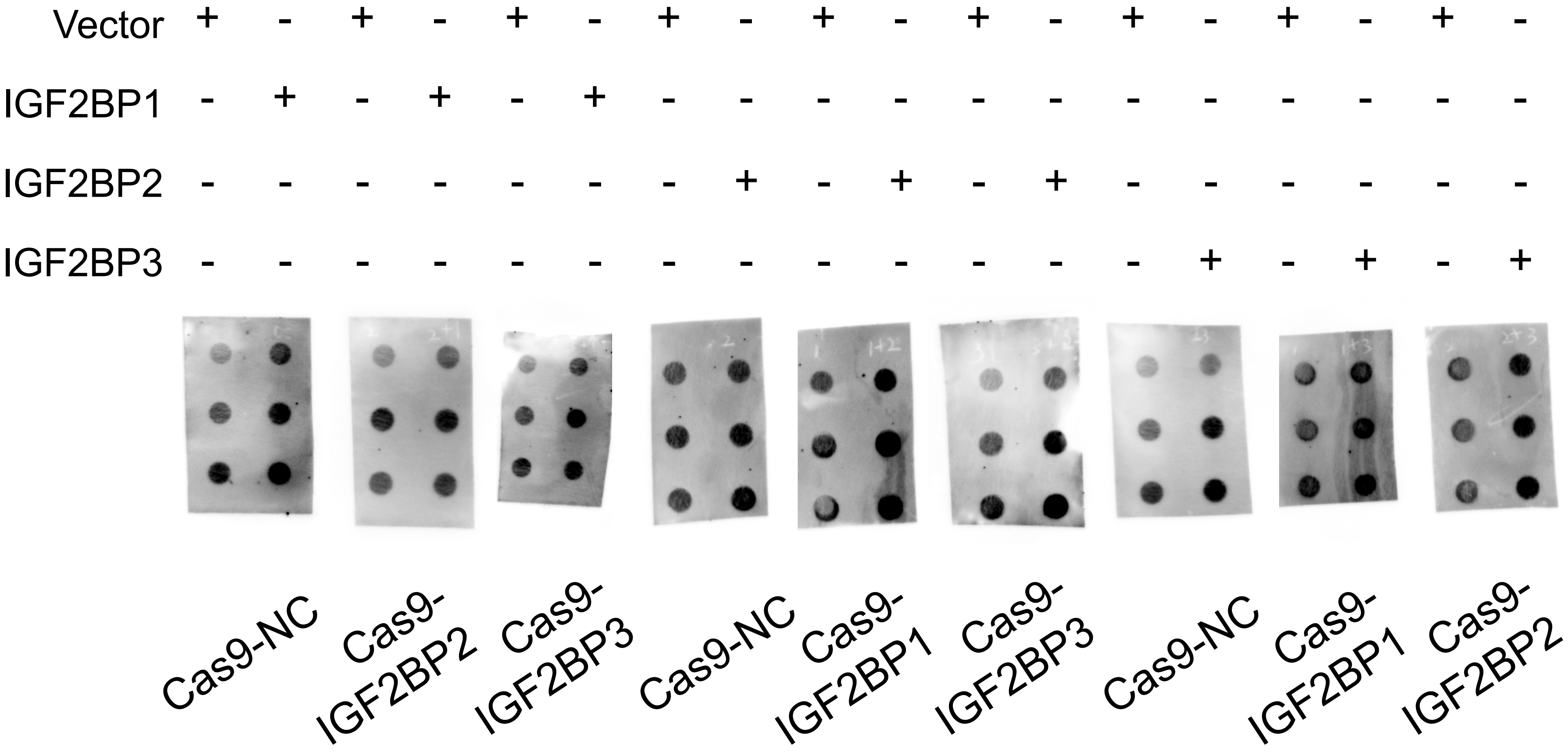

**Supplementary Figure 6 E**

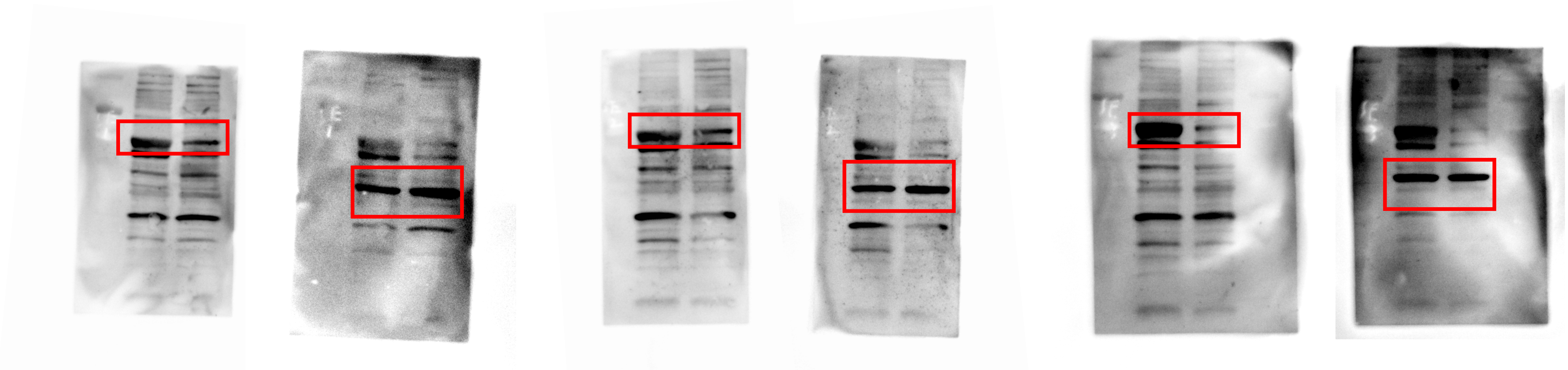

SEMA3F β-ACTIN SEMA3F β-ACTIN SEMA3F β-ACTIN

**Supplementary Figure 7 C**

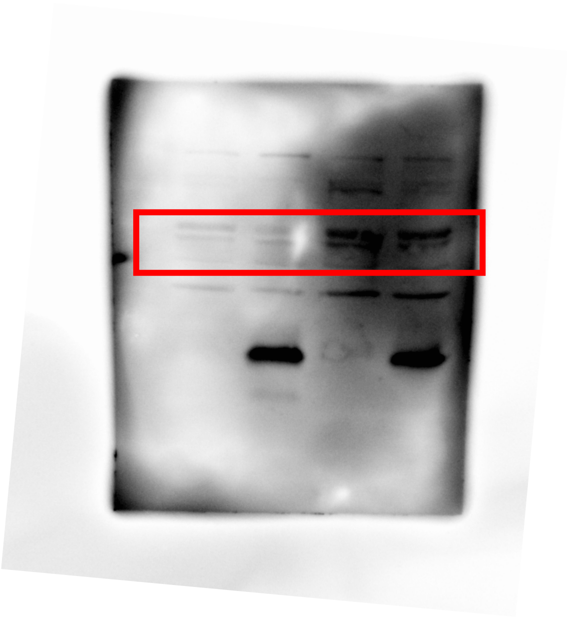

IGF2BP1

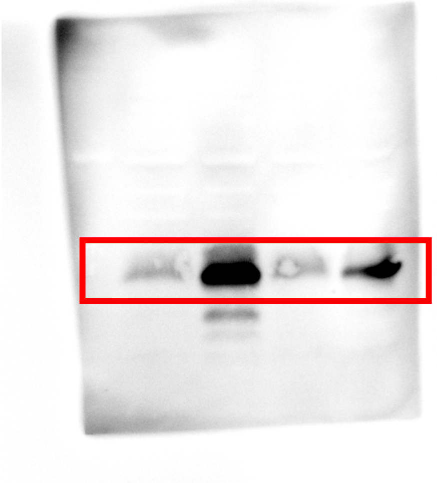

RNaseH1

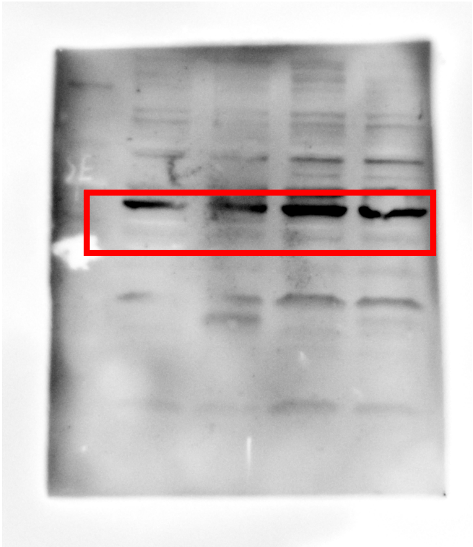

SEMA3F

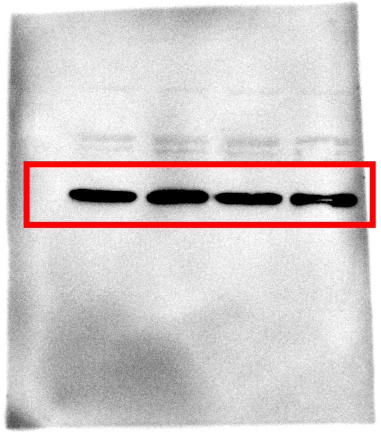

β-ACTIN

**Supplementary Figure 7 C**

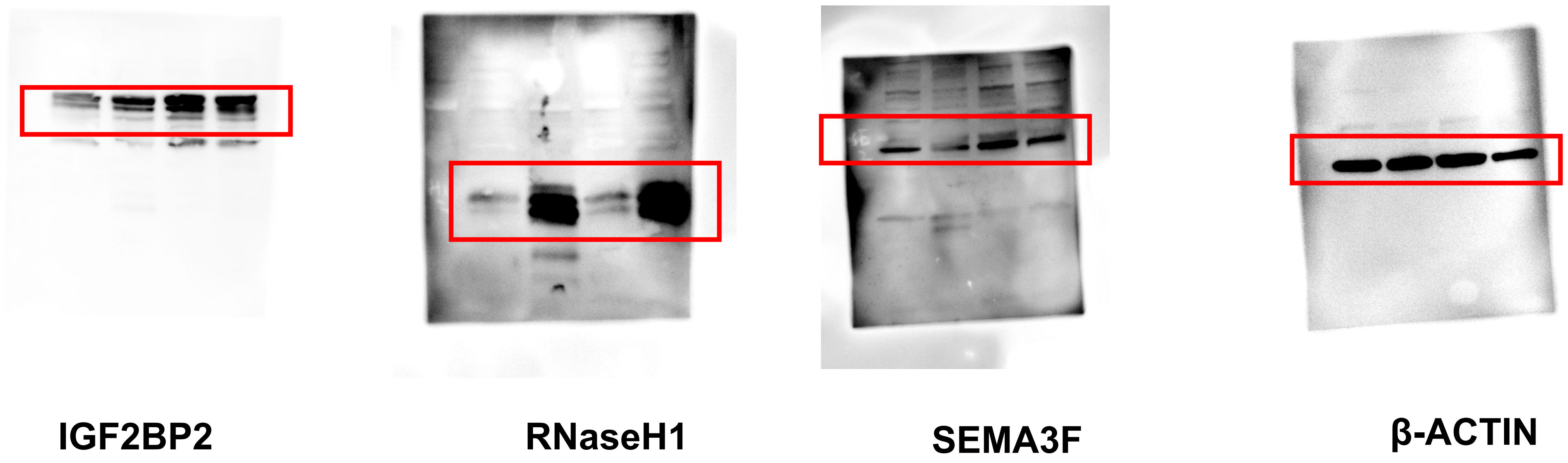

Supplementary Figure 7 C

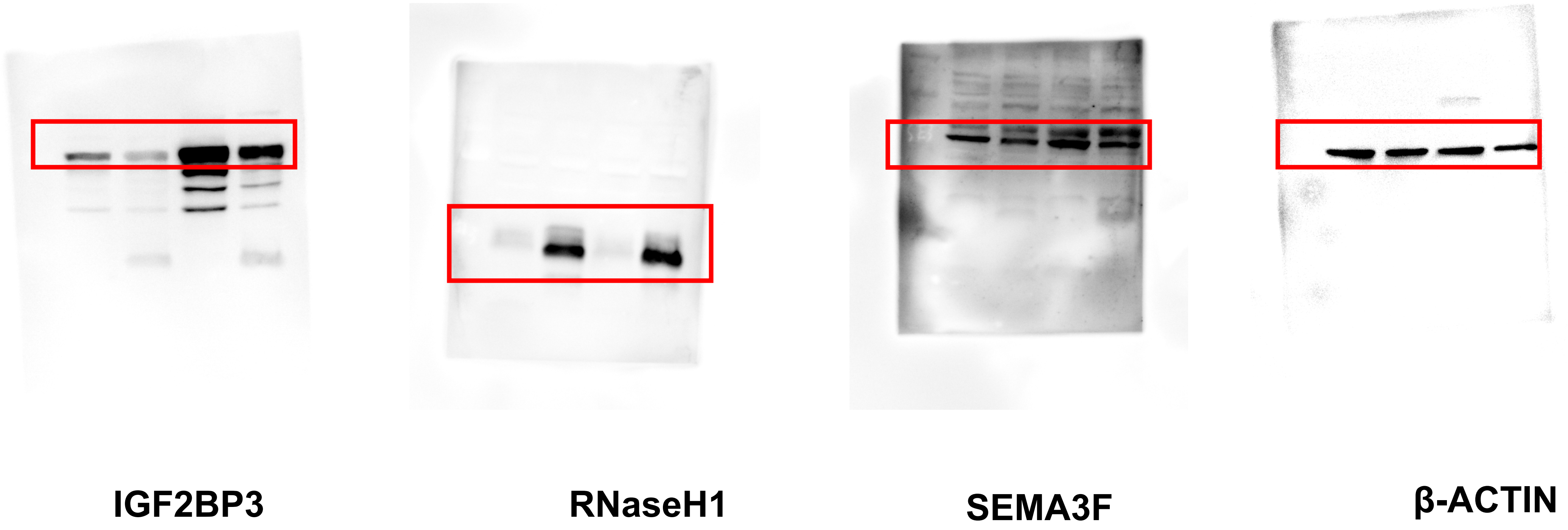

**Supplementary Figure 8 A**

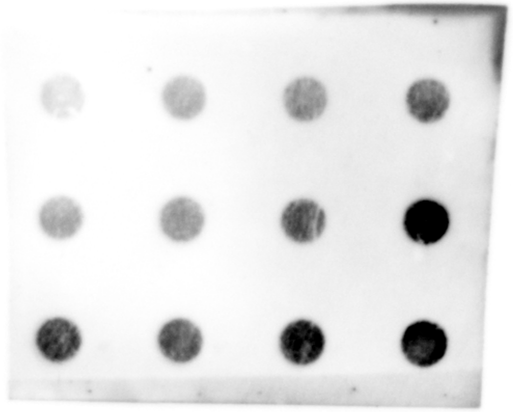

IGF2BP1

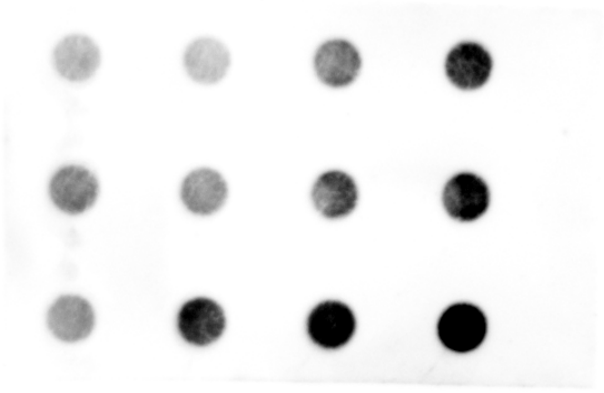

IGF2BP2

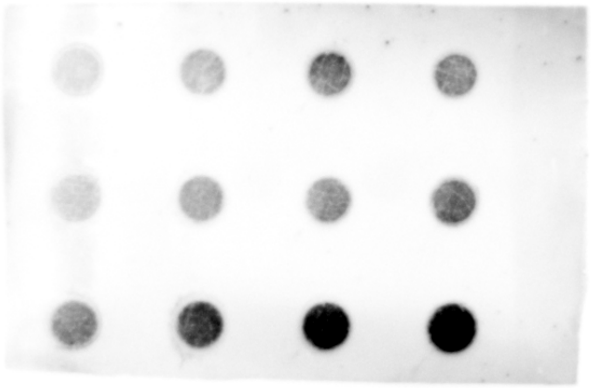

IGF2BP3

Supplementary Figure 8 C

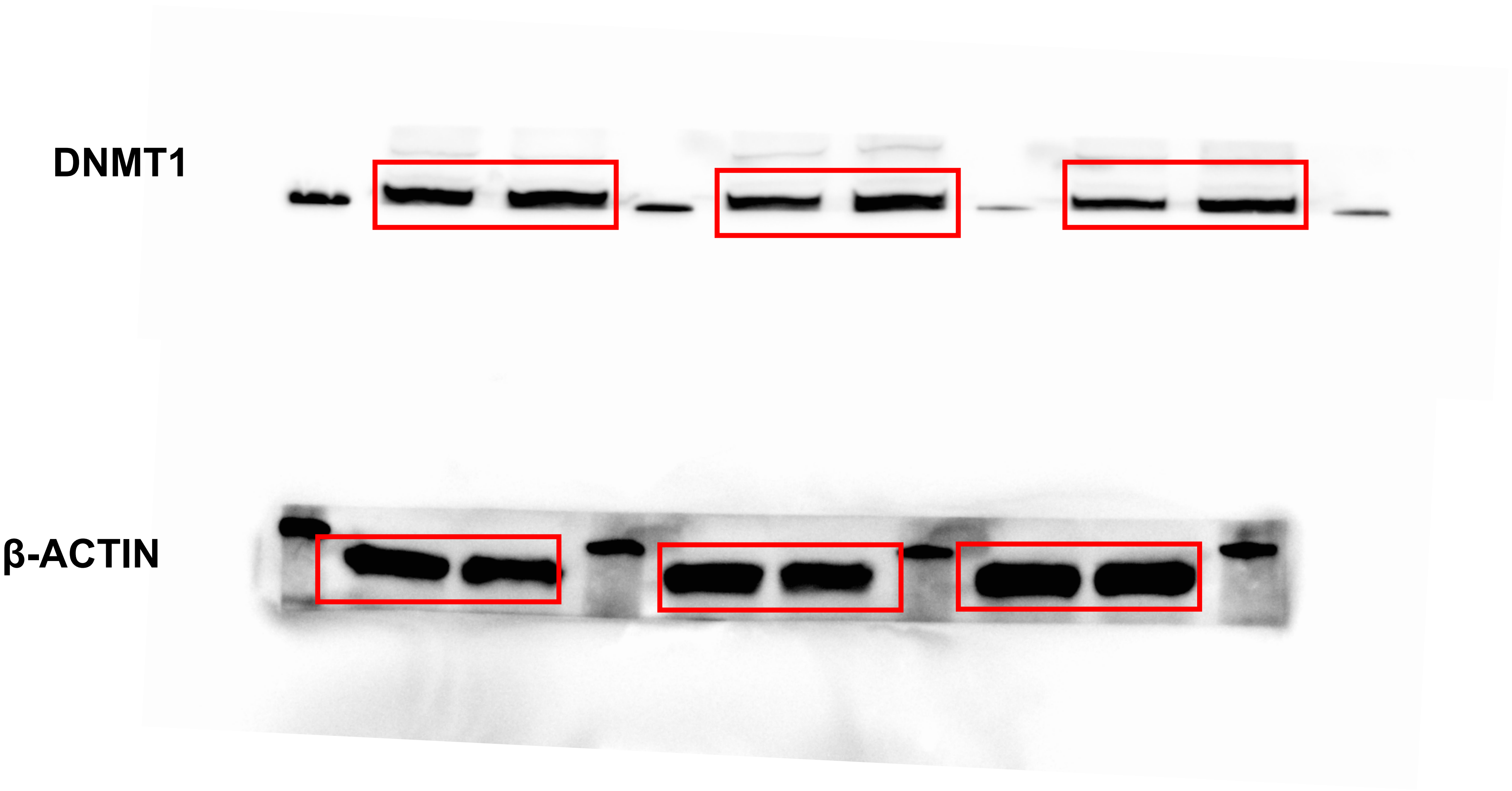

**Supplementary Figure 9 B**

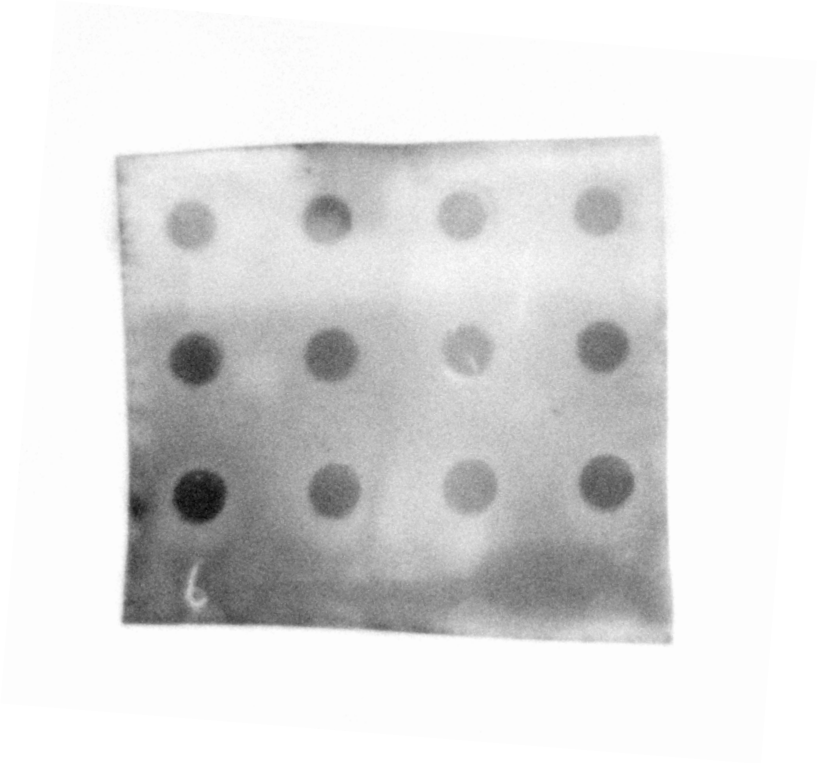

Supplementary
Figure 9 F

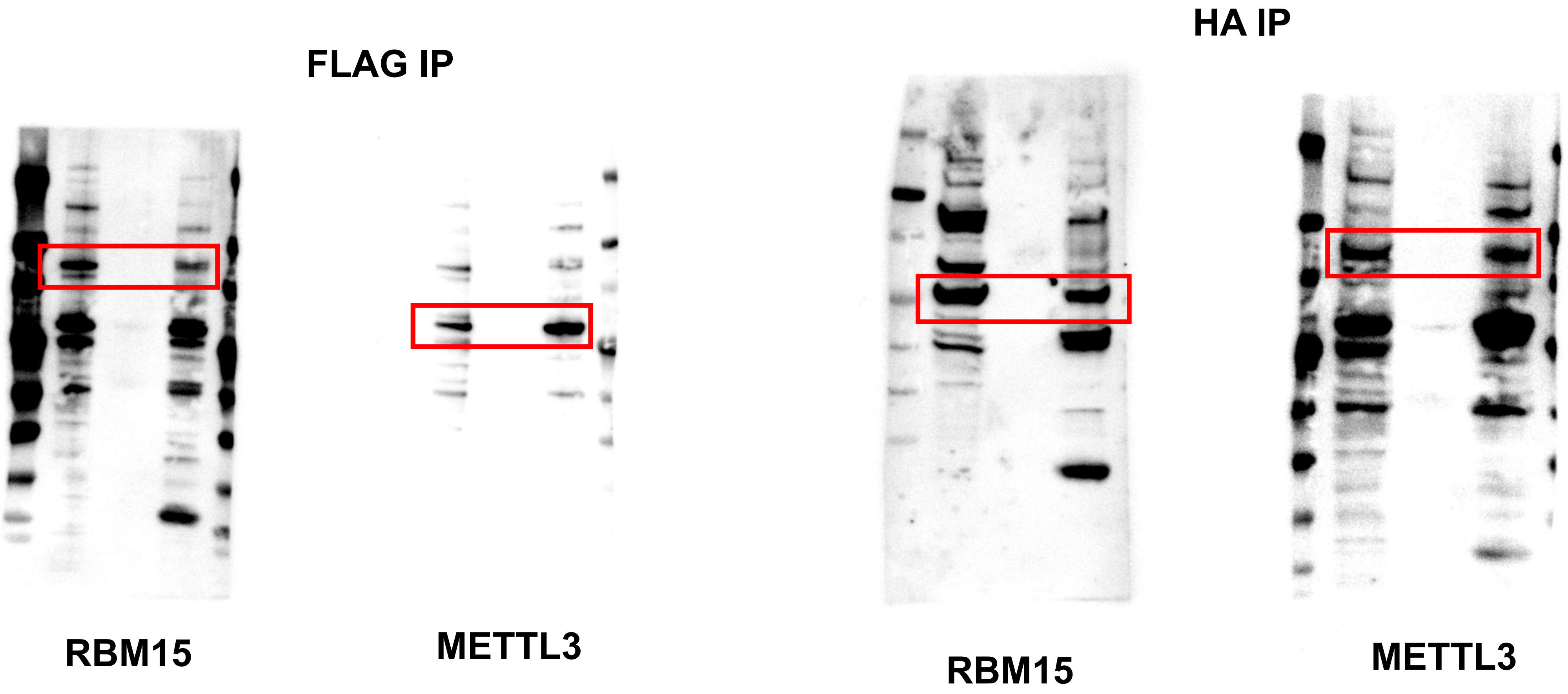

**Supplementary Figure 10 A**

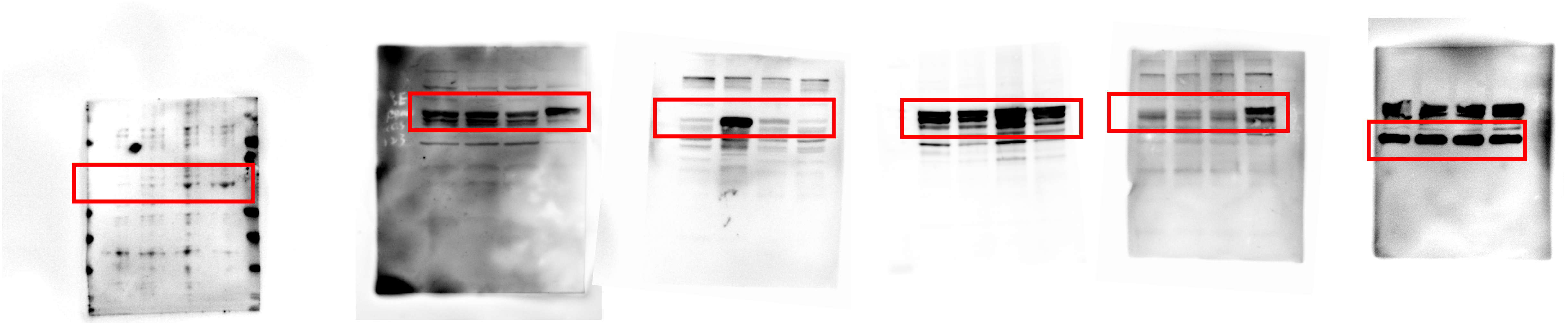

RBM15 SEMA3F IGF2BP1 IGF2BP2 IGF2BP3 β-ACTIN

Supplementary
Figure 10 B

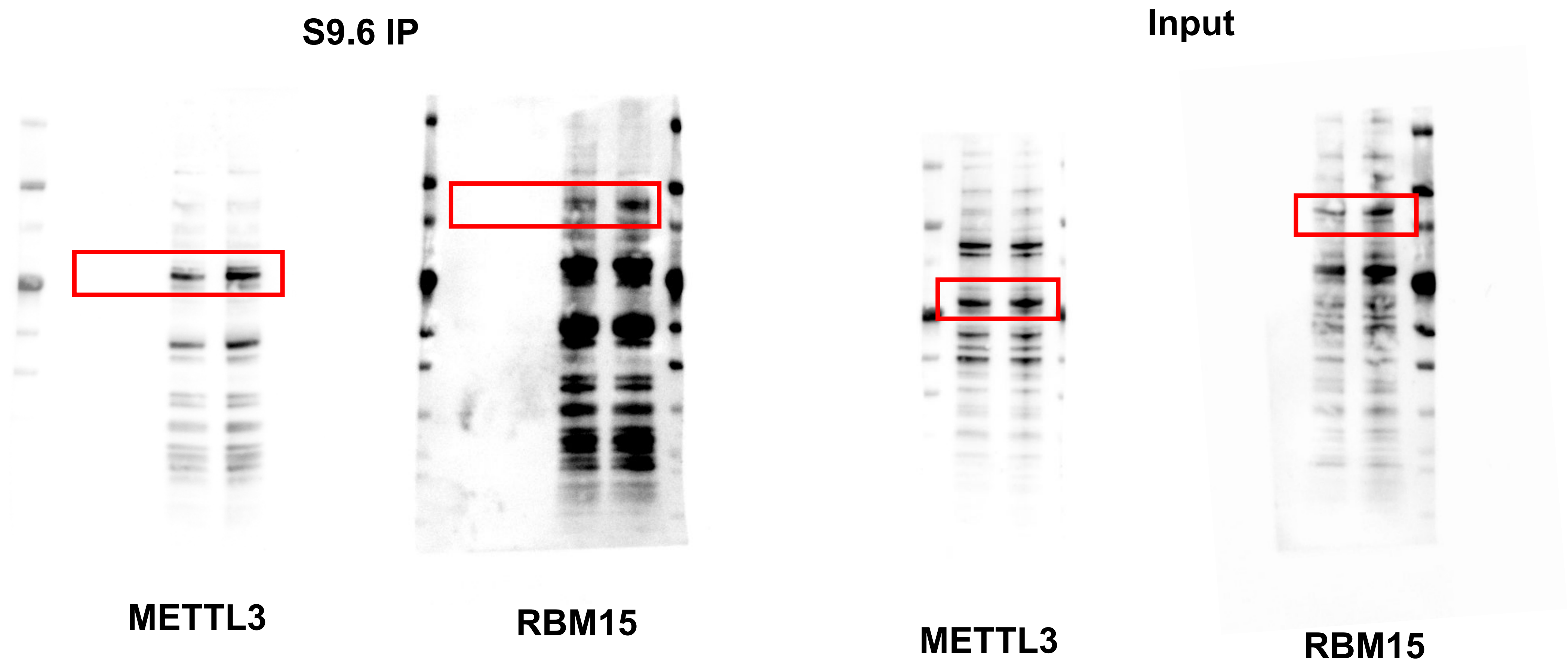

Supplementary
Figure 10 C

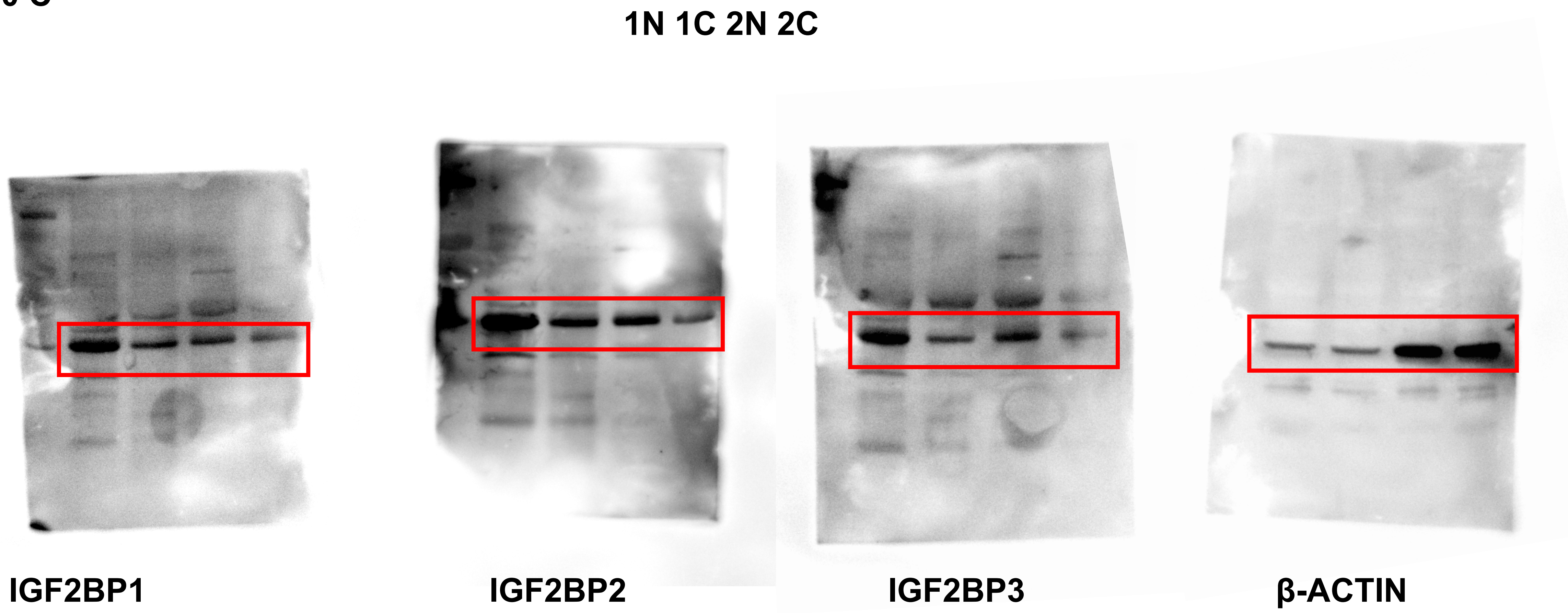

**Supplementary Figure 10 C**

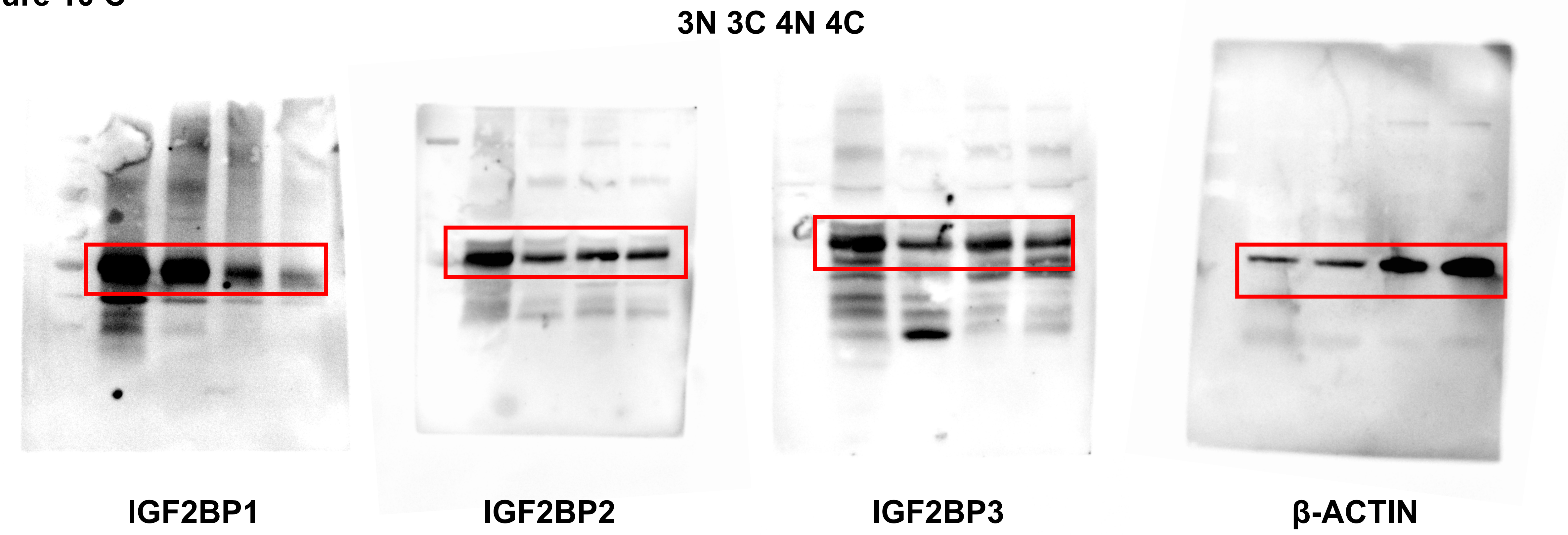

**Supplementary Figure 10 C**

5N 5C 6N 6C

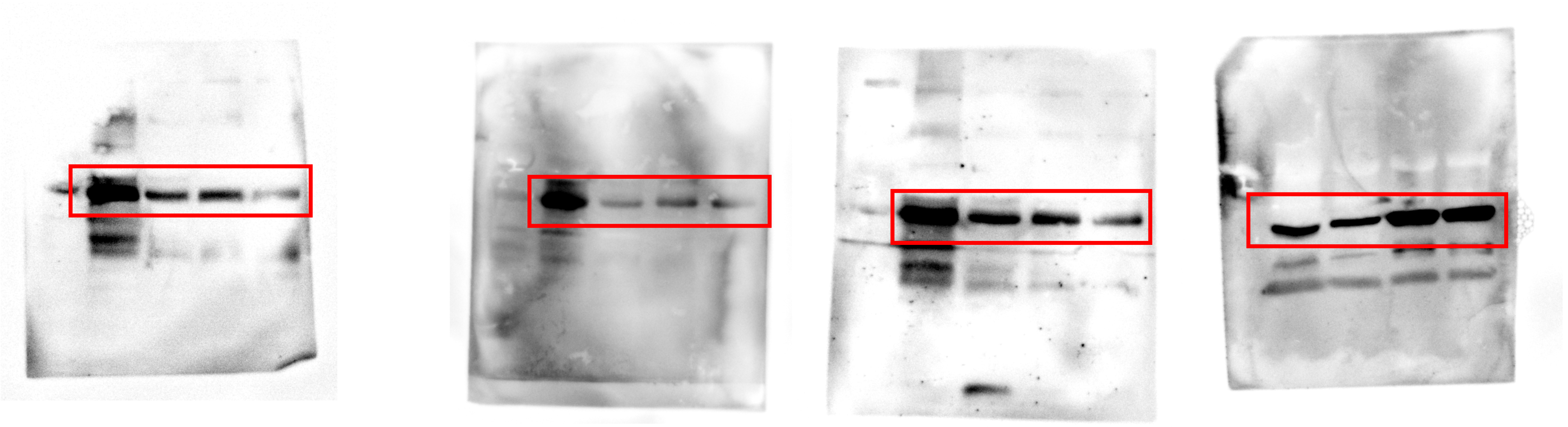

IGF2BP1 IGF2BP2 IGF2BP3 β-ACTIN

**Supplementary Figure 10 D**

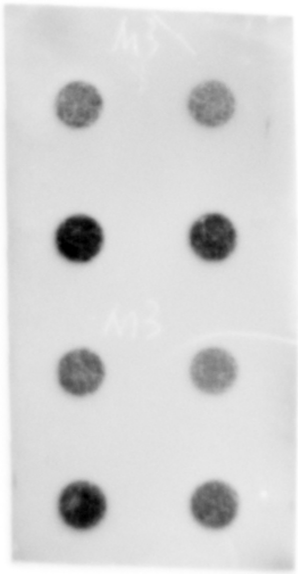

Supplementary
Figure 11E

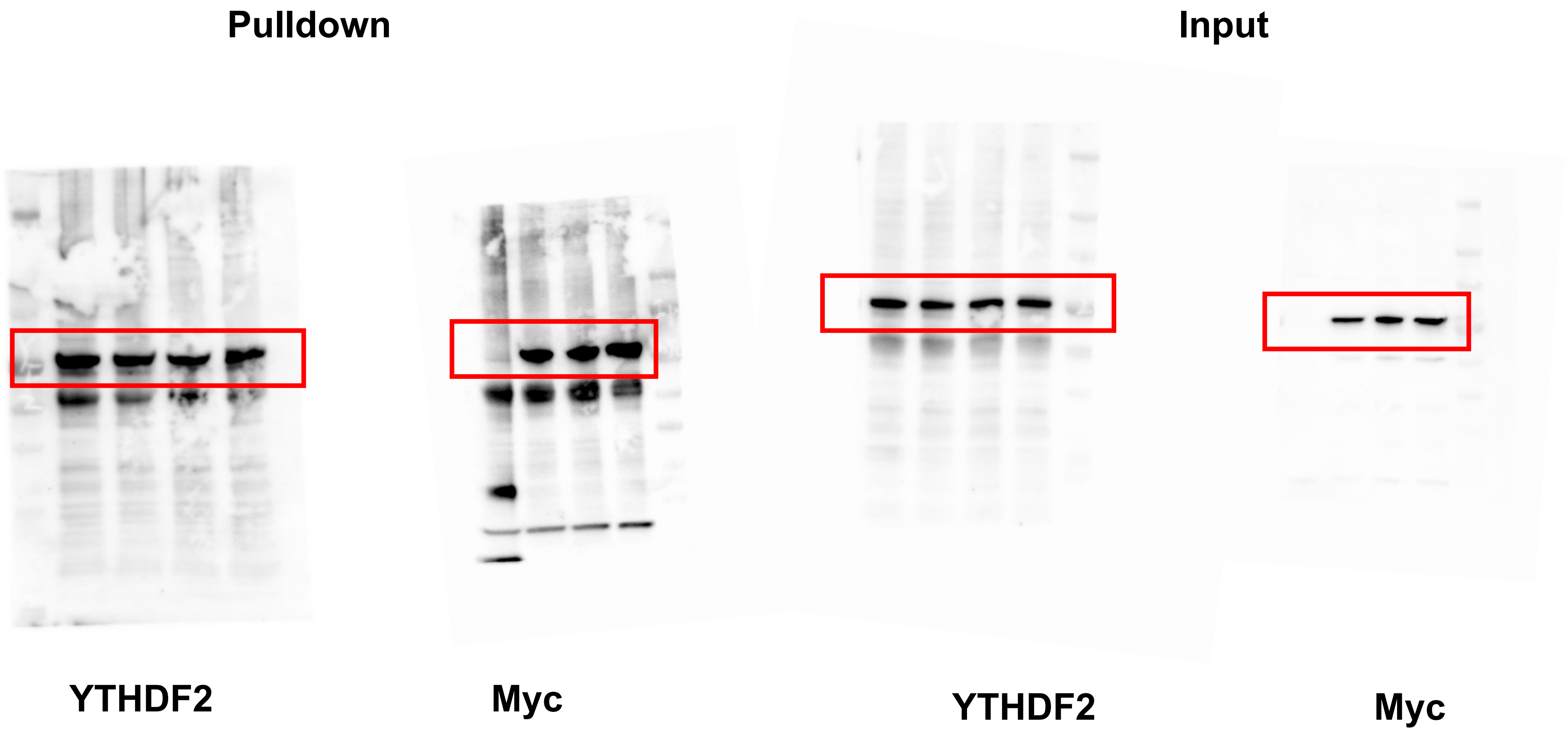

## Supplementary Figure 11 I

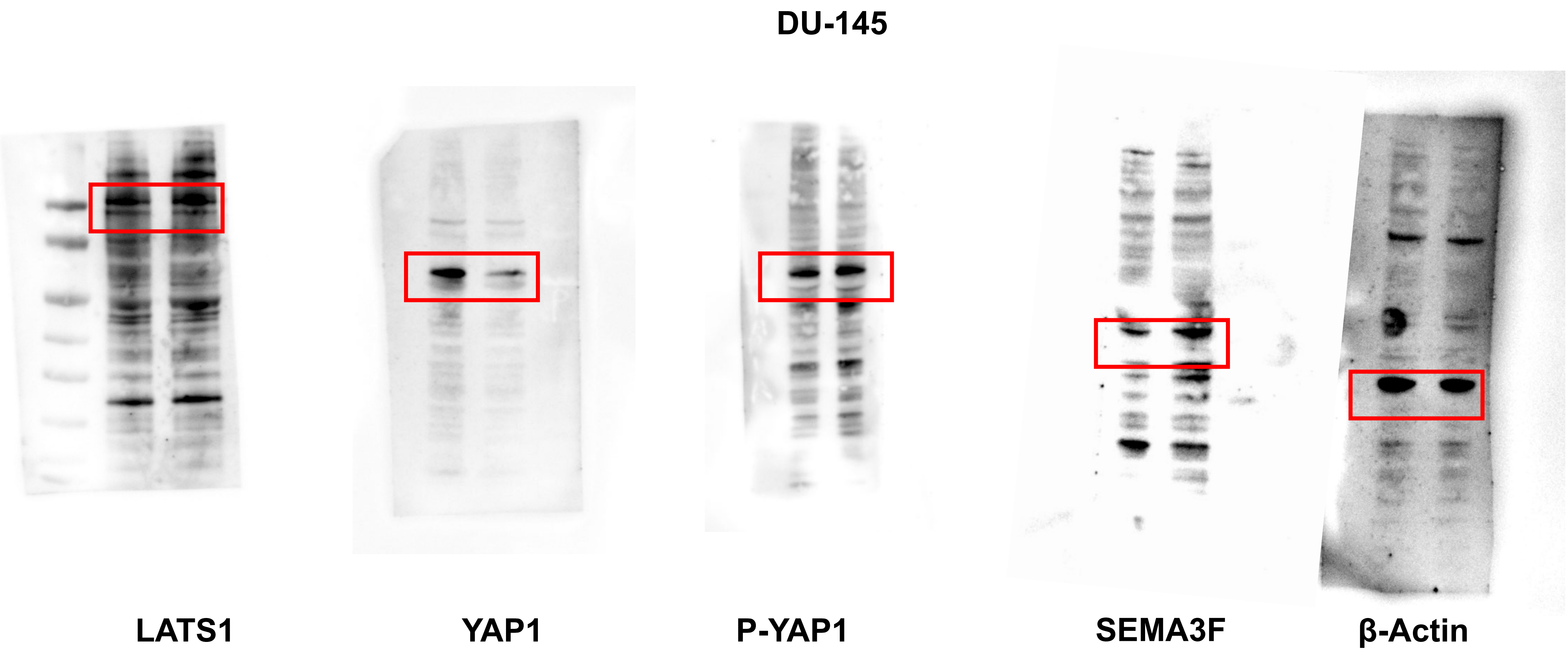

**Supplementary Figure 11 I**

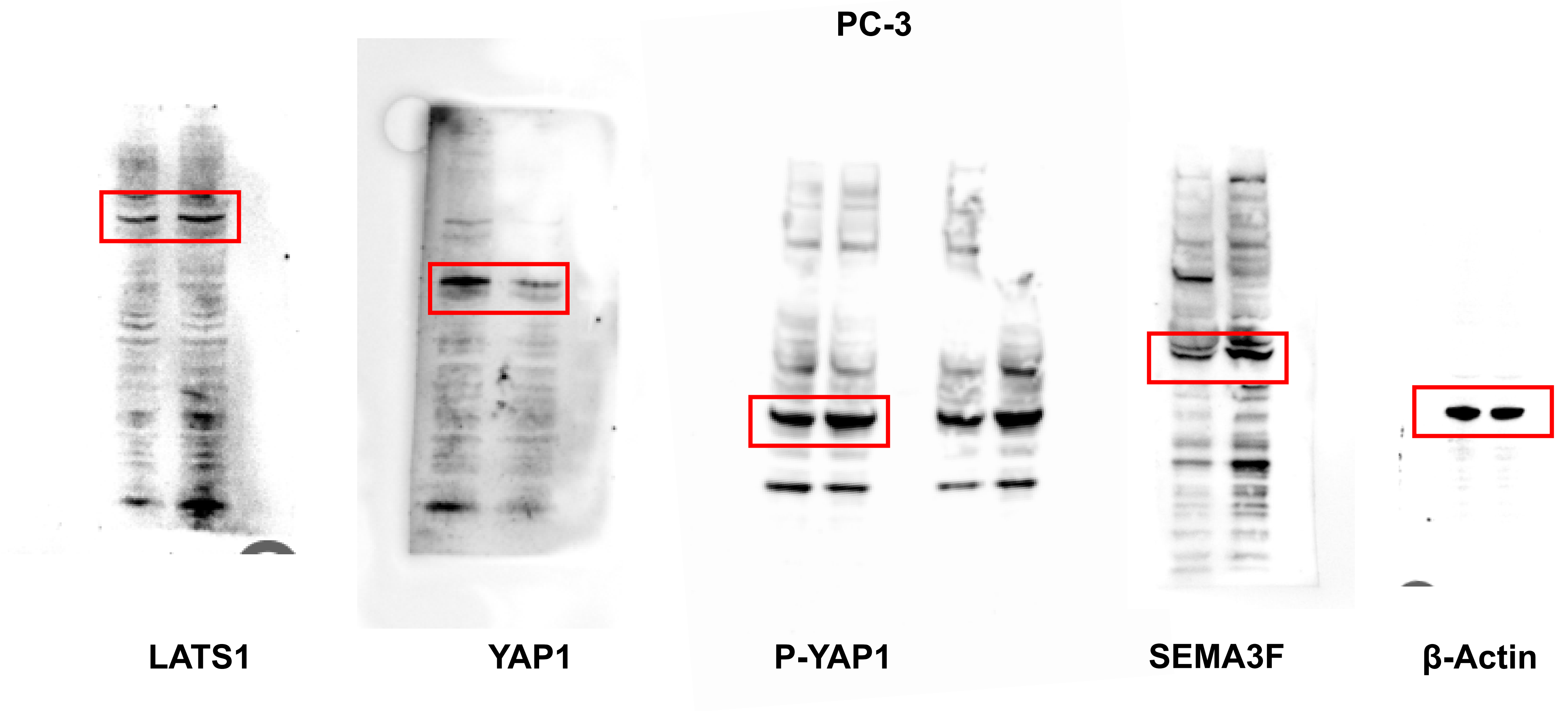

**Supplementary Figure 11 J**

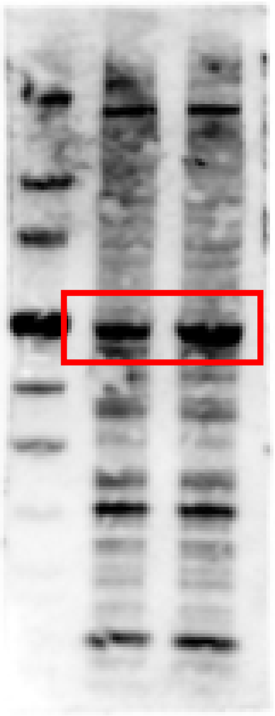

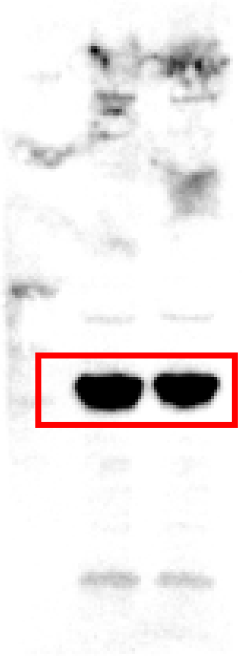

SEMA3F β-Actin
